# Comprehensive phenotypic characterization of an allelic series of zebrafish models of NEB-related nemaline myopathy

**DOI:** 10.1093/hmg/ddae033

**Published:** 2024-03-17

**Authors:** Lacramioara Fabian, Esmat Karimi, Gerrie P Farman, Jochen Gohlke, Coen A C Ottenheijm, Hendrikus L Granzier, James J Dowling

**Affiliations:** Genetics and Genome Biology Program, Hospital for Sick Children, 555 University Ave., Toronto, ON M5G 1X8, Canada; Department of Cellular and Molecular Medicine, University of Arizona, 1007 E. Lowell Street, Tucson, AZ 85724, United States; Department of Cellular and Molecular Medicine, University of Arizona, 1007 E. Lowell Street, Tucson, AZ 85724, United States; Department of Cellular and Molecular Medicine, University of Arizona, 1007 E. Lowell Street, Tucson, AZ 85724, United States; Department of Physiology, Amsterdam University Medical Center (location VUMC), De Boelelaan 1108, Amsterdam 1081 HZ, The Netherlands; Department of Cellular and Molecular Medicine, University of Arizona, 1007 E. Lowell Street, Tucson, AZ 85724, United States; Genetics and Genome Biology Program, Hospital for Sick Children, 555 University Ave., Toronto, ON M5G 1X8, Canada; Division of Neurology, Hospital for Sick Children, 555 University Ave., Toronto, ON M5G 1X8, Canada; Departments of Paediatrics and Molecular Genetics, University of Toronto, 1 King’s College Circle, Toronto, ON M5S 1A8, Canada

**Keywords:** nemaline myopathy, nebulin, zebrafish, muscle, alternative splicing

## Abstract

Nemaline myopathy (NM) is a rare congenital neuromuscular disorder characterized by muscle weakness and hypotonia, slow gross motor development, and decreased respiratory function. Mutations in at least twelve genes, all of each encode proteins that are either components of the muscle thin filament or regulate its length and stability, have been associated with NM. Mutations in Nebulin (NEB), a giant filamentous protein localized in the sarcomere, account for more than 50% of NM cases. At present, there remains a lack of understanding of whether NEB genotype influences nebulin function and NM-patient phenotypes. In addition, there is a lack of therapeutically tractable models that can enable drug discovery and address the current unmet treatment needs of patients. To begin to address these gaps, here we have characterized five new zebrafish models of NEB-related NM. These mutants recapitulate most aspects of NEB-based NM, showing drastically reduced survival, defective muscle structure, reduced contraction force, shorter thin filaments, presence of electron-dense structures in myofibers, and thickening of the Z-disks. This study represents the first extensive investigation of an allelic series of nebulin mutants, and thus provides an initial examination in pre-clinical models of potential genotype-phenotype correlations in human NEB patients. It also represents the first utilization of a set of comprehensive outcome measures in zebrafish, including correlation between molecular analyses, structural and biophysical investigations, and phenotypic outcomes. Therefore, it provides a rich source of data for future studies exploring the NM pathomechanisms, and an ideal springboard for therapy identification and development for NEB-related NM.

## Introduction

Nemaline myopathy (NM) is a rare congenital neuromuscular disorder [1], characterized by muscle weakness and hypotonia, slow gross motor development, and decreased respiratory function [2]. NM presents with a wide spectrum of clinical phenotypes, ranging from a neonatal severe form, which can lead to early mortality [3, 4], to early-childhood or adult-onset forms [5]. The defining pathological feature of NM is the presence of nemaline bodies in muscle fibers [6]. Nemaline bodies are electron-dense protein aggregates, most often rod-like shaped, distributed in the skeletal muscle [7], and containing disorganized Z-disk and thin filament proteins [3, 8]. Currently, there is no cure, nor any disease modifying therapies, for NM.

Mutations in at least twelve genes have been associated with NM [1, 9]. All these genes encode proteins that are either components of the muscle thin filament or are involved in regulation of actin filament length and stability [6]. Mutations in Nebulin (*NEB*) account for more than 50% of cases of NM [5]. The most common types of mutations identified in patients with NEB-related NM are splice-site and frameshift mutations, which account for more than 65% of cases [10]. Mutations are found anywhere along NEB, with no mutation hot spot/s identified thus far.

**Figure 1:**
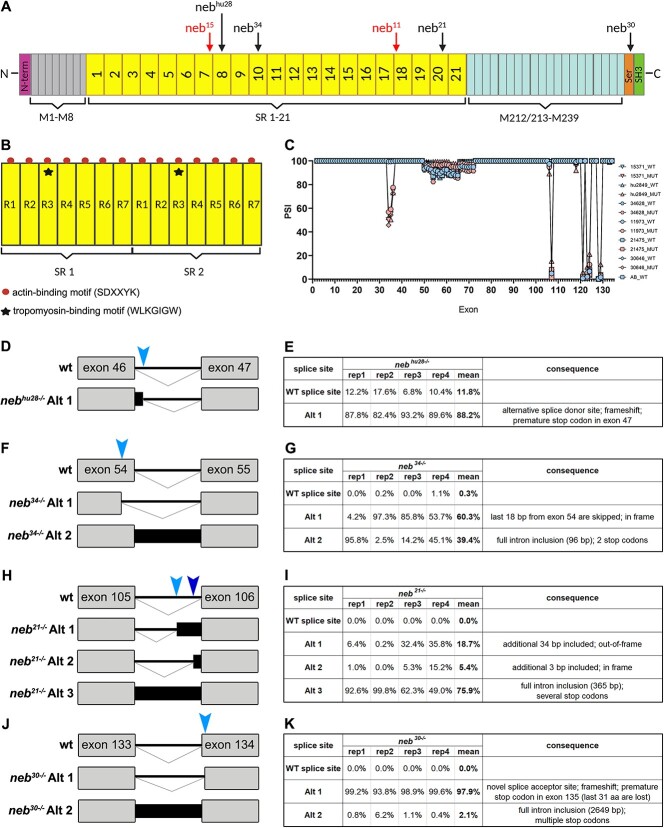
Zebrafish nebulin structure and nebulin mutations. (A) Diagram of zebrafish nebulin with position of mutations. N-terminus of nebulin consists of a glutamic acid rich sequence followed by several distinctive domains that mediate interactions with tropomodulin. Central region of nebulin has a repetitive, modular structure, and, in zebrafish, is organized into 21 super-repeats (SR). The C-terminus of nebulin consists of several linker modules, a serine-rich and an SH3 domains, which mediate interactions with proteins in the Z-disk. (B) Diagram of two SR. Each SR is made up of seven simple repeats (R1-R7) and contains a conserved WLKGIGW motif (troponin/tropomyosin binding site) in R3 (star). Each simple repeat contains an actin-binding motif (SDXXYK) (dots). (C) Percent spliced in index (PSI) showing alternative splicing in nebulin. Skipping of exons 33–37 was observed only in *neb* mutants, whereas splicing of exons 49–73 and 107 was observed in both wt and mutant zebrafish. Several splicing events were observed at the C-terminus of nebulin. (D–K) Splicing analysis and variants identified by RNAseq in zebrafish splice mutants. Four wild-type and four mutant replicates (rep1-rep4) have been analyzed and the PSI has been calculated for each. Light and darkblue arrowheads indicate the location of the alternative splice site/s used. Exons are represented by grey rectangles, introns by thick black lines, and intron inclusion by black rectangles. (D and E) Diagram (D) and table (E) illustrating alternative splicing events in *neb^hu28^* mutant. One splice variant was identified in *neb^hu28^* (alt 1), which was generated by the usage of an alternative splice donor site located in intron 46. This caused a partial intron retention, a frameshift and the formation of a stop codon in exon 47. (F and G) Diagram (F) and table (G) illustrating two alternative splicing events in *neb^34^* mutant (alt 1 and alt 2). In the alt 1 variant, an alternative splice donor site in exon 54 is used, which resulted in skipping the last 18 base pairs from exon 54, and generation of an in-frame transcript. In the alt 2 variant, the full intron was retained. (H and I) Diagram (H) and table (I) illustrating three alternative splicing events in *neb^21^* mutant (alt 1, alt 2, and alt 3). In the alt 1 and 2 variants, usage of two different alternative splice acceptor sites located in intron 105 resulted in addition of 34 base pairs and out-of-frame transcript (alt 1, light blue arrowhead), or in addition of 3 base pairs and in-frame transcript (alt 2, dark blue arrowhead). In the alt 3 variant, the full intron was retained. (J and K) Diagram (J) and table (K) illustrating two alternative splicing events in *neb^30^* mutant (alt 1 and alt 2). A new splice acceptor site was generated by the mutation, located just one base pair downstream of the original site (alt 1). This caused a frameshift and the formation of a premature stop in exon 135, which is the last exon in zebrafish nebulin. In the alt 2 variant, the full intron was retained.

Nebulin is a giant filamentous muscle protein (600- to 900-kDa), localized in the sarcomere, along actin filaments (reviewed in [11]). Nebulin structure consists of a repetitive and modular central region, organized into super repeats (SR), each made up of 7 simple repeats (R) (Fig. 1A and B). Each SR contains a conserved WLKGIGW motif, which represents the troponin/tropomyosin binding site (Fig. 1B), and each simple repeat contains an actin-binding motif (SDXXYK) (Fig. 1B) [11]. This structure allows for nebulin alignment along actin filaments in the sarcomere. The C-terminus of nebulin is found within the Z-disk of the sarcomere and consists of several linker modules, a Serine-rich and an SH3 domains, which mediate interactions with proteins in the Z-disk (Fig. 1A). The N-terminus of nebulin is found near the M line of the sarcomere, close to pointed end of actin filaments and consists of a glutamic acid rich sequence followed by several distinctive domains that mediate interactions with tropomodulin (Tmod) (reviewed in [11]).

To understand the role of nebulin in healthy and diseased muscle function, several NEB-related NM mouse and zebrafish animal models have been generated (reviewed in [11]). Results from studies using these animal models, which are primarily loss of expression mutants, revealed diverse functions for nebulin in the skeletal muscle. Nebulin was shown to be involved in thin filament length specification and stability [12–21], in sarcomere organization by regulating Z-disk width and stability [20, 22–24], in promoting alignment of myofibrils in muscle fibers [24–27], and in regulation of muscle contraction by influencing cross-bridge cycling, excitation-contraction coupling, and calcium homeostasis [27–34].

At present, there remains a lack of understanding of whether NEB genotype influences nebulin function and NEB patient phenotypes. In addition, there is a lack of therapeutically tractable models that can enable drug discovery and address the current unmet treatment needs of patients. To begin to address these gaps, we have characterized five new zebrafish models of NEB-related NM. These mutants recapitulate most aspects of NEB-based NM, showing drastically reduced survival, defective muscle structure with disorganized muscle fibers, misaligned and defective sarcomeres, presence of electron-dense structures in myofibers and thickening of the Z-disks. Importantly, they enable the first precise comparative evaluation of the consequences of different mutations. Overall, these zebrafish mutants showed impaired muscle function, with defective swimming behaviour, reduced contraction force and shorter thin filament length.

## Results

### *neb* zebrafish mutants

We characterized five new nebulin mutants, *neb^15^*, *neb^34^, neb^11^*, *neb^21^*, *neb^30^* (Fig. 1A; Table 1), which we obtained from the Zebrafish International Resource Center (ZIRC). We selected these zebrafish lines because of the type of mutation they harbor and its position in the *neb* gene. All mutations are located in the SR region of nebulin, except for *neb^30^*, in which the mutation is located in the serine-rich domain at the Z-disk localized C-terminus of *neb* (intron 133) (Fig. 1A). Two of these lines (*neb^15^* and *neb^11^*) have point mutations that create a premature stop codon (PTC, exons 45 and 89, respectively) (“nonsense mutants”) (Table 1; Supplemental Fig. 1), whereas the other three lines (*neb^34^, neb^21^, neb^30^*) have point mutations in splice sites (“splice mutants”) (Table 1). Additionally, we further characterized a previously published nebulin mutant [20], which we named here *neb^hu28^* (Table 1).

**Table 1:** Zebrafish nebulin mutants.

**ZIRC name**	**Abbreviation**	**Type of mutation**	**Location**	**Consequence**	**Splicing analysis**	**Transcript**	**Protein (Westerns)**
**sa15371**	**neb** ^**15**^	Point mutation G > T	exon 45; SR 7, R7	premature stop; non-sense mutant		reduced	drastically reduced
**hu2849**	**neb** ^**hu28**^	Point mutation C > T	intron 46; splice donor site; SR 8, R4	splice mutant	partial intron inclusion; use of alternative splice donor adds 5 bp (88.2%); frameshift; premature stop codon in exon 47	reduced	reduced
**sa34628**	**neb** ^**34**^	Point mutation C > T	intron 54; splice donor site; SR 10, R4	splice mutant	alt 1: use of alternative splice donor site results in last 18 bp being skipped from exon 54 (60.3%); in frame	unchanged	reduced
					alt 2: full intron inclusion (96 bp) (39.4%); + 2 stop codons		
**sa11973**	**neb** ^**11**^	Point mutation A > T	exon 89; SR 18, R1	premature stop; non-sense mutant		reduced	drastically reduced
**sa21475**	**neb** ^**21**^	Point mutation T > G	intron 105; splice acceptor site; SR 20, R5	splice mutant	alt 1: partial intron inclusion; use of alternative splice acceptor site 1 results in additional 34 bp to be included in exon 106 (18.7%); out-of-frame	unchanged	reduced
					alt 2: partial intron inclusion; use of alternative splice acceptor site 2 results in additional 3 bp to be included (5.4%); in frame		
					alt 3: full intron inclusion (365 bp) (75.9%); + several stop codons		
**sa30646**	**neb** ^**30**^	Point mutation C > T	intron 133; splice acceptor site; Serine-rich domain (Z-disk)	splice mutant	alt 1: use of novel alternative splice acceptor site generated by the mutation results in frameshit (97.9%); premature stop codon in exon 135	unchanged	slightly reduced
					alt 2: full intron inclusion (2649 bp) (2.1%); + several stop codons		

Note: Percentages in splicing analysis refer to usage of donor/acceptor sites near the mutation

We first evaluated the molecular consequence(s) of the mutations by RNA sequencing (RNAseq). Multiple splice changes were identified in each splice mutant. For each of them, the exon inclusion level [percentage spliced in (PSI, Fig. 1C)] and percentage splice site usage near the mutation (Table 1; Fig. 1D–K; Supplemental Figs 2–5) were calculated. For *neb^hu28^*, only one splice variant was identified (alt 1), which was generated by the usage of an alternative splice donor site located in intron 46. This causes partial intron retention, leading to a frameshift and the formation of a stop codon in exon 47 (Fig. 1D and E; Supplemental Fig. 2). For the *neb^34^* mutant, we identified two alternative splicing events: alt 1 (60.3% of transcripts), in which an alternative splice donor site in exon 54 is used, which results in skipping of the last 18 base pairs from exon 54 and generation of an in-frame transcript, and alt 2, which results in full intron retention and introduction of two stop codons (39.7% of transcripts) (Fig. 1F and G; Supplemental Fig. 3). For the *neb^21^* mutant, we identified three alternative splicing events: usage of an alternative splice acceptor site in intron 105 resulting in addition of 34 base pairs and frameshift and PTC (alt 1), usage of an alternative splice acceptor resulting in addition of 3 base pairs and an in-frame transcript (alt 2), and full intron retention with introduction of several stop codons (75.9% of transcripts) (Fig. 1H and I; Supplemental Fig. 4). In the *neb^30^* mutants, we identified two alternative splicing events: a new splice acceptor site located one base pair downstream of the original site that introduces frameshift and PTC in exon 135, the last exon in zebrafish nebulin (alt 1, 97.9% of transcripts), and full intron retention with introduction of multiple stop codons (alt 2, 2.1% of transcripts) (Fig. 1J and K; Supplemental Fig. 5).

RNAseq analysis also revealed several alternative splicing events in other regions of neb (i.e. not where the mutations are located) (Fig. 1C). In the SR region of *neb*, partial exclusion of exons 33-37 was observed only in *neb* mutants, whereas exclusion of exons 49-73 and 107 was observed in mutants and their wild-type siblings (Fig. 1C). Outside of the SR region, additional splicing events shared between mutants and wild types were observed in the C-terminus region (exons 121, 123/124, 128/129) (Fig. 1C).

### *neb* transcript and protein levels are reduced in some *neb* mutants

**Figure 2:**
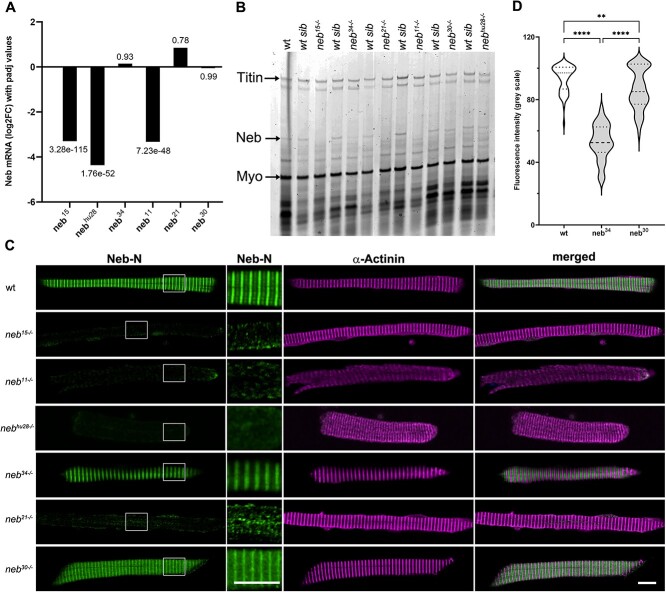
Nebulin expression is reduced in *neb* mutants. (A) Nebulin transcript levels, determined from the RNAseq data, were drastically reduced in the nonsense and *neb^hu28^* mutants, whereas the levels in the splice mutants were consistent with those in the wt siblings (numbers indicate the padj values). (B) SDS-agarose gel stained with Coomassie blue showing reduced levels of nebulin in all mutants. (C) Confocal micrographs of myofibers stained with antibodies against Neb-N and α-Actinin. The nonsense, *neb^21^,* and *neb^hu28^* mutants show altered localization and drastically reduced levels of nebulin. The localization pattern of nebulin in *neb^34^* and *neb^30^* splice mutants is similar to that in the wt myofibers; however, nebulin levels are significantly reduced. To better illustrate the altered localization of nebulin, the brightness in the region marked by the square in left column panels was increased to 150% in panels showing high magnification detail of Neb-N staining. Scale bars = 10 μm. (D) Quantification of fluorescence intensity of anti-Neb-N antibody staining in myofibers (mean ± SEM). Violin plot showing that fluorescence intensity is significantly reduced in *neb^34^* and *neb^30^* mutants compared to wt (56.5% in *neb^34^* and 92.7% in *neb^30^*, respectively). n = 56 measurements/genotype. Asterisks indicate *p*-values (** ≤ 0.01; **** ≤ 0.0001). See Supplemental Table 1 for fluorescence intensity value measurements and statistics.

Total nebulin transcript levels, as determined from the RNAseq data, were drastically reduced in the two nonsense mutants and in *neb^hu28^*, whereas the overall levels of the other splice mutants were not obviously reduced as compared to wild-type siblings (Fig. 2A; Table 1). Neb protein levels, as determined from SDS-agarose gels, were reduced in all *neb* zebrafish mutants (Fig. 2B; Table 1). Immunostaining of myofiber preparations with antibody against the N-terminus of nebulin (Neb-N) also showed reduced levels in all *neb* mutants (Fig. 2C and D). The nonsense mutants and the *neb^21^* splice mutant showed a drastic reduction in fluorescence intensity of nebulin staining (almost complete lack of staining) and altered distribution of nebulin in myofibers (Fig. 2C), whereas the *neb^34^* and *neb^30^* mutants showed fluorescence intensity reduced to 56.5% and 92.7% of the wild-type levels, respectively (Fig. 2D; Supplemental Table 1). In total, the protein expression data is consistent with our RNAseq, in that 60% of transcripts for *neb^34^* have an in-frame insertion and all transcripts for *neb^30^* only impact the final exon (and thus would not be subjected to nonsense-mediated decay).

### *neb* mutants show compromised muscle integrity and early mortality

**Figure 3:**
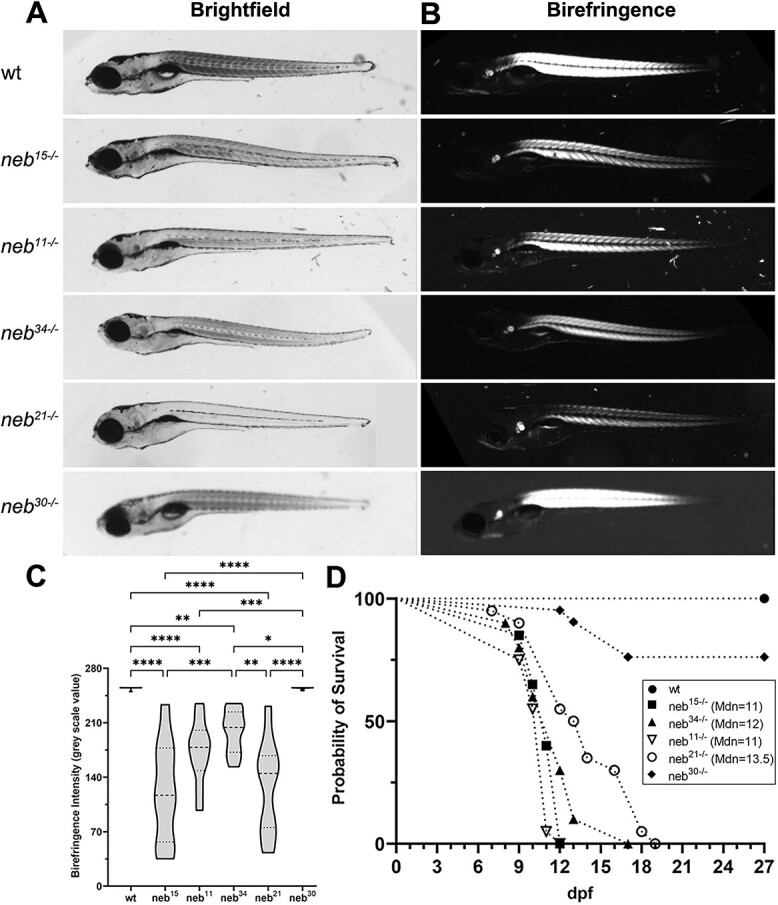
Morphological defects and early mortality in *neb* mutants. (A) Brightfield images of wt and *neb* mutant embryos at 6 dpf. All the *neb* mutants, except *neb^30^*, fail to inflate their swim bladder by 6 dpf, show defects in the lower jaw, and some mutants have bent body. (B) Polarized stereomicroscope images showing birefringence of skeletal muscle in zebrafish embryos at 6 dpf. All the *neb* mutants, except *neb^30^*, have reduced birefringence, indicating that muscle integrity and structure are compromised. (C) Quantification of birefringence intensity (mean ± SEM). All the *neb* mutants, except *neb^30^*, showed significantly reduced birefringence. Asterisks indicate *p*-values (* ≤ 0.05; ** ≤ 0.01; *** ≤ 0.001; **** ≤ 0.0001). (n = 8 for wt; n = 5 for each mutant). See Supplemental Table 2 for intensity value measurements and statistics. (D) Survival curve indicating drastically shorter life span in all the *neb* mutants, except *neb^30^*. (n = 20 for wt, *neb^15^*, *neb^11^, neb^21^*; n = 10 for *neb^34^;* n = 21 for *neb^30^*). Median survival: 11 days (*neb^15^*, *neb^11^*), 12 days (*neb^34^*), and 13.5 days (*neb^21^*).

The phenotype of the two nonsense mutants (*neb^15^* and *neb^11^*) is fully penetrant by 6 dpf (Fig. 3A). These embryos have defects in the lower jaw, show a bent body, fail to inflate their swim bladder, and have significantly reduced birefringence (Fig. 3B and C; Supplemental Table 2), indicating that muscle integrity and structure is compromised. The nonsense mutants begin dying by 8-9 dpf, and by 12 dpf all mutants are dead (Fig. 3D).

In keeping with the protein expression data, the phenotype of the splice mutants *neb^34^* and *neb^21^* share features with the nonsense mutants, including significantly reduced birefringence (Fig. 3A–C; Supplemental Table 2). The *neb^34^* mutant embryos start dying by 8 dpf and by 17 dpf all mutants are dead (Fig. 3D), whereas the *neb^21^* mutant embryos start dying by 7 dpf and by 19 dpf all mutants are dead (Fig. 3D). The phenotype of the splice mutant *neb^30^* is very mild, with bent body and lower jaw defects observed in a few embryos and birefringence was normal (Fig. 3A–C; Supplemental Table 2). Mutants live to adulthood and survival is only slightly reduced compared to wild type (Fig. 3D).

### *neb* mutants have impaired muscle function

**Figure 4:**
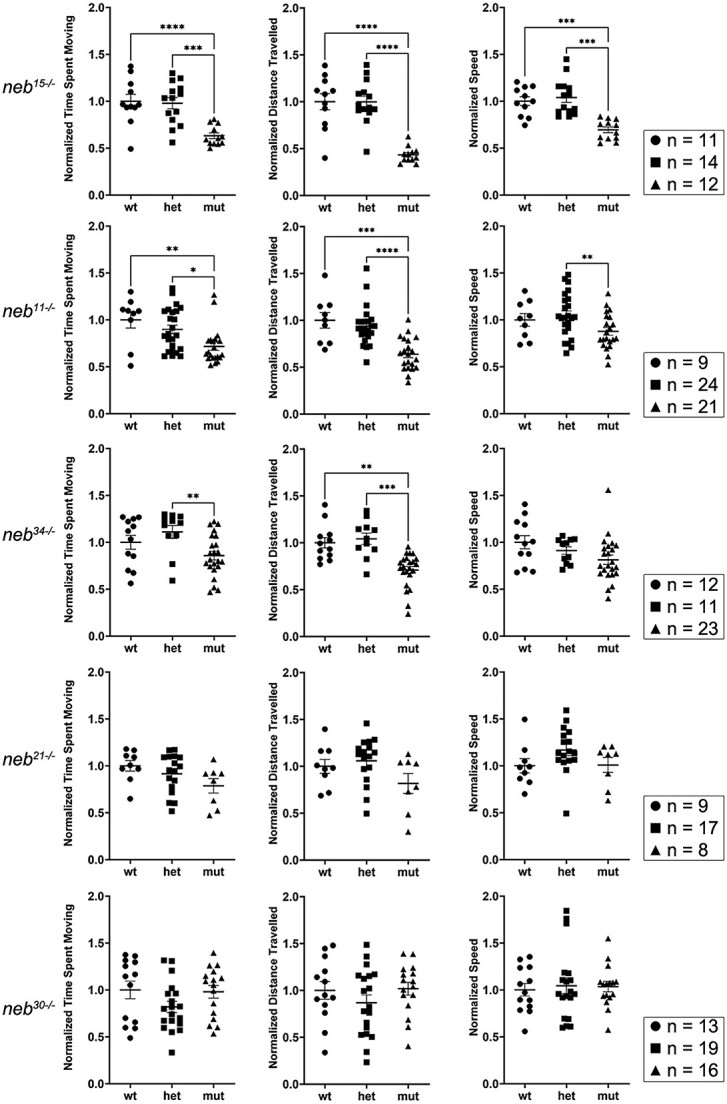
Swimming behaviour is defective in *neb* mutants. Muscle performance and function in *neb* mutants was assessed by tracking optovin-induced swimming of embryos at 7 dpf. Graphs showing time spent moving, distance travelled, and speed for each zebrafish line. The nonsense mutants *neb^15^* and *neb^11^* showed significantly shorter time swimming, distance travelled, and lower speed compared to their siblings. The splice mutants *neb^34^* and *neb^21^*, with mutations in SR region, also showed significantly shorter time spent swimming. The motor behaviour of the splice mutant *neb^30^* is not significantly different from that recorded for its siblings. Graphs display values normalized to wt (mean ± SEM). Asterisks indicate *p*-values (* ≤ 0.05; ** ≤ 0.01; *** ≤ 0.001; **** ≤ 0.0001). See Supplemental Table 3 for descriptive statistics.

We assessed muscle performance and function in the five new *neb* mutants by performing swim assays after stimulation with optovin, which induces reversible photoactivation of motor behaviour in zebrafish embryos [35] (Fig. 4; Supplemental Table 3). The nonsense mutants *neb^15^* and *neb^11^* showed significant defects in swimming behaviour starting at 6 dpf (data not shown) and fully penetrant by 7 dpf (Fig. 4). Splice mutants *neb^34^* and *neb^21^*, with mutations in the SR region, also showed defective swimming behaviour at 7 dpf (Fig. 4), with some of the *neb^34^* mutants (those with bent body), swimming in circles. In general, we observed a higher degree of variation in these mutants compared to nonsense mutants. *neb^34^* showed significantly shorter time and distance travelled, whereas changes in *neb^21^* did not reach statistical significance. Swim performance in the splice mutant *neb^30^* was not significantly different from their wild-type siblings (Fig. 4).

### Muscle and sarcomere organization and structure are disrupted in *neb* mutants

**Figure 5:**
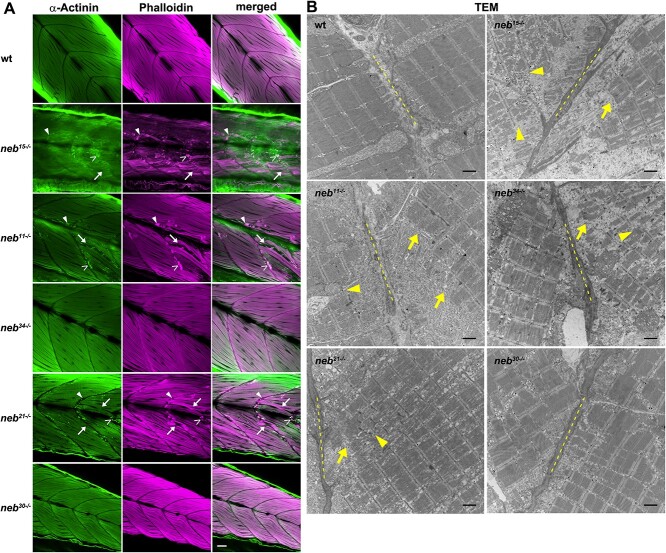
Muscle and sarcomere organization is defective in *neb* mutants. (A) Confocal micrographs of wholemount preparations of embryos at 6 dpf stained with antibody against α-Actinin and Rhodamine Phalloidin. Nonsense mutants and the splice mutant *neb^21^* show disorganized, misaligned or broken muscle fibers (arrows), defects in the MTJ regions, with partially detached fibers and accumulations of aggregates of actin filaments (arrowheads), and aggregates of actin/α-Actinin (open arrowheads). Scale bar = 20 μm. (B) Transmission electron micrographs of zebrafish embryos at 6 dpf showing defects in the MTJ regions (dotted line), with disorganized muscle fibers (arrows) and misaligned sarcomeres (arrowheads) in the nonsense, *neb^34^*, and *neb^21^* splice mutants. Scale bars = 2 μm.

To examine overall muscle and sarcomere organization, we performed wholemount staining with phalloidin and with an anti-α-Actinin antibody at 6 or 7 dpf. In both nonsense mutants and the splice mutant *neb^21^*, we observed disorganized, misaligned, and/or broken muscle fibers (Fig. 5A, arrows). We also observed defects in the myotendinous junction (MTJ) regions, with partially detached fibers and accumulation of aggregates of actin filaments (Fig. 5A, arrowheads). In addition, in these mutants, we observed aggregates of actin/α-Actinin, suggestive of nemaline bodies (Fig. 5A, open arrowheads). These results are consistent with the reduced birefringence observed in these mutants. No defects in muscle and sarcomere organization were observed in wholemount preparations of splice mutants *neb^34^* and *neb^30^*.

**Figure 6:**
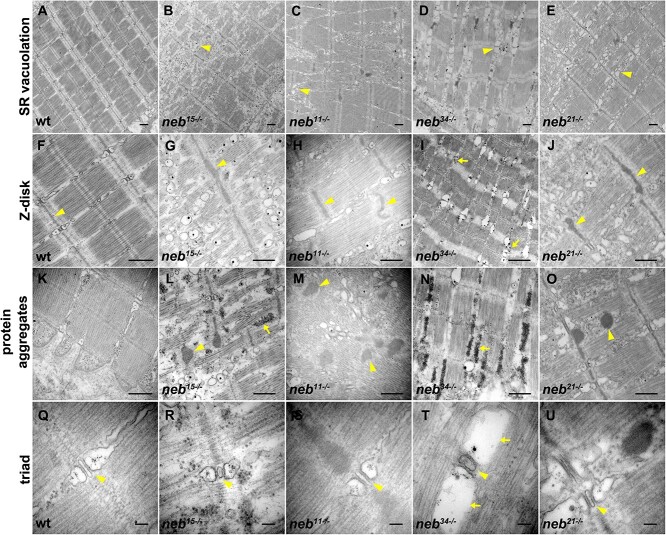
Details of ultrastructural defects in *neb* mutants. (A–U) Transmission electron micrographs of zebrafish embryos at 6 dpf. (A) Aligned sarcomeres in wt muscle. (B–E) Increased vacuolation of the sarcoplasmic reticulum in *neb^15^*, *neb^11^, neb^34^*, *neb^21^* mutants (arrowheads). (F) Z-disks in wt muscle (arrowheads). (G–J) Significantly thicker Z-disks in *neb^15^*, *neb^11^, neb^34^*, *neb^21^* mutants (arrowheads). (K) No abnormal protein aggregates have been observed in wt muscle. (L, M, and O) Electron-dense structures with a fibrous composition were observed in *neb^11^*, *neb^15^*, *neb^21^* (arrowheads). (L and N) Accumulation of ribosomes were observed in *neb^15^* and *neb^34^* mutants (arrows). (Q) Normal triad structure and organization in wt muscle (arrowhead). (R–U) Triad structure and organization did not seem to be defective in *neb* mutants (arrowheads), except in splice mutant *neb^34^*, where enlarged terminal cisternae were observed (arrows). Scale bars A-O = 500 nm. Scale bars Q-U = 100 nm.

To further characterize muscle defects, we performed transmission electron microscopy (TEM) studies on all mutants. These results confirmed that sarcomere structure is defective in the nonsense and *neb^21^* mutants. They also revealed defects in the splice mutant *neb^34^* that were not detectable in the wholemount stained preparations. Overall, we found that the MTJ area (Fig. 5B, dotted line) was disorganized, with many fibers not reaching all the way to the membrane [*neb^11^, neb^15^* (most), *neb^34^* (least), *neb^21^*] (Fig. 5B, arrows). We observed increased disorganization of myofibers [*neb^11^, neb^15^* (extensive), *neb^34^* (some)], regions with misaligned Z-disks [*neb^11^, neb^15^*, *neb^34^*, *neb^21^*] (Fig. 5B, arrowheads), increased vacuolation of the sarcoplasmic reticulum [*neb^15^*, *neb^11^, neb^34^*, *neb^21^*] (Fig. 6B–E; arrowheads), thickening of the Z-disk [*neb^15^*, *neb^11^, neb^34^ (least)*, *neb^21^*] (Fig. 6G, H and J; arrowheads), and Z-disk disassembly [*neb^34^*] (Fig. 6I, arrows). In addition, electron-dense structures were present in *neb^11^* (extensive), *neb^15^* (extensive), *neb^34^*, and *neb^21^* mutants (Fig. 6L–O). In some mutants, these electron-dense structures had a fibrous composition [*neb^15^*, *neb^11^, neb^21^*] (Fig. 6L, M, and O; arrowheads), whereas in others they consisted of accumulation of ribosomes [*neb^15^*, *neb^34^*] (Fig. 6L and N; arrows). Lastly, we looked at the triad structure and organization in these mutants. Overall, triad structure did not appear abnormal (Fig. 6R, S, and U; arrowheads), except in splice mutant *neb^34^*, where we observed increased vacuolation of the terminal cisternae (Fig. 6T; arrows).

### Force production during muscle contraction is impaired in *neb* mutants

**Figure 7:**
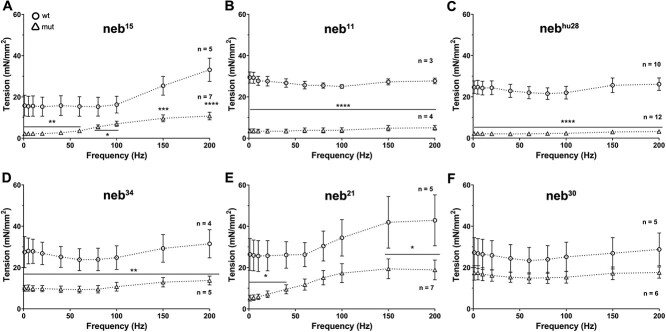
Force production is reduced in *neb* mutants. (A–F) Plots of force production at optimal length (mean ± SEM). Across all frequency range of stimulation (1–200 Hz), all of the mutants showed lower force production than the wt controls at optimal sarcomere length. The reduction in force production is significant in both nonsense mutants and the splice mutants *neb^hu28^* and *neb^34^*. Asterisks indicate *p*-values (* ≤ 0.05; ** ≤ 0.01; *** ≤ 0.001; **** ≤ 0.0001). See Supplemental Table 4 for measurements and statistics.

To examine the impact of nebulin mutations on force production, we performed muscle physiological studies in 4 dpf wild-type and mutant fish. Larvae were stimulated over a large range of frequencies (1–200 Hz) at optimal sarcomere length. All mutants (except *neb^30^)* showed lower force production than their wild-type siblings (Fig. 7A–F; Supplemental Table 4). A significant reduction in force production at all frequencies was observed only in *neb^15^, neb^34^, neb^hu28^,* and *neb^11^*, whereas in *neb^21^* the force was altered at all data points but only significantly reduced at the lowest (1–40 Hz) and highest (150 and 200 Hz) frequencies. The mean force levels in *neb^30^* mutant were lower than in the wild-type siblings across all frequencies but the differences did not reach statistical significance.

To assess the impact of nebulin mutations on the kinetics of force production, we measured activation and relaxation kinetics of single twitches produced at optimal sarcomere length and at 1 Hz frequency. Activation kinetics measures the time required for peak force development, starting at 10% force, while relaxation kinetics measures the time from peak force to 10% force [36]. We found no significant difference in either activation or relaxation kinetics between wild types and mutants across all strains, except for *neb^21^*, which showed a significant increase in relaxation kinetics in mutants relative to the wild type (Supplemental Fig. 6; Supplemental Table 5).

### *neb* mutants have shorter thin filaments

**Figure 8:**
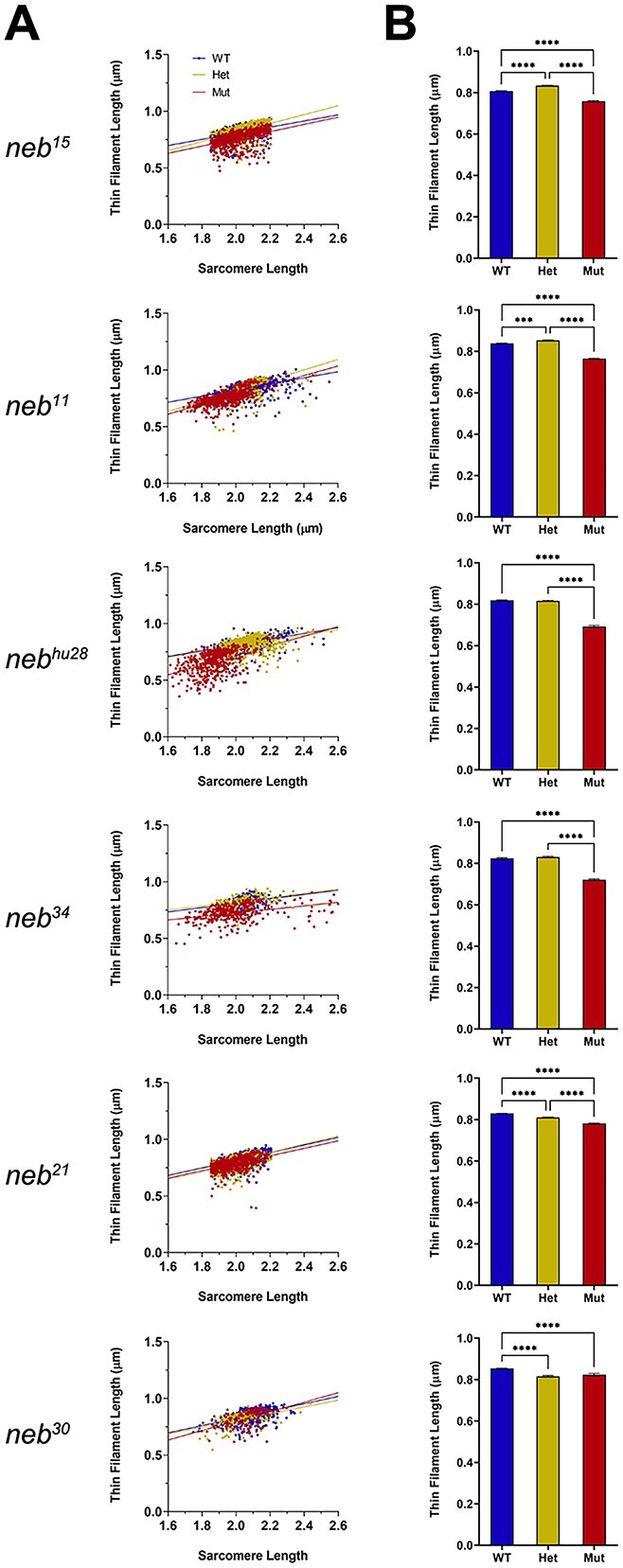
Thin filaments are shorter *neb* mutants. (A) Linear regression graphs of thin filament lengths measured at 1.6–2.6 μm sarcomere lengths. Thin filament length was reduced in all *neb* mutants compared to wt siblings. (B) Graphs of thin filament lengths measured at 1.85–2.2 μm sarcomere length (mean ± SEM). Thin filament length was significantly reduced in all *neb* mutants. Asterisks indicate *p*-values (* ≤ 0.05; ** ≤ 0.01; *** ≤ 0.001; **** ≤ 0.0001). See Supplemental Table 6 for statistics.

The length of the thin filament is a key determinant of muscle force generation [37], and nebulin has been shown previously to be a critical determinant of thin filament length [21]. We thus measured thin filament length in each mutant using TRITC-phalloidin staining and compared values to their wild-type and heterozygous siblings. Consistent with mouse Neb mutants [12], and as previously reported for *neb^hu28^* [20], thin filament length was significantly reduced in each group of mutants (Fig. 8; Supplemental Table 6). Unsurprisingly, the two nonsense mutants (which produce no detectable nebulin protein) had the highest magnitude reduction, though *neb^34^* (where levels were reduced to 40% of wild type) also was found to have a similarly large change. Conversely, *neb^30^* had the smallest reduction, though interestingly, still showed a significant shortening as compared to its wild-type siblings. Overall, the magnitude of reduction in thin filament length in each mutant correlated to the overall change in maximally activated tension observed for that mutant.

### *neb* mutants show altered transcriptome profile

**Figure 9:**
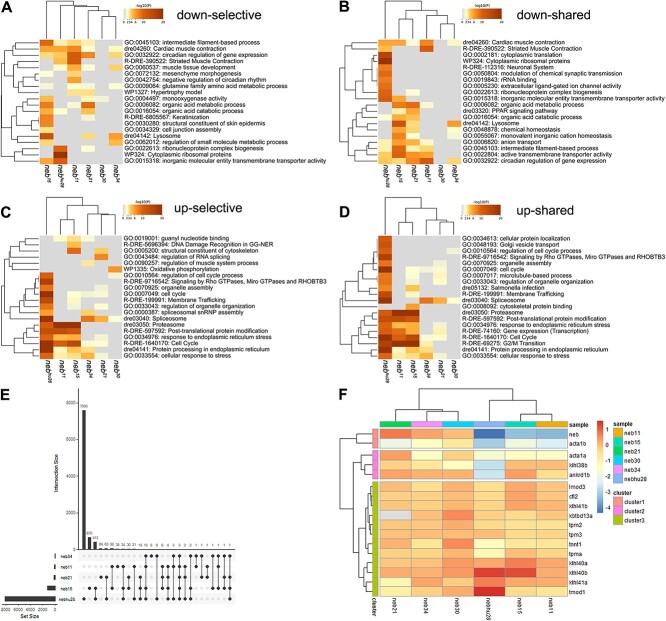
*neb* mutants show altered transcriptome profile. (A–D) Dendrograms and heatmaps from Metascape enrichment analysis illustrating selective (unique to each comparison in the study) and shared (common to all the comparisons in the study) enriched terms. (A) Selective downregulated pathways. (B) Shared downregulated pathways. Many of the downregulated genes in the nonsense mutants and the splice mutants *neb^hu28^* and *neb^21^* are involved in muscle contraction, muscle tissue development, and intermediate filament-based processes. (C) Selective upregulated pathways. (D) Shared upregulated pathways. Highly upregulated genes in the nonsense mutants are mostly involved in protein degradation processes and response to stress, and cluster under the proteasome and stress response pathways. The heatmap cells are coloured by their *p*-values; white cells indicate the lack of enrichment for that term in the corresponding gene list. *p*-value cutoff = 0.05; minimum enrichment = 1.5; minimum overlap = 3; kappa score = 0.3. (E) UpSet plot illustrating number of DEGs unique and shared between the data sets. The nonsense mutants and *neb^hu28^* cluster together. Only three DEGs are shared by all five neb mutants depicted in the plot. (F) Dendrogram and heatmap illustrating expression of nemaline myopathy genes in nebulin mutants. Noticeable, *acta1* and *tnnt1* are downregulated in several of the mutants investigated in this study. See Supplemental Table 7 for recorded values.

To better understand the molecular consequences of nebulin mutations, we performed whole animal comparative transcriptomic profiling using RNAseq. The number of differentially expressed genes (DEGs) varied, with the highest number being observed in the *neb*^*hu28*^ and *neb*^*15*^ mutants (> 1000 DEGs), whereas the smallest number of DEGs was observed in the *neb^30^* mutant (only 4 DEGs) (Table 2). Enrichment analysis showed that many of the downregulated genes that were shared between mutants with complete/near complete loss of nebulin (i.e. the nonsense mutants and the splice mutants *neb*^*hu28*^ and *neb*^21^) are involved in muscle contraction, muscle tissue development, and intermediate filament-based processes (Fig. 9A and B). Highly upregulated genes in the nonsense mutants and in *neb^hu28^* mostly involve protein degradation processes and response to stress, and cluster under the proteasome and stress response pathways (Fig. 9C and D). Interestingly, the spliceosome is highly upregulated in the splice mutants (Fig. 9C and D). Comparative analysis of DEGs in our data sets showed that only three genes are shared by all five *neb* mutants depicted in the plot (Fig. 9E).

**Table 2:** Number of DEGs in neb zebrafish mutants.

**Lines**	**DEGs (padj ≤ 0.05)**		
	**Up**	**Down**	**Total**
***neb*** ^***15***^	520	725	1245
***neb*** ^***11***^	96	75	171
***neb*** ^***hu28***^	4127	4049	8176
***neb*** ^***34***^	20	20	40
***neb*** ^***21***^	49	151	200
***neb*** ^***30***^	9	3	12

We examined in greater detail the expression of several genes that have been shown to be involved in nemaline myopathy [38], and found that *acta1* and *tnnt1* are drastically downregulated in several of the mutants (Fig. 9F; Supplemental Table 7). We also studied a multitude of muscle-relevant genes, specifically nebulin interactors and regulators. We found significant downregulation of several genes involved either in muscle fiber organization and development (i.e. *acta1, actc1, capza1, myhc, myhz1, desmb, tnni1*, etc.) or in muscle contractile dynamics (i.e. *atp2a2a*) (Supplemental Fig. 7; Supplemental Table 8). Interestingly, the only gene that was significantly upregulated in all mutants was *mustn1b*, which plays a critical role in myogenesis in vertebrates [39] (Supplemental Fig. 7; Supplemental Table 8).

## Discussion

In this study, we characterized five new zebrafish *nebulin* mutants and expanded the analysis of a previously published zebrafish *neb* mutant [20] (Table 3). This represents the first extensive investigation of an allelic series of nebulin mutants, and thus provides an examination in pre-clinical models of potential genotype-phenotype correlations in human NEB patients. It also represents the first utilization of a set of comprehensive outcome measure parameters in the zebrafish, including correlation between molecular analyses, structural investigations, biophysical studies, and phenotypic outcomes. As such, it provides a template for the study of other zebrafish *neb* mutants and animal models of human muscle diseases. Importantly, these newly characterized *neb* mutants share fundamental features of human NEB-related nemaline myopathy, including altered thin filament length, the presence of nemaline bodies, muscle weakness and impaired motor performance, and reduced survival [10, 40, 41]. As such, they therefore provide a rich source of data for future studies exploring the pathomechanism(s) related to NEB mutations, and an ideal springboard for therapy identification and development for NEB-related nemaline myopathy.

**Table 3:** Summary of results.

**Line**	**Splicing analysis**	**Birefringence**	**Swimming behaviour**	**Neb-N in myofibers**	**Muscle & sarcomere organization**	**Contraction Force**	**Thin Filament Length**	**Survival**
**neb** ^**15**^		reduced (48%)	swim defects (distance: 43.4%)	reduced (~none)	protein aggregates; thick and misaligned Z-disks; sarcoplasmic reticulum vacuolation; accumulation of ribosomes;	significantly reduced (33%)	significantly shorter (94%)	dead by 12 dpf
**neb** ^**hu28**^	partial intron inclusion; use of alternative splice donor adds 5 bp (88.2%); frameshift; premature stop codon in exon 47	reduced (Telfer *et al*. [20])	swim defects (Telfer *et al*. [20])	reduced (~none)	protein aggregates	significantly reduced (12%)	significantly shorter (85%)	dead by 7 dpf (Telfer *et al*. [20])
**neb** ^**34**^	alt 1: use of alternative splice donor site results in last 18 bp being skipped from exon 54 (60.3%); in frame	reduced (78%)	swim defects (distance: 70.9%)	reduced (56.5%)	normal by WM; misaligned Z-disks; sarcoplasmic reticulum vacuolation; accumulation of ribosomes;	significantly reduced (39%)	significantly shorter (88%)	dead by 17 dpf
	alt 2: full intron inclusion (96 bp) (39.4%); + 2 stop codons							
**neb** ^**11**^		reduced (68%)	swim defects (distance: 63.8%)	reduced (~none)	protein aggregates; thick and misaligned Z-disks; sarcoplasmic reticulum vacuolation;	significantly reduced (18%)	significantly shorter (91%)	dead by 12 dpf
**neb** ^**21**^	alt 1: partial intron inclusion; use of alternative splice acceptor site 1 results in additional 34 bp to be included in exon 106 (18.7%); out-of-frame	reduced (51%)	swim defects (distance: 81.8%)	reduced (~none)	protein aggregates; thick and misaligned Z-disks; sarcoplasmic reticulum vacuolation;	significantly reduced (44%)	significantly shorter (94%)	dead by 19 dpf
	alt 2: partial intron inclusion; use of alternative splice acceptor site 2 results in additional 3 bp to be included (5.4%); in frame							
	alt 3: full intron inclusion (365 bp) (75.9%); + several stop codons							
**neb** ^**30**^	alt 1: use of novel alternative splice acceptor site generated by the mutation results in frameshit (97.9%); premature stop codon in exon 135	normal (100%)	no swim defects	reduced (92%)	normal by WM; normal by TEM	reduced (61%)	significantly shorter (96%)	viable
	alt 2: full intron inclusion (2649 bp) (2.1%); + several stop codons							

Note: Percentages in splicing analysis refer to usage of donor/acceptor sites near the mutation; WM = wholemount preparations; Values in parentheses represent percentage change in mutant compared to their wt siblings.

The most consistent correlation identified in this study was the relationship between nebulin protein expression and the various phenotypes. The two mutants with nonsense mutations, plus *neb^hu28^* (which produces only one alternative transcript that promotes frameshift and introduction of PTCs), produced essentially no transcript or protein. They had fully penetrant phenotypes that were the most severe and that showed the least variability. They reveal the fundamental abnormalities in muscle structure and function associated with loss of nebulin in zebrafish, including shortened thin filament length that correlates with reduced muscle force production, a well establish relationship [37]. They emphasize that the absence of nebulin not only alters this fundamental property, but also promotes widespread muscle disorganization. These results are similar to what has been observed in other pre-clinical knockout models of NEB-related NM [20, 23, 42]. One notable difference, though, is that mouse knockouts are perinatal lethal, with the early postnatal mice exhibiting little to no movement. In contrast, the nonsense mutant zebrafish have their first motor deficits at 6 dpf, and survival until 12 dpf. This suggests that nebulin is not required for embryonic and early larval muscle function, and that larval zebrafish are able to produce forceful enough muscle contractions to sustain some movement even after phenotype onset.

Two additional interesting observations from the mutant lines harboring nonsense mutations were noted. One is that, even though there was very little transcript measured and no detected protein by gel analysis, there were punctae of protein expression observed by immunostaining. This might represent aggregated, truncated versions of nebulin. Second is the fact that proteosome components and cell stress pathways are upregulated by RNAseq analysis. This fits with the presence of aggregated protein(s) and the observed nebulin punctae in immunostained preparations. One hypothesis that emerges from this is that proteotoxic stress contributes to the phenotype of *neb* mutants with nonsense mutations, and that modulating this may promote improvement. We did perform a pilot study of treatment of *neb* nonsense mutants with MG132, a proteosome inhibitor, but this experiment did not result in any difference in *neb* mutant swim performance compared to untreated zebrafish (data not shown).

The three new splice mutants all present important observations. One finding seen across all three mutants is that the splice mutations result in the production of multiple abnormal alternative transcripts. In each, there is a predominant transcript, and then at least one other transcript seen at a lower frequency. In general, the splice mutants show more individual phenotypic variability, a finding that can be explained in large part by the ratio of different aberrant splice isoforms in each individual zebrafish. Another shared observation is the increase in expression of spliceosome pathway components. This makes sense in that these mutations involve RNA processing; however, we are not aware of whether such changes are unique to splice site changes in *neb*, or are more generalizable to splice mutants.

In *neb^21^*, two of three transcripts (accounting for 95% of transcripts) introduce premature stop codons. This is reflected by significant reduction in protein levels, but interestingly not with changes in transcript levels. A plausible explanation for this difference is that these transcripts are expected to be subjected to nonsense-mediated decay. In other ways, this mutant shares features with the nonsense mutants, showing reduced birefringence, shorter thin filament length, and presence of protein aggregates. Forces were significantly different at the lowest and highest frequencies, but not in the intermediate ones, although they trended to be lower. Surprisingly, despite these changes, there was no significant change in swim behavior. This may reflect sample size, variability within the mutant subgroup, and/or the insensitivity of swim behavior as a measure of reduced force generation.

*neb^34^* has several interesting features. 60% of transcripts have an in-frame deletion of 18 base pairs, and 40% have premature stop codons. Protein levels are decreased by a little more than 50%, which may reflect both nonsense-mediated decay and instability of the truncated protein produced from the in-frame transcript. While it may seem unlikely that such a small deletion would cause nebulin protein instability, this has been described with other nebulin mutants, and particularly the recurrent exon 55 deletion, which removes 35 amino acids [42]. An emerging hypothesis is that this is caused by the partial interruption of the super repeat sequence, causing the nebulin protein to not properly (or stably) bind with the actin in the thin filament. Additional mutants that create small in-frame deletions will be necessary to test this concept. In addition, even though there is approximately 50% of mutant protein generated, the reductions in thin filament length and force generation are similar to those seen with the nonsense mutations. This suggests that the mutant protein is functionally abnormal, as well. Of note, zebrafish heterozygous for nonsense mutations have no phenotypic abnormalities, and so the changes in *neb^34^* are not solely due to changes in protein levels.

The final splice mutation, *neb^30^*, is located at the extreme C-terminus, and results in stable levels of a nebulin protein with 45 missense and 31 missing amino acids. There are essentially no obvious abnormalities in this mutant, including normal survival, swim behavior, birefringence, and muscle force production. Mean force values are lower than wild types, but do not reach statistical significance. The only significant alteration was in thin filament length, where there was a small decrease in both heterozygous and homozygous mutant zebrafish. The observation of change in the heterozygous zebrafish may indicate that C-terminus truncated nebulin exerts a dominant negative effect.

The modest impact of the *neb^30^* mutation aligns with findings from studies in mice with a deletion in nebulin’s C-terminus, where no structural defects were observed [27], but is in contrast with what has been reported in human patients with NEB mutations located in that region, who display a severe NM phenotype [8]. The C-terminus end of nebulin includes the last several M-domains, the serine-rich and SH3-domains, and extends into the Z-disk [43, 44], where it interacts with α-actinin [45] and myopalladin [46, 47], among other Z-disk constituent proteins. One possible explanation is that in *neb^30^* zebrafish the mutation is located in the Serine-rich domain, not in the M repeats located before this domain, as is the case with human patients with severe NM [8]. This may allow the *neb^30^* mutant nebulin to integrate into the Z-disk. The subtle functional defects in these zebrafish, such as the lower (non-significant) force production and reduced thin filament length, are similar to results described for mice with mutations in the SH3 domain [27]. The SH3 and the Serine-rich domains are involved in regulating myofibrillar assembly and disassembly [43], functions consistent with the modest changes observed in both our zebrafish *neb^30^* and the SH3 mutant mice.

While the observations seen across our allele series are largely consistent with findings in mouse models and in patients, there were some differences. The force kinetics studies measure the interplay between Ca^2+^ homeostasis, excitation-contraction coupling, and actin-myosin cross-bridge cycling. In the mutants described here, we did not observe differences in any of the parameters examining these features. Other studies (in mice and cells) have shown that nebulin can regulate cross-bridge cycling [36, 48]. The absence of change in our study could be due to differences in nebulin function between species, or could be due to compensatory mechanisms in either calcium homeostasis or excitation-contraction coupling [49–51].

RNAseq analysis revealed additional changes in *neb* processing outside of the mutation areas. For instance, skipping of exons 34–36 was observed in *neb^11^, neb^15^, neb^30^* and *neb^hu28^* but not in wild-type controls. The consequence of this change is predicted to be skipping of one super-repeat (SR5). Physiological skipping of a full super repeat has been observed previously in mouse skeletal muscle [52], and was hypothesized to allow for slight adjustments of nebulin length, and also providing functional benefits, such as fine-tuning of isometric force production [52]. Why this change only occurs in mutant (and not wild-type) zebrafish is unclear. We also observed exon 107 skipping in both wild types and mutants. Human and mouse orthologs of nebulin exon 107 are developmentally regulated, and their inclusion increased with age [53]. Low inclusion of exon 107 likely reflects the young age of zebrafish used for analysis. Finally, we saw variable inclusion of exons in nebulin’s C-terminus region (exons 121–129). These encode simple repeats & Z-repeats, which are known to be differentially expressed based on muscle type and have been proposed to play a role in Z-disk width regulation [44, 52].

A long-term goal is to identify and develop therapies for NEB-related nemaline myopathy. In this study we have advanced this goal by establishing quantifiable outcome measures (e.g. thin filament length, force production, swim behavior, and survival). This will aid future testing of potential therapeutics. In terms of identifying potential treatment strategies, while no direct targets emerged from this work, our in-depth comparative transcriptome analysis may provide some clues for future investigations. The observation of changes in cellular and proteotoxic stress suggests that examining modulators of these pathways may be worth considering in future studies. In addition, we elucidated individual transcripts that were significantly dysregulated. For example, *actc1* levels were significantly decreased in all mutants. This is noteworthy because previous studies in mice have shown that increased expression of *Actc1* can modify the phenotype of a different model of nemaline myopathy (ACTA1-related NM) [54]. One transcript, *mustnb1*, was upregulated across all models. As mustn1b is required for muscle development [39], this may reflect immaturity of skeletal muscle with nebulin mutation, though its significance is worth additional consideration.

In all, we provide a comprehensive examination of the first allele series of nebulin mutations. Results presented in this study validate many previous observations about nebulin function and NEB mutations. It also identifies new areas for study, lays important groundwork and establishes key parameters for studies of disease pathomechanisms and for NEB-related NM therapy development.

## Materials & methods

### Zebrafish husbandry and strains

Zebrafish were maintained in accordance to Canadian Council on Animal Care Guidelines (ISBN: 978-0-919 087-84-2) and all protocols used in this study were approved by the Animal Care Committee at the Peter Gilgan Centre for Research and Learning (PGCRL) at The Hospital for Sick Children (Protocol #: 65697). The following ENU-generated *neb* mutants were obtained from Zebrafish International Resource Center (ZIRC) (Eugene, OR, USA): *neb*^*15*^ (sa15371), *neb*^*11*^ (sa11973), *neb*^*21*^ (sa21475), *neb*^*34*^ (sa34628), and *neb*^*30*^ (sa30646). The *neb*^*hu28*^ (hu2849), generated during Zebrafish Mutation Project, was obtained from Wellcome Sanger Institute (Cambridge, UK).

All the *neb* lines were maintained as heterozygotes and homozygous progeny was obtained through incrosses.

### Genotyping

Genotyping for all experiments except muscle mechanics, was done using genomic DNA extracted from tail clips or heads and specially designed Taqman PCR probes and protocols (Thermo Fischer Scientific). All embryos that were not raised to adulthood were sacrificed in accordance with our established protocols. For all the experiments described in this paper, the zebrafish were genotyped either before or after the experiment.

High throughput zebrafish genotyping for muscle mechanics experiments was performed by fin clipping [55]. Three days post-fertilization (dpf) zebrafish larvae were anesthetized in 1.5 mM Tricaine in E3 embryo media (5 mM NaCl, 0.17 mM KCl, 0.33 mM CaCl_2_, 0.33 mM MgSO_4_, pH 7.2). Under a stereo microscope, tail fins of anesthetized fish were excised using a micro scalpel and then transferred to PCR tubes containing 15 μl 50 mM NaOH using a P20 pipettor. The sample was heated at 98°C for 2 min, cooled down and neutralized with 6 μl of 500 mM Tris-HCl, pH 8.0, followed by brief centrifugation to pellet cellular debris. Extracted DNA was subjected to TaqMan SNP genotyping assays according to the manufacturer’s instructions provided by Applied Biosystems. TaqMan reactions were performed in 96-well plates in a mixture containing approximately 1 ng of zebrafish genomic DNA, 5 μl TaqMan genotyping master mix and 0.25 μl of TaqMan SNP Genotyping Assay (Thermo Fisher Scientific, Waltham, MA, USA) in a volume of 10 μl. The PCR was performed using a Light Cycler 480 system (Roche Diagnostics, Switzerland). The PCR was as follows: initial denaturing at 95°C for 10 min; 40 cycles of 95°C for 15 s and 60°C for 1 min. Each 96-well plate contained 40 samples of an unknown genotype with two replicates, 4 reactions with known genotype (heterozygous) as positive control and 4 reaction mixtures containing the reagents, but no DNA (negative control). The crossing point-PCR-Cycle (Cp) of the target gene was calculated by Light Cycler 480 software release 1.5.0 SP4 (Applied Biosystems) and genotypes of strains were determined by Basic Relative Analysis.

### Swim assay

Swimming behaviour of 7dpf zebrafish larvae was monitored using the Zebrabox platform (Viewpoint Behaviour Technology, Lyon, France), as previously described [56, 57]. Briefly, zebrafish larvae were placed into 96-well plates in 150 µl system water and treated with the optovin analogue 6b8 (ChemBridge, #5707191) at 10 µM final concentration to promote motor behaviour. The Zebrabox protocol used consisted of 30 s light exposure, 1 min dark, 30 s light, 1 min dark, and 30 s light. Time spent moving, total distance travelled, and average velocity during the light exposure periods were measured.

### Birefringence

Zebrafish were anaesthetized with 0.04% tricaine and mounted in 3% methylcellulose on glass slides. Muscle integrity of zebrafish larvae was assessed at 3–7 dpf using an Olympus SZX7 stereoscope equipped with two polarizing filters. Images were acquired with a Firefly camera (Belmont, MA, USA). Quantification of birefringence intensity was done by measuring the brightness of pixels (grey scale value) along dorsal and ventral plot profiles of zebrafish embryos at 6 dpf using Image J (ImageJ, U. S. National Institutes of Health, Bethesda, Maryland, USA, (https://imagej.nih.gov/ij/).

### Survival analysis

Progeny from incrosses of *neb* heterozygous zebrafish lines were genotyped at 5 dpf (tail fin clips) and 20 fish from each genotype (wild type, mutant) were placed in separate tanks and added to the fish facility nursery for feeding. Larvae in each tank were counted daily and dead fish were removed from tanks. Survival curves were generated using Microsoft Excel.

### Microscopic preparations and immunofluorescence staining

Wholemount and myofiber microscopic preparations of 6–7 dpf zebrafish embryos were prepared as previously described [57, 58]. Incubation with primary antibodies was done overnight, at 4°C, and incubation with secondary antibodies and Rhodamine-Phalloidin was done for 2–3 h, at room temperature. The following dilutions were used for wholemount preparations: mouse anti-alpha-Actinin (1:100; Sigma, A7811), Alexa Fluor 488 goat anti-mouse (1:250; ThermoFisher Scientific), and Rhodamine-Phalloidin (1:200; Molecular Probes). The following dilutions were used for myofiber preparations: rabbit anti-N-Nebulin (1:300; Myomedix), mouse anti-alpha-Actinin (1:100; Sigma, A7811), Alexa Fluor 488 goat anti-rabbit (1:1000; ThermoFisher Scientific), and Alexa Fluor 555 goat anti-mouse (1:1000; ThermoFisher Scientific). ProLong Gold Antifade Mountant with DAPI (ThermoFisher Scientific) was used as mounting medium.

### Microscopy and image analysis

Preparations were visualized with a Nikon A1R laser confocal microscope, using a 63x oil-immersion lens. Images were acquired with NIS Elements software (Nikon Instruments Inc., Melville, NY, USA), imported in Volocity (Quorum Technologies Inc., Puslinch, ON, Canada) for processing, and edited using Adobe Photoshop (Adobe Inc., San Jose, CA, USA). Images were only adjusted for brightness and contrast. Diagrams have been created with BioRender.com or Adobe Illustrator.

### Transmission electron microscopy

Zebrafish larvae at 6 dpf were fixed in 2% paraformaldehyde and 2.5% glutaraldehyde in 0.1 M sodium cacodylate buffer overnight, at 4°C, and sent to Advanced Bioimaging Center (PGCRL, The Hospital for Sick Children, Toronto) for processing. Samples were rinsed in 0.1 M sodium cacodylate buffer, post-fixed in 1% osmium tetroxide in buffer for 90 min, dehydrated in a graded ethanol series (50%, 70%, 90% and 100%) followed by two propylene oxide changes for 30 min, and embedded in Quetol-Spurr resin. Blocks were cured overnight in the oven at 60°C. Sections 80 nm thick were cut on a Leica EM UC7 ultramicrotome and stained with uranyl acetate and lead citrate. Sections were imaged with a JEOL JEM 1200EX TEM (JEOL, Massachusetts, USA) (Electron Microscopy Facility at the Laboratory of Pathology, The Hospital for Sick Children, Toronto). Images were obtained using AmtV542 software, and were manipulated only for brightness and contrast using Adobe Photoshop (Adobe Inc., San Jose, CA, USA).

### Muscle mechanics

Mechanical experiments were done on zebrafish larva at 4 dpf using modified version of previously published protocols [36, 48]. Larvae were selected for the study at random from the wild-type or homozygous mutant pools. Experiments were conducted by alternately selecting wild-type or mutant fish to minimize age variation between groups due to the rapid development of larva (~over 6 h period). Larva were anesthetized in 0.02% tricaine and transferred to a small experimental chamber containing Tyrodes solution of the following composition (in gram per 1000 ml): 7.977 NaCl, 0.373 KCl, 0.102 MgCl_2_.6H_2_O, 0.265 CaCl_2_.2H_2_O, 1.000 NaHCO_3_, 0.048 NaH_2_PO_4_ [20]. The larva was mounted horizontally in the 400 µl test chamber of the 801C small intact muscle apparatus by Aurora Scientific, between an 50 mN isometric force transducer (Aurora Scientific, model 400A) and a lever arm of a length controller (Aurora Scientific, 322C). Monofilament nylon suture (size 10-0) was used to fix the larva in place, with ties placed anterior to the swim bladder and near the posterior end of the larva. The experimental chamber was attached to the stage of an inverted microscope (Nikon DIAPHOT 300). Parallel platinum electrodes inside the test chamber were connected to a biphasic muscle stimulator (Aurora Scientific, model 701B). Muscle striations were viewed through the transparent chamber bottom. A video system (Aurora Scientific, 901D) monitored striation spacing and reported sarcomere length, which was adjusted by changing the length of the larva preparation. The current progressively increased until twitch force reached the plateau and did not increase further. Further experiments were conducted at this current. Then larvae were stimulated at slack sarcomere length at 1-ms pulse trains of 150-ms duration at 1, 5, 10, 20, 40, 60, 80, 100, 150, 200 Hz. Larvae were unstimulated for 5 min after each train of stimuli. The maximum force attained in twitches or tetanus at each frequency was taken to represent force attained at that frequency in milli-Newtons. Then larvae were progressively stretched to achieve optimum sarcomere length, the point at which force production after stimulation for 1 ms at 60 Hz no longer increased with length. The larvae were then allowed to rest for 5 min. After the rest period, larvae were stimulated at the same train of frequencies was used at slack length. After mechanical testing, the preparation remained at optimal length and the width of the trunk was measured at three different points to get an average of the width. Then the preparation was rotated 90°C, and its depth at the same anatomical positions was measured. The maximum cross-sectional area (CSA) of the preparation was calculated by modeling the cross section of the zebrafish preparation as an ellipse.

Single twitch force, tetanus contraction at 200Hz, force-frequency relationship, time-to-peak- twitch force (t10-t100) as activation kinetics and time required for peak twitch force to decay (t100-t10) as relaxation kinetics were measured in wild-type and mutant groups at both slack and optimum sarcomere length and were compared using two-way Anova statistical analysis.

### Thin filament length measurement

The protocol for thin filament length measurement was a modified version of the protocol described in [32]. In brief, larvae at 3 dpf from the wild type, mutant and heterozygous pools were anesthetized in 1.5 mM Tricaine in 1X E3 embryo media and stretched around 15% from slack length, fixed in 10% formalin for at least 2 h, washed four times with PBS for 15 min each, embedded in Tissue-Tek O.C.T compound, and immediately frozen in 2-methylbutane precooled in liquid nitrogen and stored at −80°C. The O.C.T. embedded specimen was sectioned into 5 μm thick with Microm HM 550 (Thermo Scientific) microtome and placed on glass slides. Fixed tissues were permeabilized again with 0.2% Triton X-100 in PBS for 20 min at room temperature on a light box to bleach out the background fluorescence, washed with 1X PBS four times and then incubated overnight at 4°C with Alexa Fluor 488-conjugated phalloidin (1:1000; A12379; Invitrogen). The sections were then washed three times with PBS for 15 min each and coverslips (Fisher Finest Premium Cover glass, size: 22X50X1) were mounted to glass slides using Aqua Poly/Mount (Polysciences Inc.). Images were captured using a Deltavision RT system (Applied Precision) with an inverted microscope (IX70; Olympus), using a 100X objective, and a charge-coupled device camera (CoolSNAP HQ; Photometrics) using SoftWoRx 3.5.1 software (Applied Precision). The images were then deconvolved using SoftWoRx. Deconvolved images were reopened in ImageJ (http://rsb.info.nih.gov/ij), then the 1D plot profile was calculated along the myofibril direction. The plot profile was analyzed using Fityk0.9.8 (http://fityk.nieto.pl). A custom “rectangle + 2 half Gaussian” function was used for analyzing phalloidin-stained images that consisted of a rectangle that was flanked by two half Gaussian curves. To account for actin overlapping in the Z-disk which creates a small bump in the center of the rectangle, we developed a special script designed for Fityk that de-activates the center points within the rectangle fit. This improved the subsequent fit for the “rectangle + 2 half Gaussian” function. Thin filament length was calculated as half the width of the rectangle plus half the width of the Gaussian fit at half maximum height. Sarcomere length (SL) was calculated from the distance between the centers of two adjacent Gaussian fits. We analyzed a large number of images and measured the thin filament length within the SL range of 1.85–2.2 μm.

### Protein analysis

After mechanical experiments, zebrafish larvae were decapitated and stored in liquid nitrogen and then solubilized in urea buffer [8 M urea, 2 M thiourea, 50 mM tris-HCl, 75 mM dithiothreitol with 3% SDS, and 0.03% bromophenol blue (pH 6.8)] and 50% glycerol with protease inhibitors (0.04 mM E64, 0.16 mM leupeptin, and 0.2 mM phenylmethylsulfonyl fluoride) at 60°C for 10 min [20]. Solubilized samples were centrifuged at 13 000 rpm for 5 min, aliquoted, flash-frozen in liquid nitrogen, and stored at −80°C. Nebulin expression analysis was performed on solubilized samples using a vertical SDS-agarose gel system. 1% gels were run at 15 mA per gel for 3:20 h, then stained using Coomassie brilliant blue, and scanned using a commercial scanner.

### Statistical analysis

All statistical analyses were performed using GraphPad Prism 9.0.

### RNA-seq

Zebrafish larvae at 6 dpf were anaesthetized with 0.04% tricaine and dissected. The heads were used for genotyping and the bodies were used for total RNA extraction using Qiagen RNeasy Mini Kit protocol. Four biological replicates for each homozygous mutant and wild-type sibling *neb* line were submitted to The Centre for Applied Genomics—Next-Generation Sequencing Facility (PGCRL, The Hospital for Sick Children, Toronto) for paired-end sequencing using Illumina NovaSeq 6000 platform. RNA samples were checked on the Agilent Bioanalyzer 2100 RNA Nano chip for RNA integrity (RIN > 7) and quantified using the Qubit RNA HS Assay. RNA library preparation was performed following the NEBNext Ultra II Directional RNA Library Preparation protocol to generate stranded data. In brief, 100 ng of total RNA was used as the input material and enriched for poly-A mRNA using magnetic oligo d(T) beads, fragmented into the 200-300-bases range for 10 min at 94°C and converted to double stranded cDNA. cDNA proceeded to library preparation and dual-index Illumina adapters were added through PCR. Final libraries were validated on the Bioanalyzer 2100 DNA High Sensitivity chip (Agilent Technologies) to check for size and absence of primer dimers, and quantified by qPCR using Kapa Library Quantification Illumina/ABI Prism Kit protocol (KAPA Biosystems). Validated libraries were pooled in equimolar quantities and sequenced on the Illumina Novaseq platform (1 lane Novaseq S4 flowcell) following Illumina’s recommended protocol to generate paired-end reads of 150-bases in length.

#### Analysis design

Reference-based count-based differential gene expression with STAR+HTseq+DESeq2/edgeR was used for sample comparison.

#### RNA-Seq library and reference genome information

Type of library: stranded, paired end. Genome reference sequence and annotations: GRCz11/Ensembl release 106 (http://useast.ensembl.org/Danio_rerio/Info/Index) downloaded on June 6, 2022.

#### Read pre-processing, alignment and obtaining gene counts

The sequencing data is in FASTQ format. The quality of the data is assessed using FastQC v.0.11.5 (http://www.bioinformatics.babraham.ac.uk/projects/fastqc/).

Adaptors were trimmed using Trim Galore (http://www.bioinformatics.babraham.ac.uk/projects/trim_galore/) v. 0.5.0. Trim Galore is running Cutadapt (https://cutadapt.readthedocs.org/en/stable/) v. 1.10. The raw trimmed reads were aligned to the reference genome using the STAR aligner, v.2.6.0c. (https://github.com/alexdobin/STAR, https://academic.oup.com/bioinformatics/article/29/1/15/272537). The filtered STAR alignments were processed to extract raw read counts for genes using htseq-count v.0.6.1p2(HTSeq, http://www-huber.embl.de/users/anders/HTSeq/doc/overview.html). Assigning reads to genes by htseq-count was done in the mode “intersection_nonempty” and only uniquely mapping reads were counted.

#### Pre-processing, alignment and gene counts QC

MultiQC (https://multiqc.info/) v. 1.9 was used to generate a consolidated report from FastQC screening of both untrimmed and trimmed reads, and from RSeQC, FastQ Screen, STAR and htseq-count results.

#### Exploratory analysis and differential gene expression (DGE) analysis with DESeq2

Exploratory analysis was performed using R v.3.6.1. Differential gene expression analysis was performed with DESeq2 (https://bioconductor.org/packages/release/bioc/html/DESeq2.html and http://master.bioconductor.org/packages/release/workflows/vignettes/rnaseqGene/inst/doc/rnaseqGene.html) v. 1.26.0 s, using the same R version.

#### Pathway analysis

The web-based enrichment tools from Metascape (https://metascape.org/) were used to identify the enriched gene-sets and report the results from the multiple comparisons of all the different nebulin alleles (meta-analysis). The instructions in https://metascape.org/gp/index.html#/menu/manual_meta were followed with the following changes: Excel sheets with the differentially expressed genes per each comparison, split by up-regulated (logFoldChange > 0) and down-regulated (logFoldChange < 0) genes, were used separately as input for the program. The threshold to select the genes to include in each comparison was set as the minimum FDR (Benjamini-Hochberg p value correction for multiple comparisons) from the differential expression analysis which gave a number of differentially expressed genes between 100 and 3000 for each up or down list. For neb30646_MT vs neb30646_wild type there was no such FDR threshold, so logFC > = 1 was selected instead. The parameters for Metascape enrichment analysis, for both the shared sets (sets common to all the comparisons in the study) and the selective sets (sets unique to each comparison in the study), were: Min overlap = 3, *P* value cutoff = 0.05, Min enrichment = 1.5. The analysis for each list was repeated twice: once for the shared GO clusters, and a second for only the selective (list-specific) GO clusters. Metascape first identified all statistically enriched terms; cumulative hypergeometric p-values and enrichment factors were calculated and used for filtering. The remaining significant terms were then hierarchically clustered into a tree based on Kappa-statistical similarities among their gene memberships. Then 0.3 kappa score was applied as the threshold to cast the tree into term clusters. Metascape then selected the term with the best p-value within each cluster as its representative term and displayed them in a dendrogram. The heatmap cells were coloured by their p-values; white cells indicate the lack of enrichment for that term in the corresponding gene list.

#### Goseq procedure

We used goseq as an enrichment tool to account for the extreme in gene length, which may affect the probability of a gene been classified as differentially expressed. The same lists used for Metascape were also used for goseq, but the underlying collection used for the gene-sets is different and contains only GO terms, KEGG, Biocarta and Reactome pathways. The enrichment results were visualized in cytoscape (v. 3.8.1) and analyzed in Microsoft Excel. Gene sets (downloaded on 20220722 from http://rest.kegg.jp/  http://www.reactome.org/download/current/ReactomePathways.gmt.zip  http://software.broadinstitute.org/gsea/msigdb/download_file.jsp?filePath=/resources/msigdb/7.0/c2.cp.biocarta.v7.2.entrez.gmt) with zebrafish identifiers were obtained from the R Bioconductor library org.Dr.eg.db, and KEGG pathways from http://rest.kegg.jp/ (KEGG pathways). Reactome and Biocarta pathways were converted from human to zebrafish identifiers using the resources listed in: https://ftp.ncbi.nih.gov/gene/DATA/gene_orthologs.gz

Conversion of ensembl identifiers (used for RNA-seq alignments) to entrez gene identifiers (used for pathway analysis) was achieved using the mapping on the ensemble and ncbi websites.

Software used: R 4.1.3 (2022-03-10), enrichment map 3.3.3, GSEA 4.0.3, Gene Ontology org.Dr.eg.db.

#### UpSet plot

To visualize quantitative relationships between the DEGs in the nebulin zebrafish mutant data set we created an UpSet plot using UpSetR [59, 60].

#### PSI and splice junction analysis

For calculating inclusion percentages of all exons from nebulin transcripts, inclusion reads (IRs) and exclusion reads (ERs) were counted for each exon based on nebulin isoform ENSDART00000143888.3. IRs are reads overlapping the exon being investigated, normalized by exon length. ERs are reads either upstream or downstream that support exclusions of the read. From these factors, the following equations were used to calculate the PSI index using the ASpli R-package [61].


$$ {\displaystyle \begin{array}{l}{\mathrm{IR}}_{i,n}={\mathrm{IR}}_i/\mathrm{length}\ {\mathrm{exon}}_i+\mathrm{read}\ \mathrm{length}-1\\{}{\mathrm{ER}}_{i,n}={\mathrm{ER}}_i/\mathrm{read}\ \mathrm{length}-1\\{}{\mathrm{PSI}}_i=\left({\mathrm{IR}}_{i,n}/{\mathrm{IR}}_{i,n}+{\mathrm{ER}}_{i,n}\right)\%\end{array}} $$

where *i* is the exon number and *n* is the normalized read counts.

For detection of alternative splicing events around mutations, the most frequent events were considered, and junction reads were used to calculate relative frequencies of events (alternative junction usage vs. intron inclusion vs. normal junction usage).

## Supplementary Material

Fabian_et_al-Supplemental_Figure_1_ddae033

Fabian_et_al-Supplemental_Figure_2_ddae033

Fabian_et_al-Supplemental_Figure_3_ddae033

Fabian_et_al-Supplemental_Figure_4_ddae033

Fabian_et_al-Supplemental_Figure_5_ddae033

Fabian_et_al-Supplemental_Figure_6_ddae033

Fabian_et_al-Supplemental_Figure_7_ddae033

Supplemental_Table_1_ddae033

Supplemental_Table_2_ddae033

Supplemental_Table_3_ddae033

Supplemental_Table_4_ddae033

Supplemental_Table_5_ddae033

Supplemental_Table_6_ddae033

Supplemental_Table_7_ddae033

Supplemental_Table_8_ddae033

Fabian_et_al_Supplemental_Material_accepted_ddae033

## Data Availability

All data relevant to the study are provided in the manuscript. Source images and data are stored at the Hospital for Sick Children and are available upon request.
