# Supplementary figures and images for: Comprehensive phenotypic characterization of an allelic series of zebrafish models of NEB-related nemaline myopathy
Source: Hum Mol Genet. 2024 Mar 17;33(12):1036–54. doi: 10.1093/hmg/ddae033 (PMC11153343; doi:10.1093/hmg/ddae033)

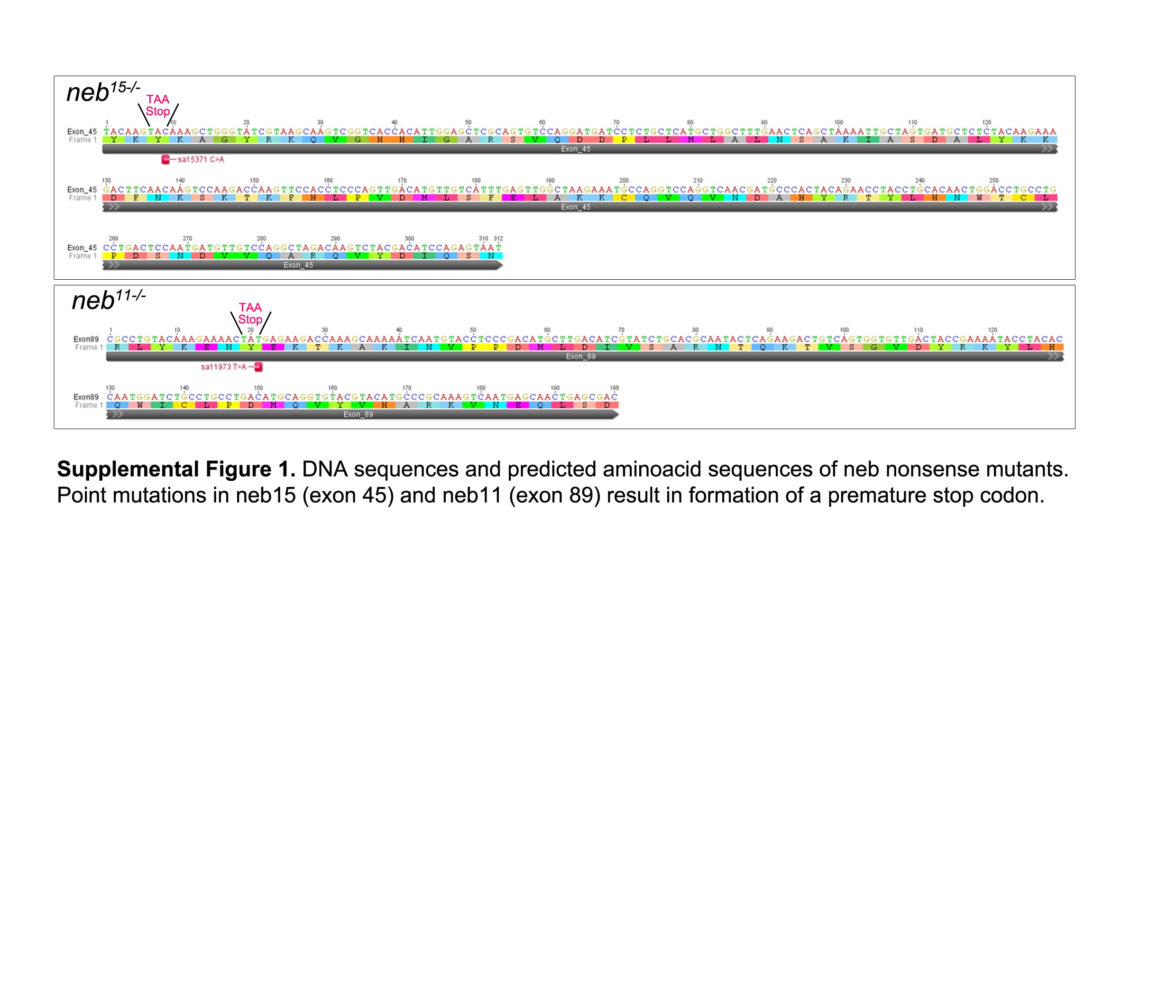

Supplement: Fabian_et_al-Supplemental_Figure_1_ddae033 [file fabian_et_al-supplemental_figure_1_ddae033.jpeg]

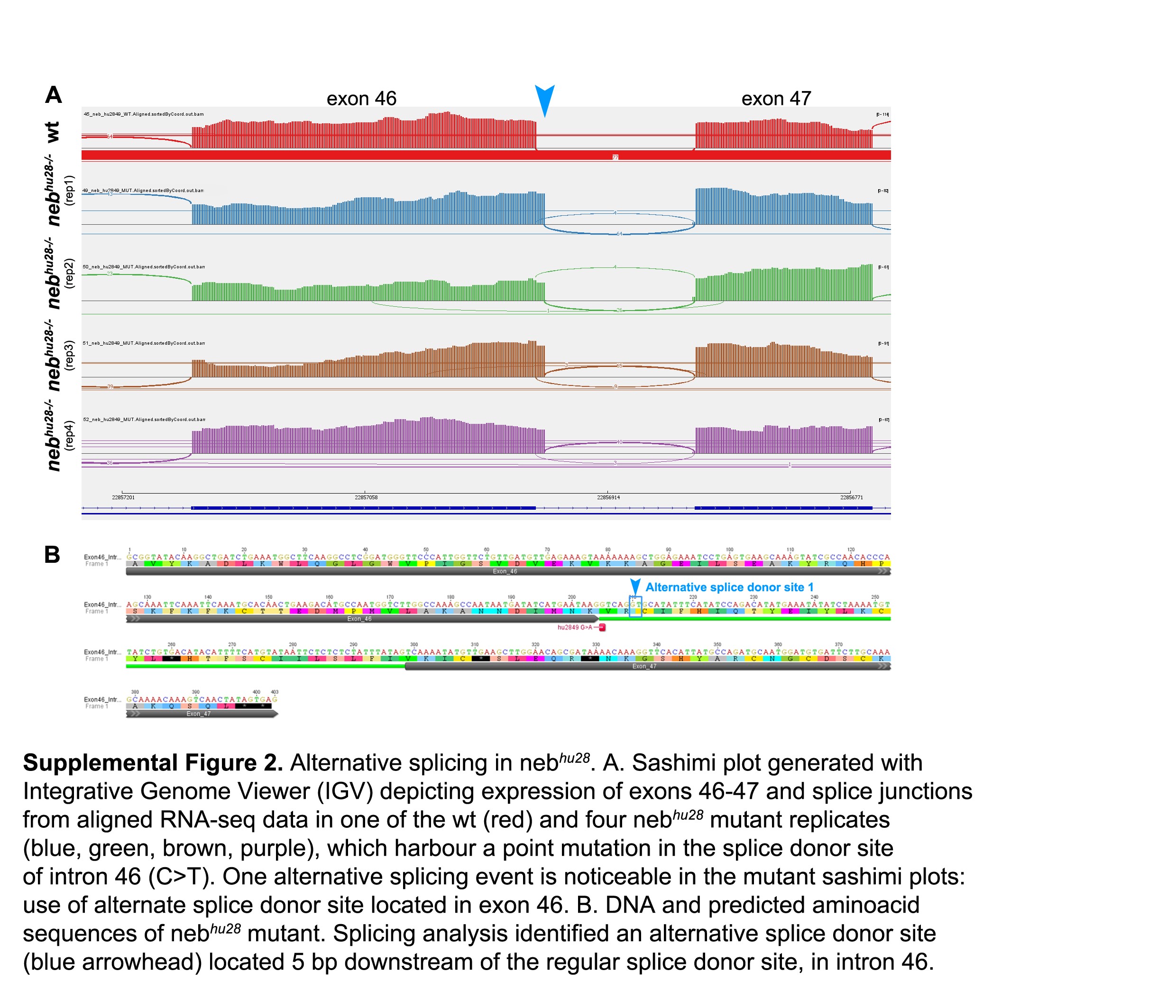

Supplement: Fabian_et_al-Supplemental_Figure_2_ddae033 [file fabian_et_al-supplemental_figure_2_ddae033.jpeg]

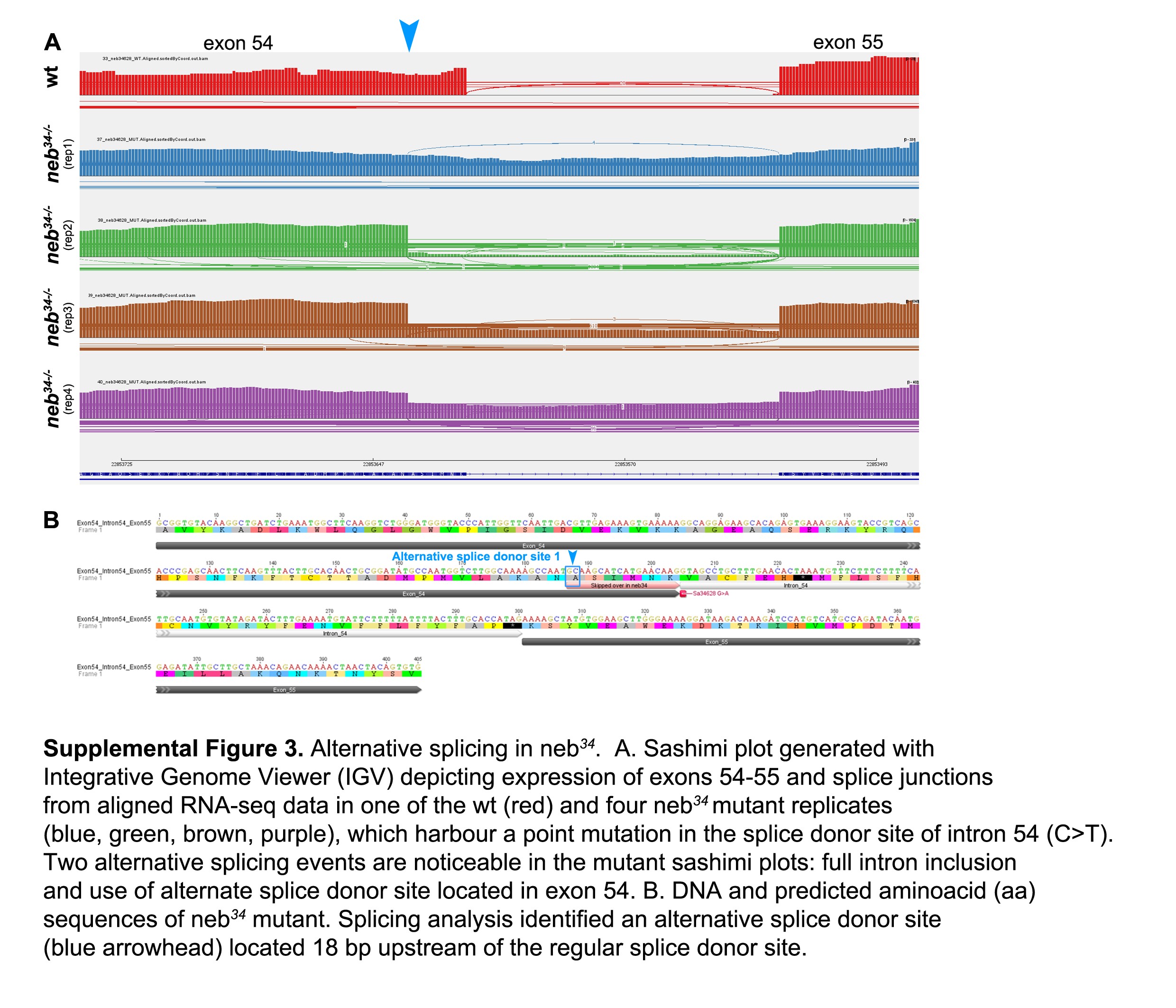

Supplement: Fabian_et_al-Supplemental_Figure_3_ddae033 [file fabian_et_al-supplemental_figure_3_ddae033.jpeg]

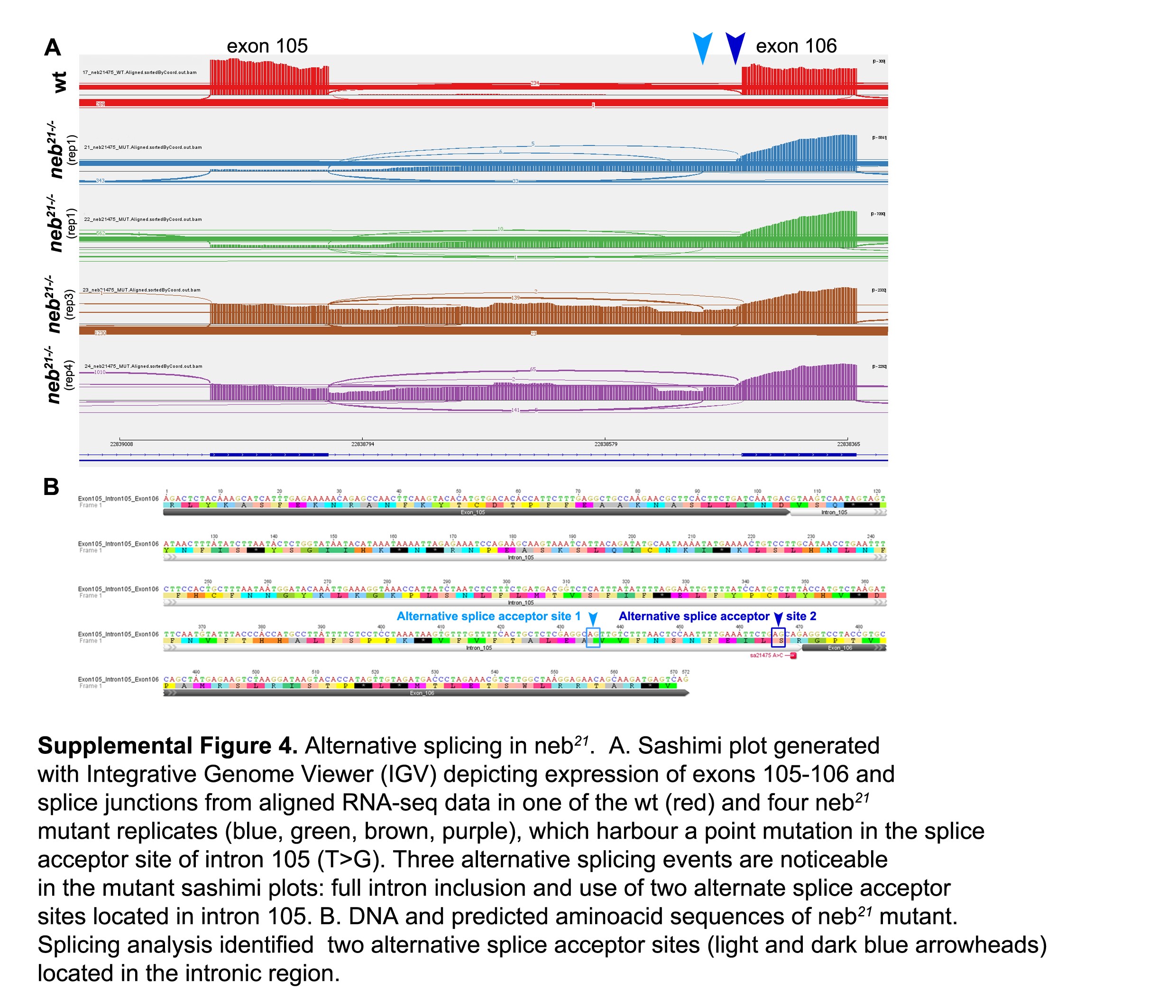

Supplement: Fabian_et_al-Supplemental_Figure_4_ddae033 [file fabian_et_al-supplemental_figure_4_ddae033.jpeg]

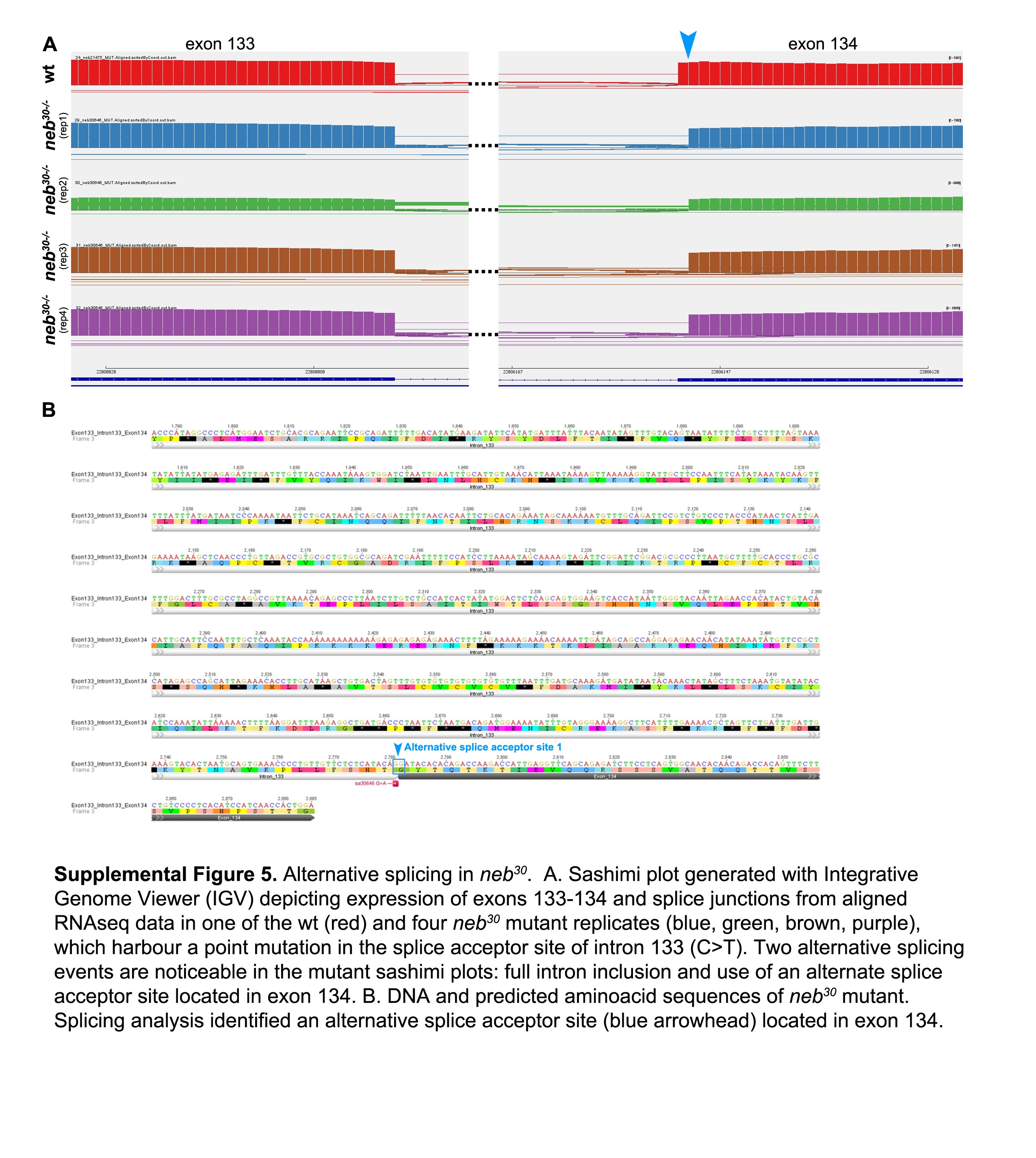

Supplement: Fabian_et_al-Supplemental_Figure_5_ddae033 [file fabian_et_al-supplemental_figure_5_ddae033.jpeg]

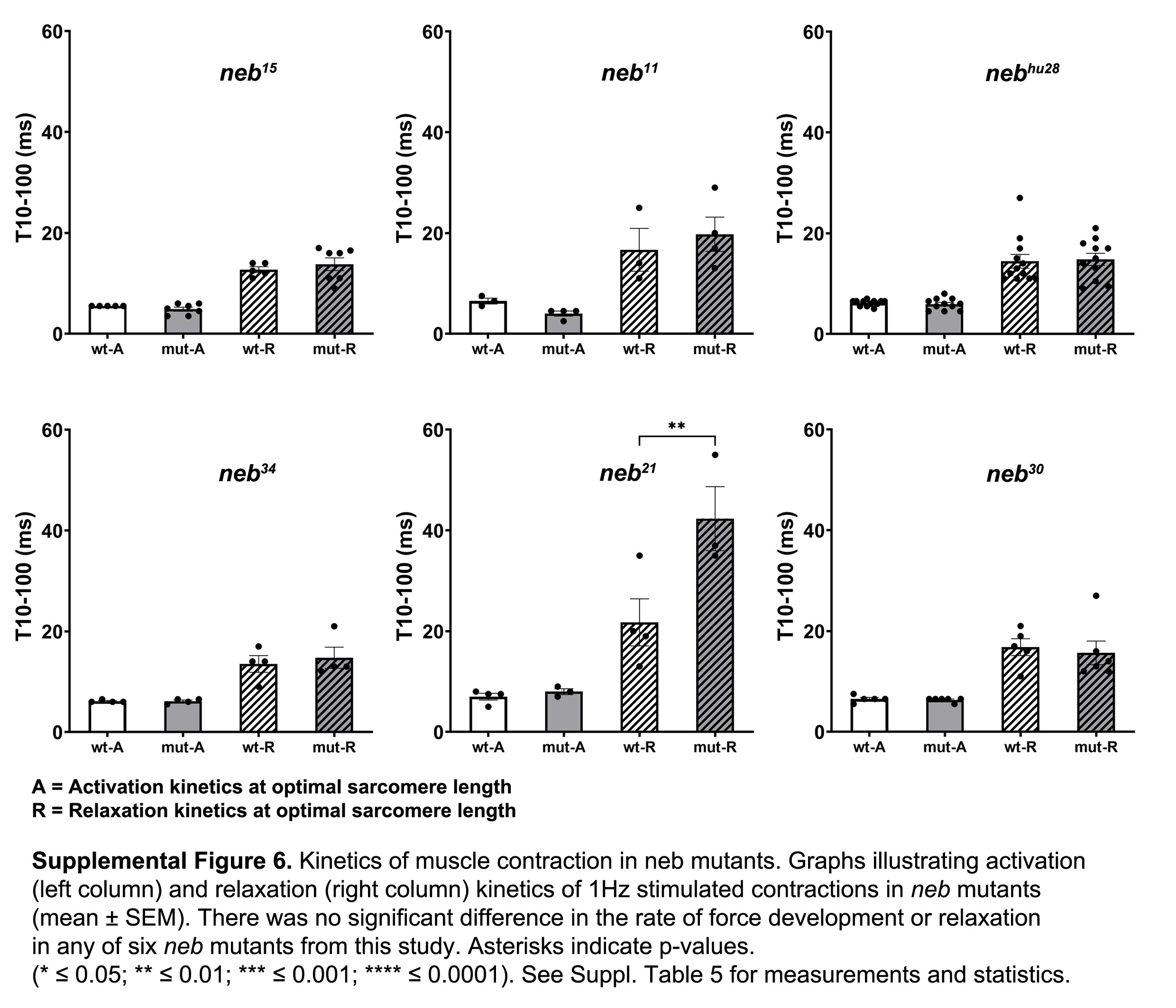

Supplement: Fabian_et_al-Supplemental_Figure_6_ddae033 [file fabian_et_al-supplemental_figure_6_ddae033.jpeg]

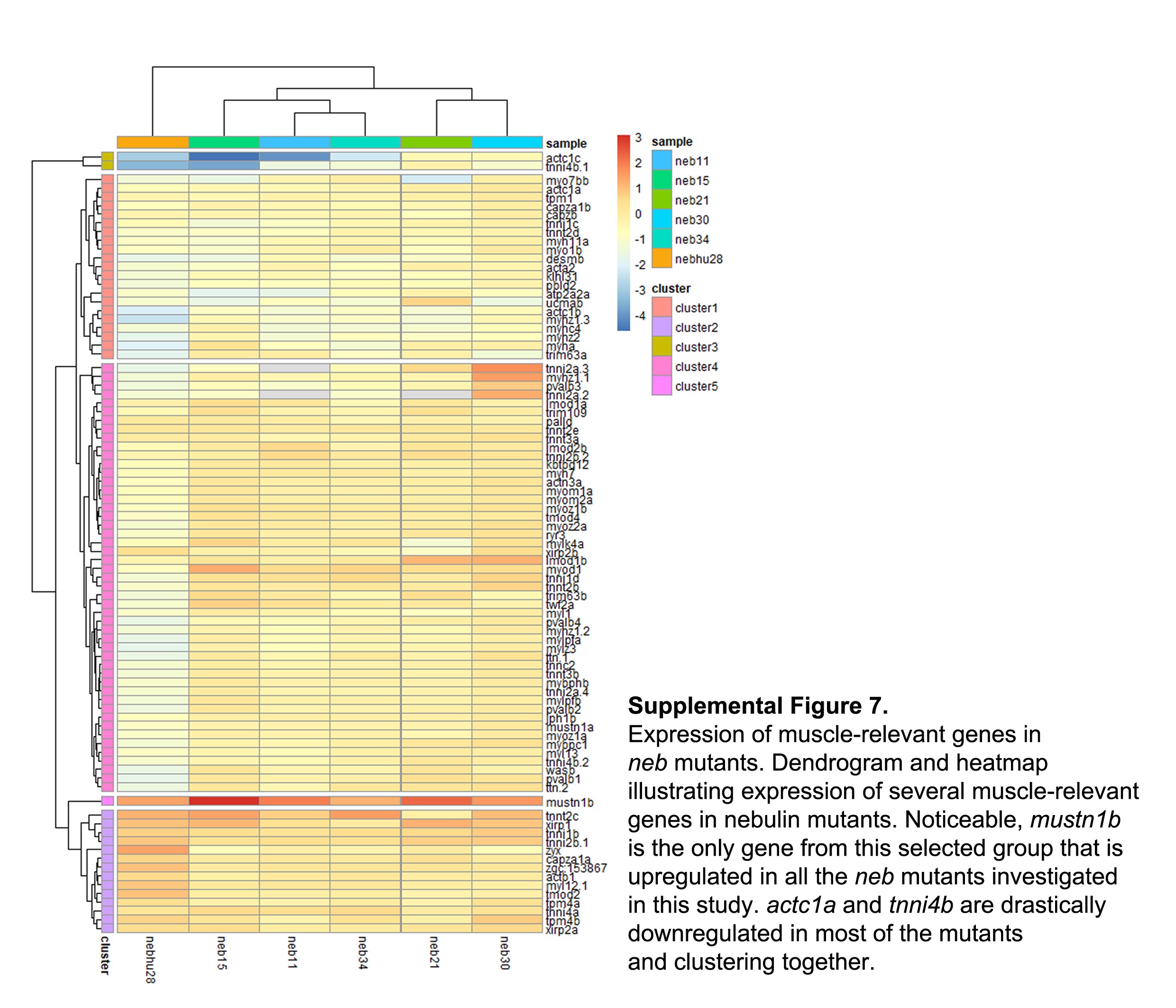

Supplement: Fabian_et_al-Supplemental_Figure_7_ddae033 [file fabian_et_al-supplemental_figure_7_ddae033.jpeg]
