# Supplementary material for: Comprehensive phenotypic characterization of an allelic series of zebrafish models of NEB-related nemaline myopathy
Source: Hum Mol Genet. 2024 Mar 17;33(12):1036–54. doi: 10.1093/hmg/ddae033 (PMC11153343; doi:10.1093/hmg/ddae033)
Supplement: Supplemental_Table_1_ddae033 [file supplemental_table_1_ddae033.pdf]

		average intensity		
		wt	neb^{34}	neb^{30}
myofiber 1	sarcomere 1	98.56	63.04	76.45
	sarcomere 2	93.68	74.53	81.94
	sarcomere 3	92.51	57.96	83.18
	sarcomere 4	102.43	56.73	84.25
	sarcomere 5	95.59	53.5	79.74
	sarcomere 6	82.39	56.92	90.53
	sarcomere 7	93.1	48.27	88.34
	sarcomere 8	94.42	66.82	85.88
myofiber 2	sarcomere 1	99.48	28.43	76.55
	sarcomere 2	101.39	28.81	82.32
	sarcomere 3	100.7	31.13	78.41
	sarcomere 4	100.69	30.44	83.65
	sarcomere 5	102.67	40.1	78.73
	sarcomere 6	102.37	38.77	68.66
	sarcomere 7	99.62	46.18	75.8
	sarcomere 8	97.38	50	80.06
myofiber 3	sarcomere 1	81.45	69.41	79.83
	sarcomere 2	84.21	55.46	76.65
	sarcomere 3	87.75	60.86	81.69
	sarcomere 4	86.28	52.61	74.99
	sarcomere 5	84.55	64.06	81.77
	sarcomere 6	83.18	67.29	79.36
	sarcomere 7	81.97	56.42	89.55
	sarcomere 8	87.36	65.69	88.94
myofiber 4	sarcomere 1	98.25	66.75	95.15
	sarcomere 2	98.25	72.83	89.8
	sarcomere 3	101.19	63.06	90.68
	sarcomere 4	101.97	59.49	98.87
	sarcomere 5	91.08	52.57	83.2
	sarcomere 6	94.13	49.74	96.87
	sarcomere 7	86.66	64.24	94.22
	sarcomere 8	101.59	66.88	87.53
myofiber 5	sarcomere 1	64.91	60.81	63.64
	sarcomere 2	75.93	57.98	57.92
	sarcomere 3	87.11	50.26	64.47
	sarcomere 4	87.89	63.28	68.14
	sarcomere 5	84.3	61.16	71.43
	sarcomere 6	81.06	52.73	67.14
	sarcomere 7	81.36	63.37	68.84
	sarcomere 8	80.22	56.03	69.67
myofiber 6	sarcomere 1	102.59	51.53	102.66
	sarcomere 2	101.59	50.6	102.64
	sarcomere 3	96.81	41.33	102.67
	sarcomere 4	101.62	33.43	102.67
	sarcomere 5	100.74	36.89	102.67
	sarcomere 6	101.16	48.13	102.67
	sarcomere 7	98.55	46.92	102.67
	sarcomere 8	98.7	41.62	102.67
myofiber 7	sarcomere 1	99.89	48.47	102.67
	sarcomere 2	97.5	52.08	102.67
	sarcomere 3	98.96	51.91	102.67
	sarcomere 4	98.67	51.74	102.67
	sarcomere 5	102.54	51.56	102.67
	sarcomere 6	95.51	39.2	102.67
	sarcomere 7	96.45	46.14	102.67
	sarcomere 8	97.93	44.58	102.67
n		56	56	56
Mean		93.55	52.87	86.74
SEM		1.139	1.509	1.736
%			56.50%	92.70%

Supplemental Table 1. Average fluorescence intensity measurements of anti Neb-N antibody staining in myofiber preparations and descriptive statistics
