# Supplementary material for: Comprehensive phenotypic characterization of an allelic series of zebrafish models of NEB-related nemaline myopathy
Source: Hum Mol Genet. 2024 Mar 17;33(12):1036–54. doi: 10.1093/hmg/ddae033 (PMC11153343; doi:10.1093/hmg/ddae033)
Supplement: Supplemental_Table_2_ddae033 [file supplemental_table_2_ddae033.pdf]

		wt		neb^{15}		neb^{11}		neb^{34}		neb^{21}		neb^{30}	
		n (pixels)	average intensity	n (pixels)	average intensity	n (pixels)	average intensity	n (pixels)	average intensity	n (pixels)	average intensity	n (pixels)	average intensity
**measurement 1**	**fish 1**	148	254.40	145	156.28	184	174.32	138	165.56	168	136.39	117	255.00
	**fish 2**	147	254.92	156	97.59	145	113.32	175	210.39	183	81.21	121	251.45
	**fish 3**	135	255.00	146	124.93	122	35.39	160	153.63	147	154.28	117	253.10
	**fish 4**	124	255.00	131	175.58	147	100.64	157	174.27	147	43.17	124	255.00
	**fish 5**	135	255.00	150	159.04	121	120.42	164	186.36	154	58.03	142	255.00
	**fish 6**	169	250.24										
	**fish 7**	140	255.00										
	**fish 8**	158	255.00										
**measurement 2**	**fish 1**	142	254.97	143	194.88	156	233.27	144	201.54	121	187.05	255.00	255.00
	**fish 2**	122	255.00	135	181.41	132	187.08	145	234.83	180	161.42	255.00	255.00
	**fish 3**	111	254.94	132	189.16	99	61.12	137	221.50	136	231.19	254.77	254.77
	**fish 4**	120	254.95	117	234.72	155	44.15	132	206.52	125	85.69	255.00	255.00
	**fish 5**	110	255.00	145	216.88	132	154.34	148	231.09	150	153.58	255.00	255.00
	**fish 6**	153	254.62										
	**fish 7**	123	255.00										
	**fish 8**	151	255.00										
**n**		16		10		10		10		10		10	
**Mean**		254.6		122.4		173		198.6		129.2		254.4	
**SEM**		0.2955		20.6		12.92		8.793		19.07		0.3806	

Supplemental Table 2. Average birefringence intensity measurements (grey scale value) and descriptive statistics.
