# Supplementary material for: Comprehensive phenotypic characterization of an allelic series of zebrafish models of NEB-related nemaline myopathy
Source: Hum Mol Genet. 2024 Mar 17;33(12):1036–54. doi: 10.1093/hmg/ddae033 (PMC11153343; doi:10.1093/hmg/ddae033)
Supplement: Supplemental_Table_3_ddae033 [file supplemental_table_3_ddae033.pdf]

zebrafish line	neb^{15}			neb^{11}			neb^{34}			neb^{21}			neb^{30}		
**recorded values**															
**time spent moving**	**wt**	**het**	**mut**	**wt**	**het**	**mut**	**wt**	**het**	**mut**	**wt**	**het**	**mut**	**wt**	**het**	**mut**
**n**	11	14	12	9	24	21	12	11	23	9	17	8	13	19	16
**mean**	145.50	142.50	92.00	130.33	117.07	93.47	153.70	170.70	131.90	164.10	150.30	129.20	140.20	114.90	137.50
**SEM**	10.77	8.75	4.45	11.29	6.00	5.55	11.39	10.64	7.13	9.18	8.32	12.57	13.16	8.43	9.45
**distance travelled**	**wt**	**het**	**mut**	**wt**	**het**	**mut**	**wt**	**het**	**mut**	**wt**	**het**	**mut**	**wt**	**het**	**mut**
**n**	11	14	12	9	24	21	12	11	23	9	17	8	13	19	16
**mean**	386.60	386.20	167.80	360.50	337.80	230.00	428.60	447.00	304.00	367.90	389.80	300.90	347.10	301.90	353.40
**SEM**	33.34	23.85	9.48	30.11	15.24	13.49	23.78	25.77	16.21	27.36	22.01	39.01	32.59	28.66	23.79
**% change**			43.40			63.80			70.93			81.79			101.82
**average speed**	**wt**	**het**	**mut**	**wt**	**het**	**mut**	**wt**	**het**	**mut**	**wt**	**het**	**mut**	**wt**	**het**	**mut**
**n**	11	14	12	9	24	21	12	11	23	9	17	8	13	19	16
**mean**	2.64	2.75	1.84	2.85	2.98	2.50	2.92	2.66	2.38	2.27	2.65	2.29	2.53	2.64	2.62
**SEM**	0.12	0.14	0.08	0.19	0.14	0.12	0.21	0.12	0.15	0.17	0.14	0.18	0.17	0.22	0.14
**normalized values**															
**time spent moving**	**wt**	**het**	**mut**	**wt**	**het**	**mut**	**wt**	**het**	**mut**	**wt**	**het**	**mut**	**wt**	**het**	**mut**
**n**	11	14	12	9	24	21	12	11	23	9	17	8	13	19	16
**mean**	1.00	0.98	0.63	1.00	0.90	0.72	1.00	1.11	0.86	1.00	0.92	0.79	1.00	0.82	0.98
**SEM**	0.07	0.06	0.03	0.09	0.05	0.04	0.07	0.07	0.05	0.06	0.05	0.08	0.09	0.06	0.07
**distance travelled**	**wt**	**het**	**mut**	**wt**	**het**	**mut**	**wt**	**het**	**mut**	**wt**	**het**	**mut**	**wt**	**het**	**mut**
**n**	11	14	12	9	24	21	12	11	23	9	17	8	13	19	16
**mean**	1.00	1.00	0.43	1.00	0.94	0.64	1.00	1.04	0.71	1.00	1.06	0.82	1.00	0.87	1.02
**SEM**	0.09	0.06	0.02	0.08	0.04	0.04	0.06	0.06	0.04	0.07	0.06	0.11	0.09	0.08	0.07
**average speed**	**wt**	**het**	**mut**	**wt**	**het**	**mut**	**wt**	**het**	**mut**	**wt**	**het**	**mut**	**wt**	**het**	**mut**
**n**	11	14	12	9	24	21	12	11	23	9	17	8	13	19	16
**mean**	1.00	1.04	0.70	1.00	1.05	0.88	1.00	0.91	0.81	1.00	1.17	1.01	1.00	1.05	1.04
**SEM**	0.05	0.05	0.03	0.07	0.05	0.04	0.07	0.04	0.05	0.08	0.06	0.08	0.07	0.09	0.06

Supplemental Table 3. Descriptive statistics for swim assay measurements (recorded and normalized values for time spent moving, distance travelled, average speed)
