# Supplementary material for: Comprehensive phenotypic characterization of an allelic series of zebrafish models of NEB-related nemaline myopathy
Source: Hum Mol Genet. 2024 Mar 17;33(12):1036–54. doi: 10.1093/hmg/ddae033 (PMC11153343; doi:10.1093/hmg/ddae033)
Supplement: Supplemental_Table_4_ddae033 [file supplemental_table_4_ddae033.pdf]

Frequency	1Hz	5 Hz	10 Hz	20 Hz	40 Hz	60 Hz	80 Hz	100 Hz	150 Hz	200 Hz	1Hz	5 Hz	10 Hz	20 Hz	40 Hz	60 Hz	80 Hz	100 Hz	150 Hz	200 Hz
	neb15 wt sib										neb15 mut									
	8.45	8.17	7.98	7.88	8.02	7.58	7.25	7.42	11.96	16.27	5.52	5.25	5.30	5.38	5.44	5.34	7.94	9.94	13.62	15.40
	34.62	33.69	33.79	32.31	33.30	32.67	31.74	30.85	37.29	47.45	1.81	1.84	1.89	1.86	2.84	4.34	6.32	8.10	12.68	13.14
	16.47	16.24	16.70	16.45	17.82	17.48	17.61	17.30	28.55	37.23	1.84	1.81	1.58	1.83	2.63	4.17	6.68	7.70	9.12	10.38
	6.04	6.27	6.41	6.43	6.63	6.40	6.94	10.15	18.33	24.37	1.87	2.01	1.90	2.04	2.07	3.05	5.32	6.86	9.19	10.46
	13.43	12.89	13.15	13.39	13.24	12.81	12.95	15.76	30.95	40.40	2.50	2.42	2.64	2.76	3.49	5.30	8.39	11.30	14.95	16.88
											0.79	1.08	1.15	0.94	1.22	1.65	2.37	3.12	4.60	5.14
**n**	5	5	5	5	5	5	5	5	5	5	7	7	7	7	7	7	7	7	7	7
**Mean**	15.80	15.45	15.61	15.29	15.80	15.39	15.30	16.30	25.42	33.15	2.18	2.21	2.21	2.26	2.67	3.57	5.51	7.00	9.65	10.78
**SEM**	11.29	10.92	10.96	10.35	10.74	10.63	10.20	9.08	10.16	12.61	1.59	1.42	1.47	1.51	1.51	1.68	2.63	3.39	4.44	4.85
**SD**	5.05	4.88	4.90	4.63	4.80	4.75	4.56	4.06	4.54	5.64	0.60	0.54	0.55	0.57	0.57	0.63	0.99	1.28	1.68	1.83
											percentage force at 200 Hz (compared to wt siblings)									33
	neb11 wt sib										neb11 mut									
	31.11	31.01	29.15	27.40	27.58	25.28	24.70	23.68	25.65	26.09	2.98	2.99	2.99	2.93	2.90	2.83	2.62	3.02	5.05	5.94
	24.05	24.04	23.45	23.66	22.81	22.76	23.78	24.63	25.95	26.68	0.96	0.80	0.88	0.79	0.86	0.94	0.84	0.92	1.03	1.57
	32.93	32.74	30.62	31.66	29.72	29.04	27.95	26.77	30.17	30.43	4.81	4.87	4.71	4.73	4.82	5.73	5.70	5.62	6.30	5.94
											5.22	5.21	4.93	4.95	5.01	5.63	5.82	5.95	6.93	6.59
**n**	3	3	3	3	3	3	3	3	3	3	4	4	4	4	4	4	4	4	4	4
**Mean**	29.36	29.26	27.74	27.57	26.70	25.69	25.47	25.03	27.26	27.73	3.49	3.47	3.38	3.35	3.40	3.79	3.75	3.88	4.83	5.01
**SEM**	2.71	2.66	2.19	2.31	2.04	1.83	1.27	0.91	1.46	1.36	1.95	2.03	1.88	1.93	1.94	2.32	2.44	2.37	2.65	2.32
**SD**	4.69	4.61	3.79	4.00	3.54	3.16	2.19	1.58	2.53	2.35	0.97	1.02	0.94	0.97	0.97	1.16	1.22	1.18	1.33	1.16
											percentage force at 200 Hz (compared to wt siblings)									18
	nebhu28 wt sib										nebhu28 mut									
	20.14	18.52	17.76	17.61	16.21	16.15	16.10	14.98	18.61	21.15	2.04	2.12	2.04	1.79	1.72	1.75	1.84	1.97	2.91	3.03
	14.97	14.87	13.86	14.50	14.12	13.54	13.08	14.01	17.00	20.29	0.21	0.20	0.24	0.23	0.19	0.18	0.20	0.20	0.27	0.27
	16.89	15.70	16.30	15.96	15.43	15.21	15.77	16.79	18.44	20.52	0.88	0.82	0.84	0.78	0.81	0.74	0.66	0.75	0.88	1.04
	17.80	17.40	17.08	16.63	16.18	15.85	15.93	15.77	18.40	19.98	2.11	2.20	2.05	2.00	1.96	1.97	1.89	1.94	2.80	3.37
	11.60	11.71	11.48	11.80	10.31	9.00	8.66	7.79	8.33	9.58	0.54	0.63	0.64	0.65	0.91	1.24	1.62	2.07	1.95	2.29
	30.79	31.26	31.31	31.33	29.74	29.39	29.07	31.68	34.67	35.81	1.64	1.68	1.55	1.61	1.56	1.60	2.40	2.67	3.77	3.99
	28.84	27.89	28.38	31.06	25.58	26.38	26.27	26.27	32.97	28.74	1.79	1.70	1.66	1.74	1.70	1.66	1.65	1.82	2.35	3.24
	29.66	30.30	28.89	28.11	26.55	25.65	23.85	23.94	27.26	27.89	0.53	0.53	0.54	0.52	0.54	0.48	0.61	0.72	1.10	1.08
	34.81	34.98	33.75	33.16	32.39	31.72	30.49	29.84	35.67	36.42	5.19	5.12	4.99	5.00	4.97	5.23	5.50	5.76	7.03	7.44
	40.97	43.84	43.77	43.77	42.36	37.46	36.34	38.70	44.69	40.82	4.37	4.54	4.33	4.50	4.67	5.05	4.99	5.18	6.00	6.35
											7.12	6.65	6.46	5.99	5.01	4.90	4.88	4.53	6.03	5.59
											0.34	0.37	0.30	0.39	0.37	0.37	0.38	0.39	0.47	0.50
**n**	10	10	10	10	10	10	10	10	10	10	12	12	12	12	12	12	12	12	12	12
**Mean**	24.65	24.64	24.26	24.39	22.89	22.03	21.56	21.98	25.60	26.12	2.23	2.21	2.14	2.10	2.03	2.10	2.22	2.33	2.96	3.18
**SEM**	9.67	10.51	10.47	10.50	10.10	9.31	8.92	9.64	11.20	9.59	2.20	2.11	2.04	1.97	1.81	1.88	1.88	1.88	2.31	2.34
**SD**	3.06	3.32	3.31	3.32	3.19	2.94	2.82	3.05	3.54	3.03	0.63	0.61	0.59	0.57	0.52	0.54	0.54	0.54	0.67	0.68
											percentage force at 200 Hz (compared to wt siblings)									12
	neb34 wt sib										neb34 mut									
	31.86	33.41	32.98	31.78	28.90	26.24	27.75	30.58	36.92	41.66	2.98	2.99	2.99	2.93	2.90	2.83	2.62	3.02	5.05	5.94
	42.32	39.69	39.27	37.10	35.02	34.56	34.67	35.11	40.83	42.17	13.82	13.97	13.62	13.98	13.85	14.11	14.70	17.00	19.74	19.99
	29.12	28.32	27.24	26.51	25.58	25.21	24.29	24.59	28.68	28.96	10.37	10.53	9.82	9.78	9.17	8.78	8.80	9.44	10.95	12.61
	6.49	10.66	11.92	11.97	11.10	9.15	8.91	8.89	10.59	13.18	8.53	8.52	8.58	8.36	8.32	7.83	8.03	8.80	10.83	11.54
											7.09	7.19	7.02	6.92	6.63	6.60	6.40	7.66	10.07	10.70
**n**	4	4	4	4	4	4	4	4	4	4	5	5	5	5	5	5	5	5	5	5
**Mean**	27.45	28.02	27.85	26.84	25.15	23.79	23.90	24.79	29.25	31.49	8.56	8.64	8.41	8.39	8.18	8.03	8.11	9.18	11.33	12.16
**SEM**	15.09	12.47	11.70	10.82	10.15	10.62	10.89	11.44	13.43	13.65	4.01	4.07	3.89	4.03	3.98	4.08	4.39	5.04	5.30	5.07
**SD**	7.54	6.24	5.85	5.41	5.07	5.31	5.44	5.72	6.72	6.83	1.79	1.82	1.74	1.80	1.78	1.83	1.96	2.25	2.37	2.27
											percentage force at 200 Hz (compared to wt siblings)									39
	neb21 wt sib										neb21 mut									
	41.24	38.47	38.18	37.24	37.59	35.10	35.90	35.72	43.92	46.58	1.95	2.36	2.54	3.20	4.45	5.36	6.78	7.58	8.52	8.28
	6.15	6.04	5.91	6.67	9.58	11.45	13.84	15.04	17.99	17.89	7.20	7.34	8.17	10.67	14.96	18.21	23.53	29.51	31.74	31.19
	35.15	36.22	36.22	37.25	34.96	36.07	47.45	57.59	75.95	75.45	3.77	4.06	5.00	6.31	8.20	10.47	13.45	14.76	16.88	15.76
	23.06	22.81	22.16	21.85	22.33	22.52	24.57	29.44	29.93	31.62	7.96	7.96	7.65	8.24	10.32	13.08	16.76	17.48	20.52	20.25
**n**	4	4	4	4	4	4	4	4	4	4	4	4	4	4	4	4	4	4	4	4
**Mean**	26.40	25.89	25.62	25.75	26.11	26.28	30.44	34.45	41.95	42.88	5.22	5.43	5.84	7.11	9.48	11.78	15.13	17.33	19.42	18.87
**SEM**	15.47	14.93	14.95	14.64	12.88	11.66	14.48	17.69	25.02	24.67	2.84	2.67	2.60	3.15	4.39	5.35	6.97	9.13	9.63	9.58
**SD**	7.74	7.46	7.48	7.32	6.44	5.83	7.24	8.85	12.51	12.34	1.42	1.33	1.30	1.58	2.19	2.68	3.49	4.56	4.82	4.79
											percentage force at 200 Hz (compared to wt siblings)									44
	neb30 wt sib										neb30 mut									
	8.62	8.28	7.24	6.90	6.77	6.30	5.64	5.62	4.94	5.64	19.27	18.95	18.55	17.76	17.49	16.66	16.41	15.73	16.40	16.92
	38.12	38.13	38.30	37.57	34.54	32.42	31.58	31.66	34.53	35.33	14.46	15.06	14.71	14.97	14.89	12.71	12.11	12.19	13.11	13.68
	34.19	34.66	34.96	35.75	33.50	31.73	35.13	37.08	39.06	42.70	7.44	7.10	7.20	7.03	7.03	6.88	6.77	6.71	8.19	9.44
	11.18	10.47	10.25	10.12	9.37	9.17	9.78	10.95	12.68	14.32	10.30	10.53	9.81	10.04	9.44	9.57	9.88	10.51	13.11	14.25
	44.11	42.38	41.09	39.14	37.80	36.99	37.52	40.50	43.39	45.86	31.01	29.76	27.17	24.63	21.61	22.75	23.71	25.61	27.70	25.53
											21.77	23.20	23.00	22.38	21.41	20.64	21.16	21.12	24.63	25.51
**n**	5	5	5	5	5	5	5	5	5	5	6	6	6	6	6	6	6	6	6	6
**Mean**	27.24	26.78	26.37	25.89	24.40	23.32	23.93	25.16	26.92	28.77	17.38	17.43	16.74	16.13	15.31	14.87	15.01	15.31	17.19	17.55
**SEM**	16.25	16.15	16.27	15.96	15.01	14.41	15.03	15.84	17.04	17.84	8.56	8.34	7.67	6.86	6.08	6.25	6.61	7.02	7.49	6.62
**SD**	7.27	7.22	7.28	7.14	6.71	6.44	6.72	7.08	7.62	7.98	3.49	3.40	3.13	2.80	2.48	2.55	2.70	2.87	3.06	2.70
											percentage force at 200 Hz (compared to wt siblings)									61

Supplemental Table 4. Force production measurements at optimal sarcomere length (recorded values and descriptive statistics)
