# Supplementary material for: Comprehensive phenotypic characterization of an allelic series of zebrafish models of NEB-related nemaline myopathy
Source: Hum Mol Genet. 2024 Mar 17;33(12):1036–54. doi: 10.1093/hmg/ddae033 (PMC11153343; doi:10.1093/hmg/ddae033)
Supplement: Supplemental_Table_5_ddae033 [file supplemental_table_5_ddae033.pdf]

		neb^{15}		neb^{11}		neb^{hu28}		neb^{34}		neb^{21}		neb^{30}	
		wt	mut	wt	mut	wt	mut	wt	mut	wt	mut	wt	mut
**Activation Kinetics**	**Slack**												
		5	5	4.5	4.5	5.5	5.5	6	5.5	8	7	4.5	5.5
		5.5	3.5	5.5	5.5	4.5	3.5	6	5.5	8	6	5.5	6.5
		5.5	6	5.5	4.5	5	1.5	6	6	6.5		5.5	5.5
		5.5	3.5		4.5	4.5	6.5	5.5	4.5	6		6.5	6.5
		5.5	6			4.5		6				5.5	7
			7.5			7							
			6.5										
	**n**	5	7	3	4	6	4	5	4	4	2	5	5
	**Mean**	5.4	5.429	5.167	4.75	5.167	4.25	5.9	5.375	7.125	6.5	5.5	6.2
	**SEM**	0.1	0.5714	0.3333	0.25	0.4014	1.109	0.1	0.3146	0.5154	0.5	0.3162	0.3
**Activation Kinetics**	**Optimal**												
		5.5	6	5.5	4.5	6.5	4.5	6	6.5	8	7	5.5	6.5
		5.5	3.5	7.5	2.5	6	7	6.5	6.5	5	9	6.5	6.5
		5.5	5.5	6.5	4.5	6.5	6.5	6	6	7.5	8	6.5	5.5
		5.5	3.5		4.5	5.5	7	6	5.5	7.5		6.5	6.5
		5.5	5			6.5	5.5					7.5	6.5
			4.5			6.5	6						6.5
			6			7	8						
						5.5	4.5						
						6	6						
						6.5	5.5						
						5	4.5						
						6.5							
	**n**	5	7	3	4	12	11	4	4	4	3	5	6
	**Mean**	5.5	4.857	6.5	4	6.167	5.909	6.125	6.125	7	8	6.5	6.333
	**SEM**	0	0.4041	0.5774	0.5	0.1667	0.3491	0.125	0.2394	0.677	0.5774	0.3162	0.1667
**Relaxation Kinetics**	**Slack**												
		6.5	13	9	9	8	13	10	11	10	26	7.5	12
		9	10.5	13	15	10.5	8	9	13	20	24	12	10
		9	11.5	10	10	9	8.5	11	11	13		11	9
		8	12.5		10	8	10	10	12	10		9	12
		11	9.5			8		8				13	12
			8			10							
			6.5										
	**n**	5	7	3	4	6	4	5	4	4	2	5	5
	**Mean**	8.7	10.21	10.67	11	8.917	9.875	9.6	11.75	13.25	25	10.5	11
	**SEM**	0.7348	0.8988	1.202	1.354	0.4549	1.125	0.5099	0.4787	2.358	1	1	0.6325
**Relaxation Kinetics**	**Optimal**												
		11	16	11	13	19	19	14	21	13	35	11	27
		12	16.5	25	17	14	17	17	13	35	37	16	14
		14	16	14	29	27	9	14	13	19	55	17	12
		12.5	17		20	15	21	9	12	20		21	13
		14	11			12	17					19	16
			9			11	18						12
			11			11	13						
						12	14						
						13	10.5						
						11	9.5						
						11	15						
						17							
	**n**	5	7	3	4	12	11	4	4	4	3	5	6
	**Mean**	12.7	13.79	16.67	19.75	14.42	14.82	13.5	14.75	21.75	42.33	16.8	15.67
	**SEM**	0.5831	1.253	4.256	3.4	1.368	1.207	1.658	2.097	4.679	6.36	1.685	2.348

Supplemental Table 5. Activation and relaxation kinetics (measurements and descriptive statistics)
