# Supplementary material for: Comprehensive phenotypic characterization of an allelic series of zebrafish models of NEB-related nemaline myopathy
Source: Hum Mol Genet. 2024 Mar 17;33(12):1036–54. doi: 10.1093/hmg/ddae033 (PMC11153343; doi:10.1093/hmg/ddae033)
Supplement: Supplemental_Table_6_ddae033 [file supplemental_table_6_ddae033.pdf]

zebrafish line	neb^{15}			neb^{11}			neb^{hu28}			neb^{34}			neb^{21}			neb^{30}		
	wt	het	mut	wt	het	mut	wt	het	mut	wt	het	mut	wt	het	mut	wt	het	mut
n	607	620	721	664	473	493	346	528	301	161	136	330	1262	793	625	549	149	117
mean	0.8075	0.8341	0.7588	0.8388	0.8529	0.7645	0.8192	0.8166	0.6928	0.8239	0.8308	0.7213	0.8301	0.8112	0.7816	0.8538	0.8161	0.8235
SEM	0.002098	0.002382	0.002455	0.002033	0.00324	0.00274	0.002791	0.002774	0.005091	0.004105	0.003927	0.003967	0.00121	0.001855	0.002274	0.002222	0.004387	0.006603
Slope	0.2706	0.3963	0.3189	0.269	0.4591	0.4261	0.2534	0.2433	0.4253	0.1926	0.1804	0.157	0.3358	0.3692	0.3331	0.3216	0.2981	0.4167
Y-intercept	0.2642	0.01876	0.1181	0.2847	-0.1011	-0.07247	0.304	0.3125	-0.134	0.4256	0.4624	0.4105	0.1438	0.07117	0.1206	0.1811	0.2091	-0.03382
X-intercept	-0.9766	-0.04734	-0.3704	-1.059	0.2203	0.1701	-1.2	-1.284	0.3152	-2.21	-2.563	-2.615	-0.4281	-0.1928	-0.3622	-0.5632	-0.7013	0.08114
1/slope	3.696	2.524	3.136	3.717	2.178	2.347	3.946	4.11	2.351	5.193	5.543	6.37	2.978	2.709	3.002	3.11	3.355	2.4
Slope SE	0.02136	0.02533	0.02316	0.01654	0.02759	0.01839	0.0219	0.02552	0.03393	0.05614	0.03545	0.0168	0.01278	0.0214	0.02433	0.02353	0.044	0.06117
Y-intercept SE	0.04295	0.05216	0.04657	0.0342	0.05723	0.03568	0.04513	0.05284	0.0638	0.1162	0.07276	0.03397	0.02613	0.04293	0.04828	0.04931	0.08947	0.1258
percentage length			94			91			85			88			94			96

Supplemental Table 6. Thin filament length (descriptive statistics)
