# Supplementary material for: Comprehensive phenotypic characterization of an allelic series of zebrafish models of NEB-related nemaline myopathy
Source: Hum Mol Genet. 2024 Mar 17;33(12):1036–54. doi: 10.1093/hmg/ddae033 (PMC11153343; doi:10.1093/hmg/ddae033)
Supplement: Supplemental_Table_7_ddae033 [file supplemental_table_7_ddae033.pdf]

logFC	*neb*[15]	*neb*[11]	*neb*[hu28]	*neb*[34]	*neb*[21]	*neb*[30]
**neb**	-3.358	-3.439	-4.372	0.210	0.707	-0.048
**acta1b**	-1.934	-2.474	-3.109	-1.490	-2.047	-0.681
**klhl38b**	-0.036	-0.036	-2.779	-0.735	-0.281	-1.391
**ankrd1b**	0.296	-0.488	-2.659	0.083	0.203	0.032
**actc1b**	-0.899	-1.228	-2.162	-0.929	-1.198	-0.594
**acta1a**	-1.499	-1.584	-1.730	-1.527	0.275	-0.307
**tnnt1**	-0.707	-0.638	-1.340	-0.623	0.053	0.524
**tpma**	-0.071	-0.329	-1.230	-0.353	-0.304	-0.009
**lmod3**	0.174	-0.164	-0.488	-0.461	-0.037	0.049
**cfl2**	0.237	-0.154	-0.440	-0.290	-0.275	-0.042
**tpm2**	-0.530	-0.508	-0.388	-0.181	-0.154	0.093
**klhl41b**	0.258	-0.015	-0.360	-0.300	-0.206	0.020
**kbtbd13**	-0.410	-0.194	-0.301	-0.127		0.541
**tpm3**	-0.392	-0.367	-0.038	-0.064	-0.099	-0.039
**klhl40a**	0.669	0.193	0.201	-0.100	-0.176	0.328
**klhl41a**	0.172	0.131	0.663	0.247	-1.191	-0.226
**klhl40b**	1.193	0.111	1.292	0.059	0.124	0.340
**tmod1**	-0.409	-0.640	1.493	-0.271	-0.951	0.107

Supplemental Table 7. Nemaline myopathy genes
