# Supplementary material for: Comprehensive phenotypic characterization of an allelic series of zebrafish models of NEB-related nemaline myopathy
Source: Hum Mol Genet. 2024 Mar 17;33(12):1036–54. doi: 10.1093/hmg/ddae033 (PMC11153343; doi:10.1093/hmg/ddae033)
Supplement: Supplemental_Table_8_ddae033 [file supplemental_table_8_ddae033.pdf]

logFC	*neb*^{15}	*neb*^{11}	*neb*^{hu28}	*neb*^{34}	*neb*^{21}	*neb*^{30}
**acta2**	-1.006	-0.297	-1.169	-0.798	-0.158	-0.392
**actb1**	-0.170	0.052	0.502	0.142	-0.076	0.000
**actc1a**	-0.424	-0.576	-0.655	-0.381	-0.148	0.088
**actc1c**	-4.660	-4.045	-2.981	-2.327	-0.565	-0.526
**actn3a**	0.132	-0.123	-0.874	-0.118	-0.086	0.198
**atp2a2a**	-1.849	-1.659	-0.996	-0.685	-0.333	-0.849
**capza1a**	-0.019	0.173	0.592	0.070	0.401	-0.004
**capza1b**	-0.277	-0.485	-0.752	-0.510	-0.461	-0.091
**capza1b**	-0.277	-0.485	-0.752	-0.510	-0.461	-0.091
**capzb**	-0.390	-0.496	-0.423	-0.365	-0.775	-0.056
**desmb**	-1.506	-0.510	-1.688	-1.162	-0.987	-0.386
**jph1b**	-0.167	-0.179	-0.762	-0.158	-0.242	0.111
**kbtbd12**	0.101	0.061	-0.691	-0.234	-0.181	-0.001
**klhl31**	-0.523	-0.494	-1.264	-0.962	-0.765	-0.339
**lmod1a**	0.496	0.198	-0.256	-0.506	0.166	-0.266
**lmod1b**	0.195	-0.256	-0.410	0.047	1.056	1.142
**lmod2b**	0.071	0.542	-0.587	-0.418	0.204	0.068
**mustn1a**	-0.319	-0.330	-0.888	-0.299	-0.295	0.053
**mustn1b**	3.043	1.950	1.360	1.175	2.238	1.554
**mybpc1**	-0.304	-0.432	-1.204	-0.167	0.155	0.384
**mybphb**	0.064	-0.278	-1.107	-0.269	-0.281	-0.007
**myh11a**	-1.111	-0.448	-0.909	-0.421	-0.605	-0.167
**myh7**	0.104	0.057	-0.656	-0.029	-0.207	0.066
**myha**	0.338	-0.857	-2.055	-1.225	-0.164	-0.165
**myhc4**	-0.226	-1.313	-1.263	-1.220	-1.160	-0.704
**myhz1.1**	-0.069	-0.465	-1.321	-0.422	-0.354	1.462
**myhz1.2**	-0.275	-0.725	-1.238	-0.340	-0.521	0.013
**myhz1.3**	-0.552	-1.112	-2.482	-0.903	-1.220	-0.429
**myhz2**	-0.373	-1.262	-1.748	-0.798	-0.931	-0.783
**myl1**	0.034	-0.327	-1.033	-0.610	-0.809	-0.105
**myl12.1**	0.040	0.179	0.811	0.173	-0.059	-0.125
**myl13**	-0.374	-0.609	-0.917	-0.238	0.008	-0.080
**mylk4a**	0.580	-0.068	-0.703	0.236	-1.269	0.291
**mylpfa**	-0.078	-0.667	-1.688	-0.356	-0.484	-0.120
**mylpfb**	0.147	-0.204	-1.290	-0.119	-0.312	-0.067
**mylz3**	-0.218	-0.734	-1.764	-0.464	-0.369	-0.109
**myo1b**	-0.995	-0.368	-0.845	-0.091	-0.763	-0.025
**myo7bb**	-1.606	-0.354	-1.332	-0.115	-2.245	-0.144
**myod1**	1.218	0.440	-0.869	0.646	0.439	0.481
**myom1a**	0.127	-0.213	-0.695	-0.200	-0.125	0.142
**myom2a**	0.105	-0.152	-0.717	-0.247	-0.187	0.084
**myoz1a**	-0.184	-0.309	-1.003	-0.204	-0.491	0.021
**myoz1b**	0.413	0.160	-0.854	0.005	0.107	0.249
**myoz2a**	0.166	0.036	-1.124	-0.117	0.007	0.422
**palld**	0.057	-0.062	0.073	-0.492	-0.201	-0.302
**pbld2**	-0.732	-0.845	-1.313	-0.642	-0.673	-0.271
**pvalb1**	0.135	-0.276	-1.695	-0.230	0.360	0.249
**pvalb2**	0.128	-0.272	-1.478	-0.282	-0.199	-0.012
**pvalb3**	-0.698	-1.101	-1.353	-0.613	-0.375	0.739
**pvalb4**	-0.529	-0.631	-1.634	-0.379	-0.082	0.043
**ryr3**	0.142	-0.213	-1.016	-0.263	0.136	0.287
**tmod2**	-0.110	-0.192	0.912	-0.064	-0.287	-0.156
**tmod4**	0.284	0.130	-0.934	-0.013	-0.068	0.048
**tnnc2**	-0.064	-0.394	-1.188	-0.315	-0.255	0.037
**tnni1b**	0.429	0.330	0.670	0.455	0.552	0.773
**tnni1c**	-1.304	-0.907	-0.580	-0.439	-0.334	-0.207
**tnni1d**	0.423	0.412	-1.254	0.561	0.000	0.583
**tnni2a.2**	-0.968		-1.217	-1.052		1.263
**tnni2a.3**	-0.839		-1.724	-0.577	0.505	1.692
**tnni2a.4**	-0.029	-0.364	-1.180	-0.377	-0.312	-0.063
**tnni2b.1**	0.282	0.446	0.846	0.404	0.674	0.760
**tnni2b.2**	-0.053	0.557	-0.380	0.076	0.492	0.196
**tnni4a**	0.569	0.473	0.215	0.526	-0.029	0.313
**tnni4b.1**	-3.747	-1.566	-3.437	-1.294	-0.287	-1.114
**tnni4b.2**	-0.395	-0.545	-1.063	-0.234	0.189	-0.041
**tnnt2b**	0.262	0.006	-1.107	0.191	-0.003	0.645
**tnnt2c**	1.392	0.731	1.190	1.498	-0.174	0.974
**tnnt2d**	-0.903	-0.751	-1.011	-0.289	-0.463	-0.272
**tnnt2e**	0.173	-0.027	0.120	0.045	0.179	0.061
**tnnt3a**	-0.035	-0.485	-0.023	-0.293	0.106	0.304
**tnnt3b**	-0.014	-0.305	-1.209	-0.264	-0.289	0.101
**tpm1**	-0.561	-0.340	-0.360	-0.372	-0.128	0.081
**tpm4a**	-0.215	-0.303	0.501	-0.018	-0.312	0.402
**tpm4b**	0.204	-0.322	0.595	0.318	-0.131	0.759
**trim109**	0.378	-0.329	-0.534	-0.391	0.410	-0.076
**trim63a**	0.018	-0.105	-1.818	-0.894	-0.241	-1.271
**trim63b**	0.527	0.087	-1.333	-0.524	0.469	-0.511
**ttn.1**	0.090	-0.466	-1.596	0.114	-0.289	0.309
**ttn.2**	0.170	-0.462	-1.530	0.174	0.261	0.348
**twf2a**	0.729	0.373	-1.126	-0.105	0.086	-0.309
**ucmab**	-1.643	-0.781	-1.165	-1.201	0.641	-1.539
**wasb**	0.205	-0.415	-1.629	-0.400	0.211	-0.325
**xirp1**	1.070	0.364	0.930	0.019	1.157	0.870
**xirp2a**	0.439	-0.093	0.485	-0.020	0.203	0.526
**xirp2b**	-0.265	-0.240	0.445	-0.509	-0.970	0.461
**zgc:153867**	0.015	0.160	0.909	0.162	0.248	0.156
**zyx**	-0.717	-0.172	1.389	-0.311	0.015	-0.677

Supplemental Table 8. Muscle relevant genes
