# Supplementary material for: Comprehensive phenotypic characterization of an allelic series of zebrafish models of NEB-related nemaline myopathy
Source: Hum Mol Genet. 2024 Mar 17;33(12):1036–54. doi: 10.1093/hmg/ddae033 (PMC11153343; doi:10.1093/hmg/ddae033)
Supplement: Fabian_et_al_Supplemental_Material_accepted_ddae033 [file fabian_et_al_supplemental_material_accepted_ddae033.pdf]

Supplementary Figure 1

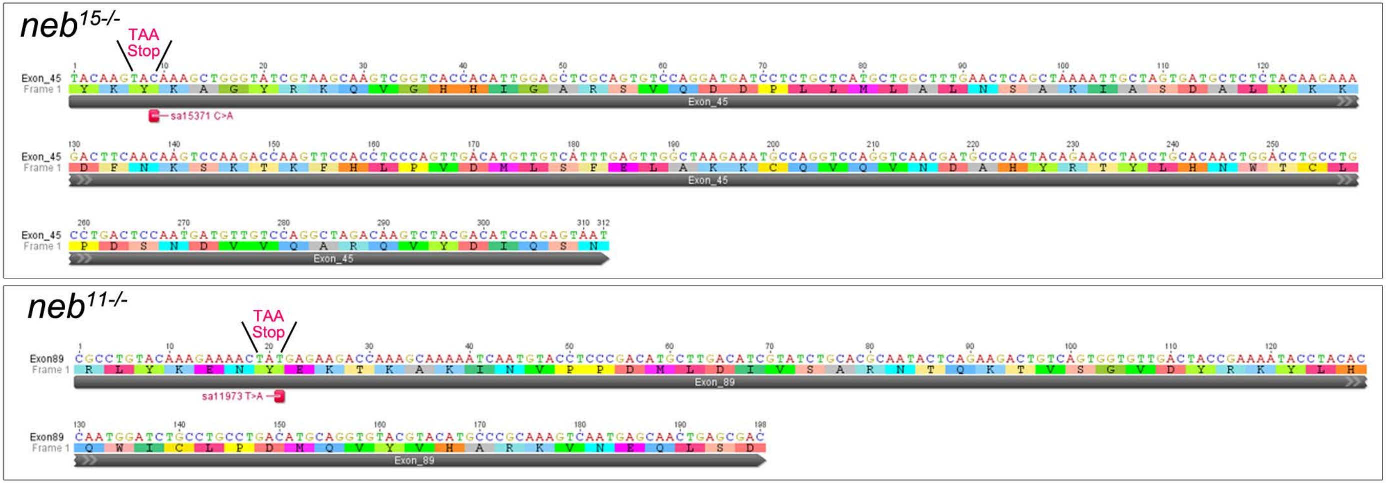

Supplementary Figure 2

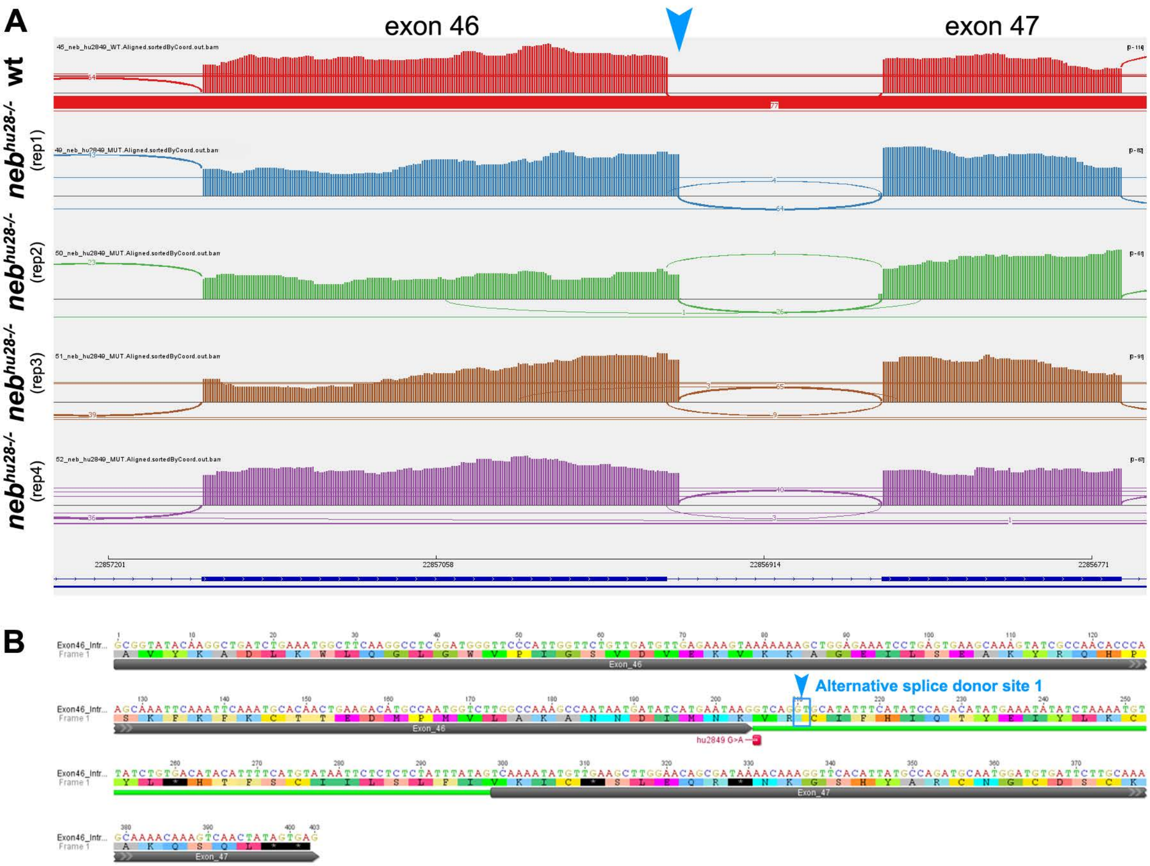

Supplementary Figure 3

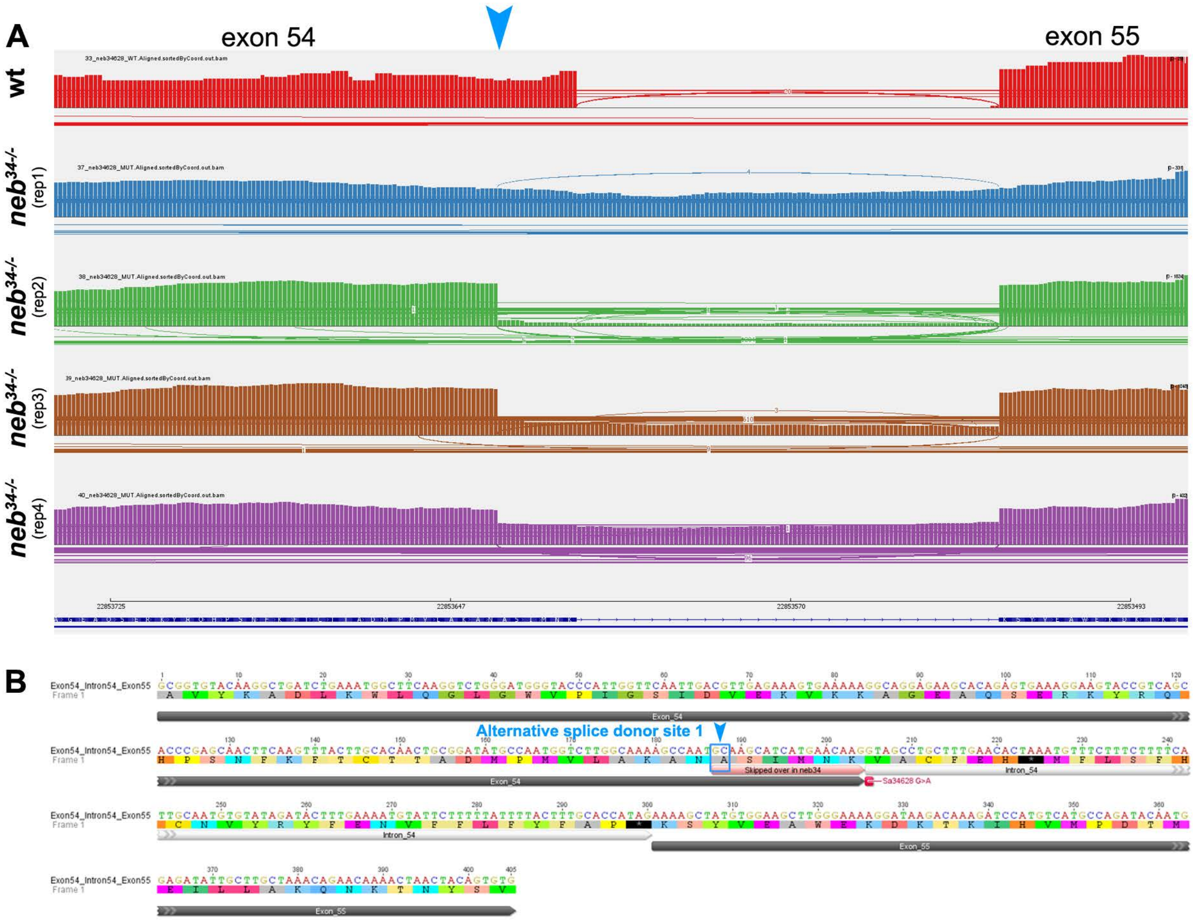

Supplementary Figure 4

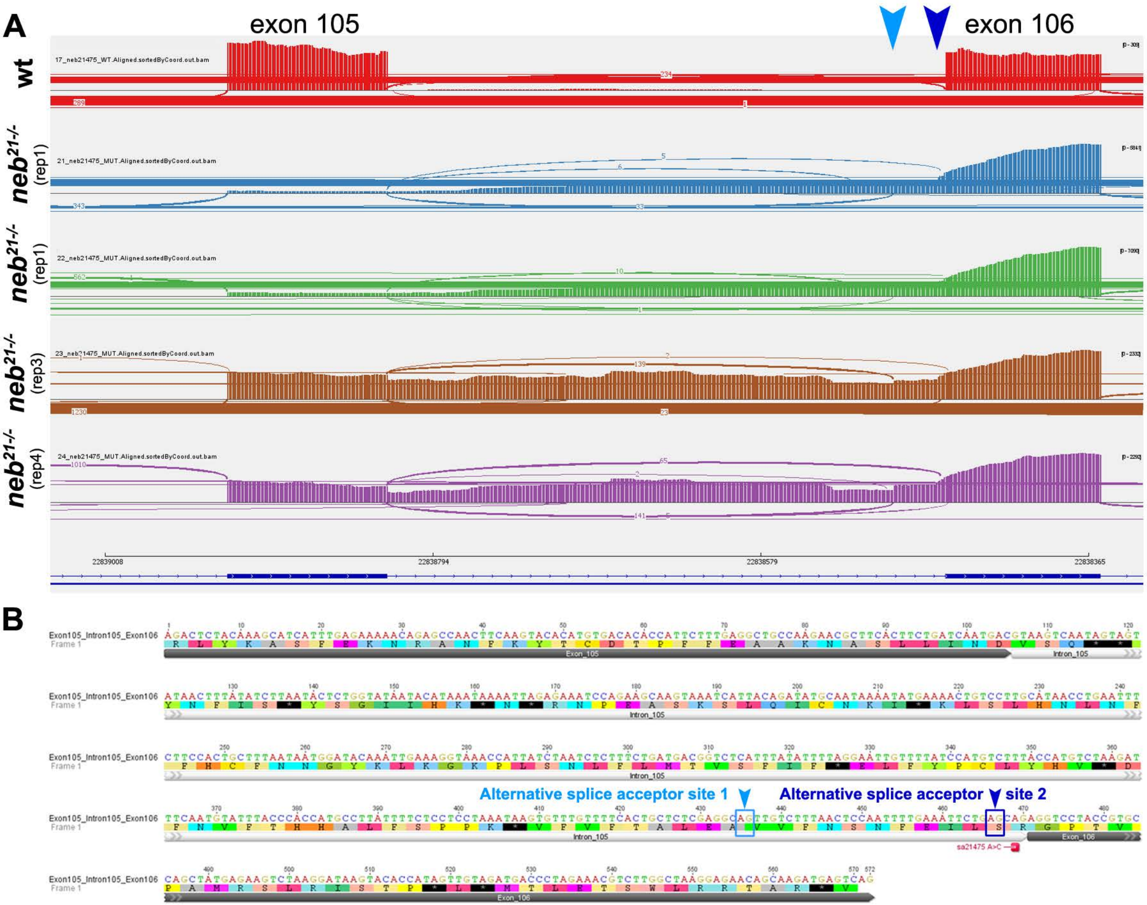

Supplementary Figure 5

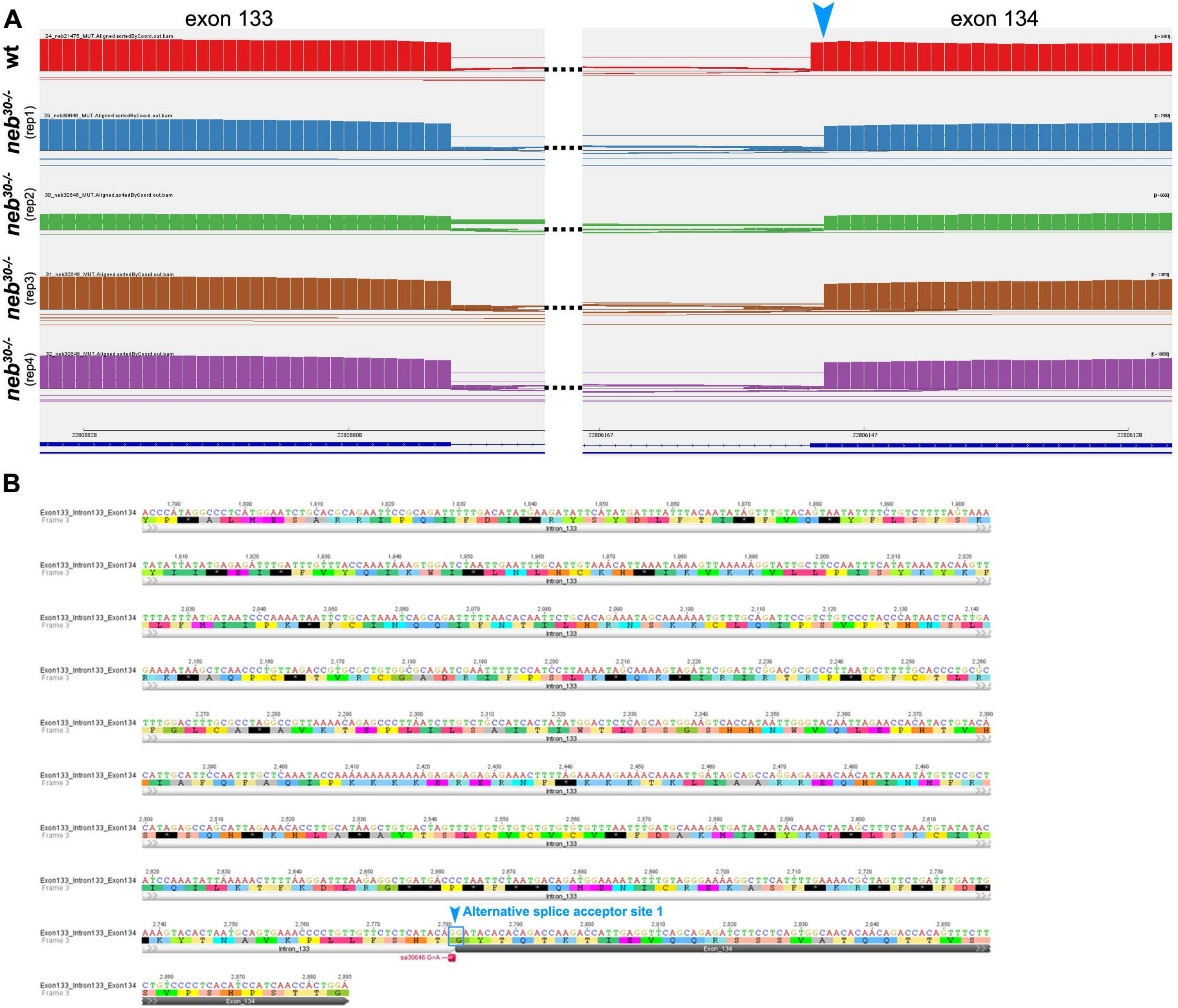

Suppl Fig 6. kinetics @ optimal length

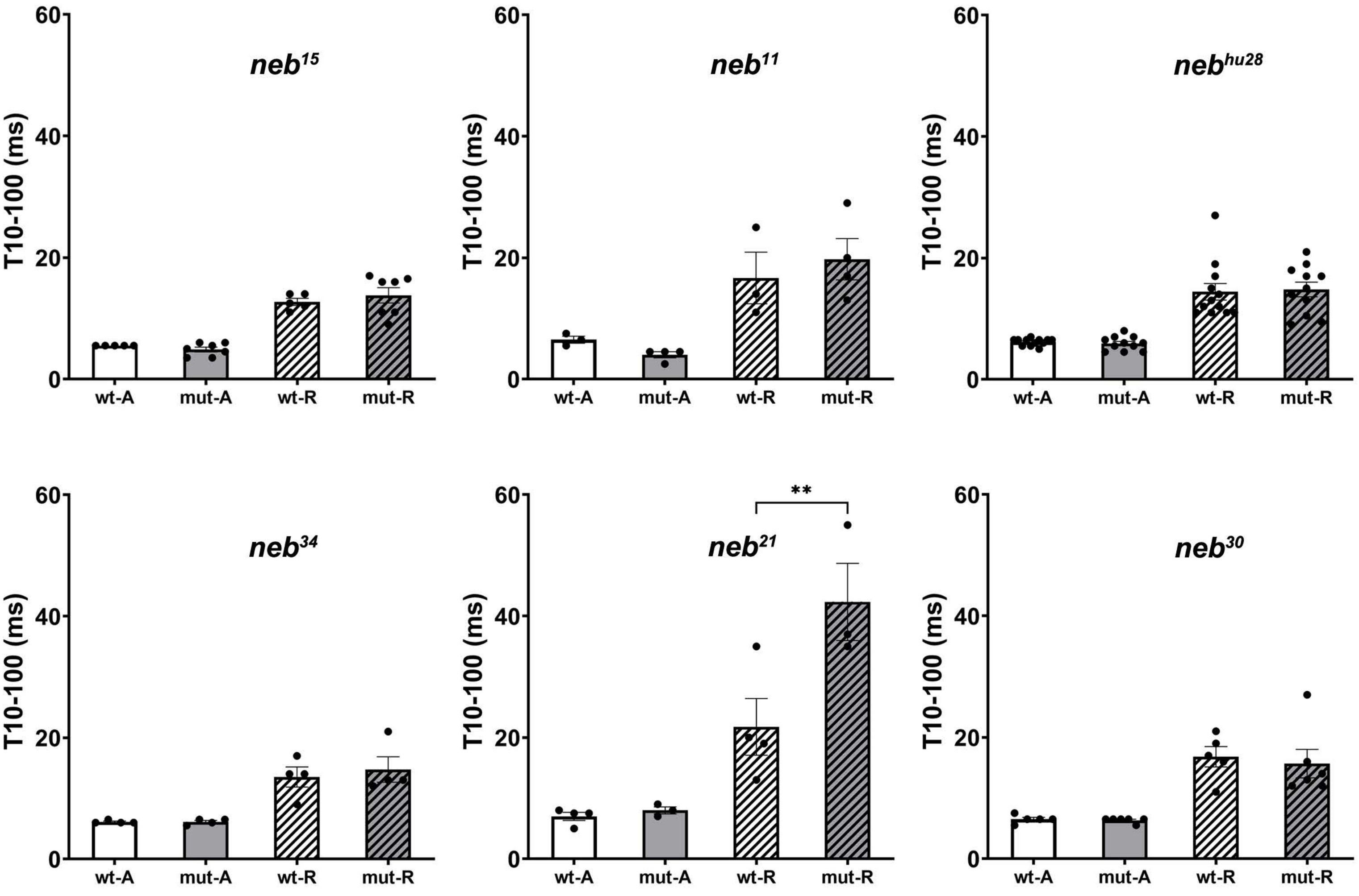

A = Activation kinetics at optimal sarcomere length
R = Relaxation kinetics at optimal sarcomere length

Suppl Fig 7 - muscle genes

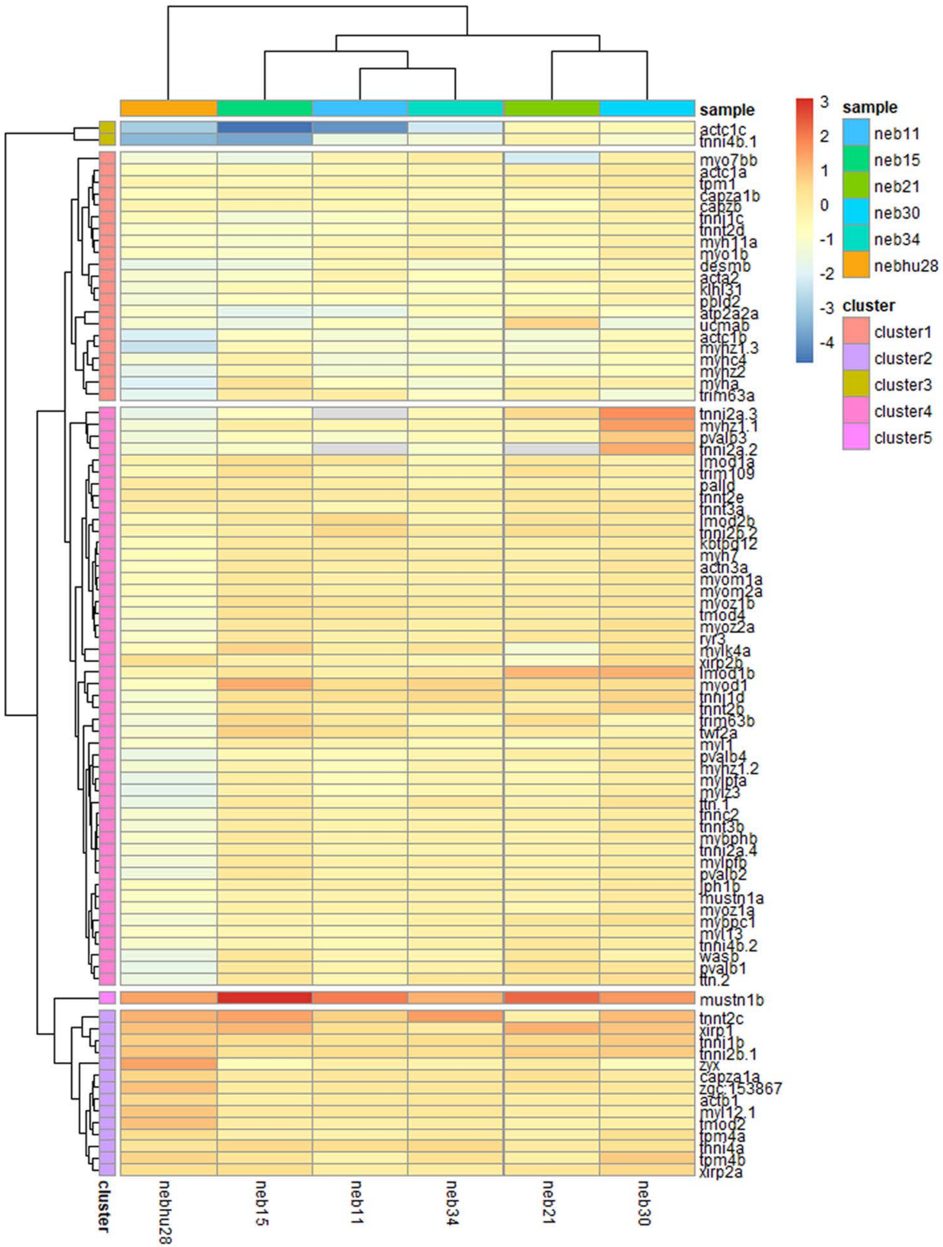

SUPPLEMENTAL FIGURE LEGENDS

Supplemental Figure 1. DNA sequences and predicted aminoacid sequences of *neb* nonsense mutants. Point mutations in neb^{15} (exon 45) and neb^{11} (exon 89) result in formation of a premature stop codon.

Alt text:
decorative

Supplemental Figure 2. Alternative splicing in neb^{hu28}. A. Sashimi plot generated with Integrative Genome Viewer (IGV) depicting expression of exons 46-47 and splice junctions from aligned RNA-seq data in one of the wt (red) and four neb^{hu28} mutant replicates (blue, green, brown, purple), which harbour a point mutation in the splice donor site of intron 46 (C>T). One alternative splicing event is noticeable in the mutant sashimi plots: use of alternate splice donor site located in exon 46. **B**. DNA and predicted aminoacid sequences of neb^{hu28} mutant. Splicing analysis identified an alternative splice donor site (blue arrowhead) located 5 bp downstream of the regular splice donor site, in intron 46.

Alt text:
decorative

Supplemental Figure 3. Alternative splicing in neb^{34}. A. Sashimi plot generated with Integrative Genome Viewer (IGV) depicting expression of exons 54-55 and splice junctions from aligned RNA-seq data in one of the wt (red) and four neb^{34} mutant replicates (blue, green, brown, purple), which harbour a point mutation in the splice donor site of intron 54 (C>T). Two alternative splicing events are noticeable in the mutant sashimi plots: full intron inclusion and use of alternate splice donor site located in exon 54. **B**. DNA and predicted aminoacid (aa) sequences of neb^{34} mutant. Splicing analysis identified an alternative splice donor site (blue arrowhead) located 18 bp upstream of the regular splice donor site.

Alt text:
decorative

Supplemental Figure 4. Alternative splicing in *neb*^{21}. A. Sashimi plot generated with Integrative Genome Viewer (IGV) depicting expression of exons 105-106 and splice junctions from aligned RNA-seq data in one of the wt (red) and four *neb*^{21} mutant replicates (blue, green, brown, purple), which harbour a point mutation in the splice acceptor site of intron 105 (T>G). Three alternative splicing events are noticeable in the mutant sashimi plots: full intron inclusion and use of two alternate splice acceptor sites located in intron 105. **B**. DNA and predicted aminoacid sequences of *neb*^{21} mutant. Splicing analysis identified two alternative splice acceptor sites (light and dark blue arrowheads) located in the intronic region.

Alt text:
decorative

Supplemental Figure 5. Alternative splicing in *neb*^{30}. A. Sashimi plot generated with Integrative Genome Viewer (IGV) depicting expression of exons 133-134 and splice junctions from aligned RNA-seq data in one of the wt (red) and four *neb*^{30} mutant replicates (blue, green, brown, purple), which harbour a point mutation in the splice acceptor site of intron 133 (C>T). Two alternative splicing events are noticeable in the mutant sashimi plots: full intron inclusion and use of an alternate splice acceptor site located in exon 134. **B**. DNA and predicted aminoacid sequences of *neb*^{30} mutant. Splicing analysis identified an alternative splice acceptor site (blue arrowhead) located in exon 134.

Alt text:
decorative

Supplemental Figure 6. Kinetics of muscle contraction in *neb* mutants. Graphs illustrating activation (left column) and relaxation (right column) kinetics of 1Hz

stimulated contractions in *neb* mutants (mean ± SEM). There was no significant difference in the rate of force development or relaxation in any of six *neb* mutants from this study. Asterisks indicate p-values. (* ≤ 0.05; ** ≤ 0.01; *** ≤ 0.001; **** ≤ 0.0001). See Suppl. Table 5 for measurements and statistics.

Alt text:
There is no significant difference in activation and relaxation kinetics at optimal sarcomere length between neb mutants and their wild-type siblings. refer to supplemental table five for measurements and statistics.

Supplemental Figure 7. Expression of muscle-relevant genes in *neb* mutants. Dendrogram and heatmap illustrating expression of several muscle-relevant genes in nebulin mutants. Noticeable, *mustn1b* is the only gene from this selected group that is upregulated in all the neb mutants investigated in this study. *actc1a* and *tnni4b* are drastically downregulated in most of the mutants and clustering together.

Alt text:
decorative

		average intensity		
		wt	neb^{34}	neb^{30}
myofiber 1	sarcomere 1	98.56	63.04	76.45
	sarcomere 2	93.68	74.53	81.94
	sarcomere 3	92.51	57.96	83.18
	sarcomere 4	102.43	56.73	84.25
	sarcomere 5	95.59	53.5	79.74
	sarcomere 6	82.39	56.92	90.53
	sarcomere 7	93.1	48.27	88.34
	sarcomere 8	94.42	66.82	85.88
myofiber 2	sarcomere 1	99.48	28.43	76.55
	sarcomere 2	101.39	28.81	82.32
	sarcomere 3	100.7	31.13	78.41
	sarcomere 4	100.69	30.44	83.65
	sarcomere 5	102.67	40.1	78.73
	sarcomere 6	102.37	38.77	68.66
	sarcomere 7	99.62	46.18	75.8
	sarcomere 8	97.38	50	80.06
myofiber 3	sarcomere 1	81.45	69.41	79.83
	sarcomere 2	84.21	55.46	76.65
	sarcomere 3	87.75	60.86	81.69
	sarcomere 4	86.28	52.61	74.99
	sarcomere 5	84.55	64.06	81.77
	sarcomere 6	83.18	67.29	79.36
	sarcomere 7	81.97	56.42	89.55
	sarcomere 8	87.36	65.69	88.94
myofiber 4	sarcomere 1	98.25	66.75	95.15
	sarcomere 2	98.25	72.83	89.8
	sarcomere 3	101.19	63.06	90.68
	sarcomere 4	101.97	59.49	98.87
	sarcomere 5	91.08	52.57	83.2
	sarcomere 6	94.13	49.74	96.87
	sarcomere 7	86.66	64.24	94.22
	sarcomere 8	101.59	66.88	87.53
myofiber 5	sarcomere 1	64.91	60.81	63.64
	sarcomere 2	75.93	57.98	57.92
	sarcomere 3	87.11	50.26	64.47
	sarcomere 4	87.89	63.28	68.14
	sarcomere 5	84.3	61.16	71.43
	sarcomere 6	81.06	52.73	67.14
	sarcomere 7	81.36	63.37	68.84
	sarcomere 8	80.22	56.03	69.67
myofiber 6	sarcomere 1	102.59	51.53	102.66
	sarcomere 2	101.59	50.6	102.64
	sarcomere 3	96.81	41.33	102.67
	sarcomere 4	101.62	33.43	102.67
	sarcomere 5	100.74	36.89	102.67
	sarcomere 6	101.16	48.13	102.67
	sarcomere 7	98.55	46.92	102.67
	sarcomere 8	98.7	41.62	102.67
myofiber 7	sarcomere 1	99.89	48.47	102.67
	sarcomere 2	97.5	52.08	102.67
	sarcomere 3	98.96	51.91	102.67
	sarcomere 4	98.67	51.74	102.67
	sarcomere 5	102.54	51.56	102.67
	sarcomere 6	95.51	39.2	102.67
	sarcomere 7	96.45	46.14	102.67
	sarcomere 8	97.93	44.58	102.67
n		56	56	56
Mean		93.55	52.87	86.74
SEM		1.139	1.509	1.736
%			56.50%	92.70%

Supplemental Table 1. Average fluorescence intensity measurements of anti Neb-N antibody staining in myofiber preparations and descriptive statistics

		wt		neb15		neb11		neb34		neb21		neb30	
		n (pixels)	average intensity	n (pixels)	average intensity	n (pixels)	average intensity	n (pixels)	average intensity	n (pixels)	average intensity	n (pixels)	average intensity
**measurement 1**	**fish 1**	148	254.40	145	156.28	184	174.32	138	165.56	168	136.39	117	255.00
	**fish 2**	147	254.92	156	97.59	145	113.32	175	210.39	183	81.21	121	251.45
	**fish 3**	135	255.00	146	124.93	122	35.39	160	153.63	147	154.28	117	253.10
	**fish 4**	124	255.00	131	175.58	147	100.64	157	174.27	147	43.17	124	255.00
	**fish 5**	135	255.00	150	159.04	121	120.42	164	186.36	154	58.03	142	255.00
	**fish 6**	169	250.24										
	**fish 7**	140	255.00										
	**fish 8**	158	255.00										
**measurement 2**	**fish 1**	142	254.97	143	194.88	156	233.27	144	201.54	121	187.05	255.00	255.00
	**fish 2**	122	255.00	135	181.41	132	187.08	145	234.83	180	161.42	255.00	255.00
	**fish 3**	111	254.94	132	189.16	99	61.12	137	221.50	136	231.19	254.77	254.77
	**fish 4**	120	254.95	117	234.72	155	44.15	132	206.52	125	85.69	255.00	255.00
	**fish 5**	110	255.00	145	216.88	132	154.34	148	231.09	150	153.58	255.00	255.00
	**fish 6**	153	254.62										
	**fish 7**	123	255.00										
	**fish 8**	151	255.00										
**n**		16		10		10		10		10		10	
**Mean**		254.6		122.4		173		198.6		129.2		254.4	
**SEM**		0.2955		20.6		12.92		8.793		19.07		0.3806	

Supplemental Table 2. Average birefringence intensity measurements (grey scale value) and descriptive statistics.

zebrafish line	neb^{15}			neb^{11}			neb^{34}			neb^{21}			neb^{30}		
**recorded values**															
**time spent moving**	**wt**	**het**	**mut**	**wt**	**het**	**mut**	**wt**	**het**	**mut**	**wt**	**het**	**mut**	**wt**	**het**	**mut**
**n**	11	14	12	9	24	21	12	11	23	9	17	8	13	19	16
**mean**	145.50	142.50	92.00	130.33	117.07	93.47	153.70	170.70	131.90	164.10	150.30	129.20	140.20	114.90	137.50
**SEM**	10.77	8.75	4.45	11.29	6.00	5.55	11.39	10.64	7.13	9.18	8.32	12.57	13.16	8.43	9.45
**distance travelled**	**wt**	**het**	**mut**	**wt**	**het**	**mut**	**wt**	**het**	**mut**	**wt**	**het**	**mut**	**wt**	**het**	**mut**
**n**	11	14	12	9	24	21	12	11	23	9	17	8	13	19	16
**mean**	386.60	386.20	167.80	360.50	337.80	230.00	428.60	447.00	304.00	367.90	389.80	300.90	347.10	301.90	353.40
**SEM**	33.34	23.85	9.48	30.11	15.24	13.49	23.78	25.77	16.21	27.36	22.01	39.01	32.59	28.66	23.79
**% change**			43.40			63.80			70.93			81.79			101.82
**average speed**	**wt**	**het**	**mut**	**wt**	**het**	**mut**	**wt**	**het**	**mut**	**wt**	**het**	**mut**	**wt**	**het**	**mut**
**n**	11	14	12	9	24	21	12	11	23	9	17	8	13	19	16
**mean**	2.64	2.75	1.84	2.85	2.98	2.50	2.92	2.66	2.38	2.27	2.65	2.29	2.53	2.64	2.62
**SEM**	0.12	0.14	0.08	0.19	0.14	0.12	0.21	0.12	0.15	0.17	0.14	0.18	0.17	0.22	0.14
**normalized values**															
**time spent moving**	**wt**	**het**	**mut**	**wt**	**het**	**mut**	**wt**	**het**	**mut**	**wt**	**het**	**mut**	**wt**	**het**	**mut**
**n**	11	14	12	9	24	21	12	11	23	9	17	8	13	19	16
**mean**	1.00	0.98	0.63	1.00	0.90	0.72	1.00	1.11	0.86	1.00	0.92	0.79	1.00	0.82	0.98
**SEM**	0.07	0.06	0.03	0.09	0.05	0.04	0.07	0.07	0.05	0.06	0.05	0.08	0.09	0.06	0.07
**distance travelled**	**wt**	**het**	**mut**	**wt**	**het**	**mut**	**wt**	**het**	**mut**	**wt**	**het**	**mut**	**wt**	**het**	**mut**
**n**	11	14	12	9	24	21	12	11	23	9	17	8	13	19	16
**mean**	1.00	1.00	0.43	1.00	0.94	0.64	1.00	1.04	0.71	1.00	1.06	0.82	1.00	0.87	1.02
**SEM**	0.09	0.06	0.02	0.08	0.04	0.04	0.06	0.06	0.04	0.07	0.06	0.11	0.09	0.08	0.07
**average speed**	**wt**	**het**	**mut**	**wt**	**het**	**mut**	**wt**	**het**	**mut**	**wt**	**het**	**mut**	**wt**	**het**	**mut**
**n**	11	14	12	9	24	21	12	11	23	9	17	8	13	19	16
**mean**	1.00	1.04	0.70	1.00	1.05	0.88	1.00	0.91	0.81	1.00	1.17	1.01	1.00	1.05	1.04
**SEM**	0.05	0.05	0.03	0.07	0.05	0.04	0.07	0.04	0.05	0.08	0.06	0.08	0.07	0.09	0.06

Supplemental Table 3. Descriptive statistics for swim assay measurements (recorded and normalized values for time spent moving, distance travelled, average speed)

Frequency	1Hz	5 Hz	10 Hz	20 Hz	40 Hz	60 Hz	80 Hz	100 Hz	150 Hz	200 Hz	1Hz	5 Hz	10 Hz	20 Hz	40 Hz	60 Hz	80 Hz	100 Hz	150 Hz	200 Hz
	neb15 wt sib										neb15 mut									
	8.45	8.17	7.98	7.88	8.02	7.58	7.25	7.42	11.96	16.27	5.52	5.25	5.30	5.38	5.44	5.34	7.94	9.94	13.62	15.40
	34.62	33.69	33.79	32.31	33.30	32.67	31.74	30.85	37.29	47.45	1.81	1.84	1.89	1.86	2.84	4.34	6.32	8.10	12.68	13.14
	16.47	16.24	16.70	16.45	17.82	17.48	17.61	17.30	28.55	37.23	1.84	1.81	1.58	1.83	2.63	4.17	6.68	7.70	9.12	10.38
	6.04	6.27	6.41	6.43	6.63	6.40	6.94	10.15	18.33	24.37	1.87	2.01	1.90	2.04	2.07	3.05	5.32	6.86	9.19	10.46
	13.43	12.89	13.15	13.39	13.24	12.81	12.95	15.76	30.95	40.40	2.50	2.42	2.64	2.76	3.49	5.30	8.39	11.30	14.95	16.88
											0.79	1.08	1.15	0.94	1.22	1.65	2.37	3.12	4.60	5.14
**n**	5	5	5	5	5	5	5	5	5	5	7	7	7	7	7	7	7	7	7	7
**Mean**	15.80	15.45	15.61	15.29	15.80	15.39	15.30	16.30	25.42	33.15	2.18	2.21	2.21	2.26	2.67	3.57	5.51	7.00	9.65	10.78
**SEM**	11.29	10.92	10.96	10.35	10.74	10.63	10.20	9.08	10.16	12.61	1.59	1.42	1.47	1.51	1.51	1.68	2.63	3.39	4.44	4.85
**SD**	5.05	4.88	4.90	4.63	4.80	4.75	4.56	4.06	4.54	5.64	0.60	0.54	0.55	0.57	0.57	0.63	0.99	1.28	1.68	1.83
											percentage force at 200 Hz (compared to wt siblings)									33
	neb11 wt sib										neb11 mut									
	31.11	31.01	29.15	27.40	27.58	25.28	24.70	23.68	25.65	26.09	2.98	2.99	2.99	2.93	2.90	2.83	2.62	3.02	5.05	5.94
	24.05	24.04	23.45	23.66	22.81	22.76	23.78	24.63	25.95	26.68	0.96	0.80	0.88	0.79	0.86	0.94	0.84	0.92	1.03	1.57
	32.93	32.74	30.62	31.66	29.72	29.04	27.95	26.77	30.17	30.43	4.81	4.87	4.71	4.73	4.82	5.73	5.70	5.62	6.30	5.94
											5.22	5.21	4.93	4.95	5.01	5.63	5.82	5.95	6.93	6.59
**n**	3	3	3	3	3	3	3	3	3	3	4	4	4	4	4	4	4	4	4	4
**Mean**	29.36	29.26	27.74	27.57	26.70	25.69	25.47	25.03	27.26	27.73	3.49	3.47	3.38	3.35	3.40	3.79	3.75	3.88	4.83	5.01
**SEM**	2.71	2.66	2.19	2.31	2.04	1.83	1.27	0.91	1.46	1.36	1.95	2.03	1.88	1.93	1.94	2.32	2.44	2.37	2.65	2.32
**SD**	4.69	4.61	3.79	4.00	3.54	3.16	2.19	1.58	2.53	2.35	0.97	1.02	0.94	0.97	0.97	1.16	1.22	1.18	1.33	1.16
											percentage force at 200 Hz (compared to wt siblings)									18
	nebhu28 wt sib										nebhu28 mut									
	20.14	18.52	17.76	17.61	16.21	16.15	16.10	14.98	18.61	21.15	2.04	2.12	2.04	1.79	1.72	1.75	1.84	1.97	2.91	3.03
	14.97	14.87	13.86	14.50	14.12	13.54	13.08	14.01	17.00	20.29	0.21	0.20	0.24	0.23	0.19	0.18	0.20	0.20	0.27	0.27
	16.89	15.70	16.30	15.96	15.43	15.21	15.77	16.79	18.44	20.52	0.88	0.82	0.84	0.78	0.81	0.74	0.66	0.75	0.88	1.04
	17.80	17.40	17.08	16.63	16.18	15.85	15.93	15.77	18.40	19.98	2.11	2.20	2.05	2.00	1.96	1.97	1.89	1.94	2.80	3.37
	11.60	11.71	11.48	11.80	10.31	9.00	8.66	7.79	8.33	9.58	0.54	0.63	0.64	0.65	0.91	1.24	1.62	2.07	1.95	2.29
	30.79	31.26	31.31	31.33	29.74	29.39	29.07	31.68	34.67	35.81	1.64	1.68	1.55	1.61	1.56	1.60	2.40	2.67	3.77	3.99
	28.84	27.89	28.38	31.06	25.58	26.38	26.27	26.27	32.97	28.74	1.79	1.70	1.66	1.74	1.70	1.66	1.65	1.82	2.35	3.24
	29.66	30.30	28.89	28.11	26.55	25.65	23.85	23.94	27.26	27.89	0.53	0.53	0.54	0.52	0.54	0.48	0.61	0.72	1.10	1.08
	34.81	34.98	33.75	33.16	32.39	31.72	30.49	29.84	35.67	36.42	5.19	5.12	4.99	5.00	4.97	5.23	5.50	5.76	7.03	7.44
	40.97	43.84	43.77	43.77	42.36	37.46	36.34	38.70	44.69	40.82	4.37	4.54	4.33	4.50	4.67	5.05	4.99	5.18	6.00	6.35
											7.12	6.65	6.46	5.99	5.01	4.90	4.88	4.53	6.03	5.59
											0.34	0.37	0.30	0.39	0.37	0.37	0.38	0.39	0.47	0.50
**n**	10	10	10	10	10	10	10	10	10	10	12	12	12	12	12	12	12	12	12	12
**Mean**	24.65	24.64	24.26	24.39	22.89	22.03	21.56	21.98	25.60	26.12	2.23	2.21	2.14	2.10	2.03	2.10	2.22	2.33	2.96	3.18
**SEM**	9.67	10.51	10.47	10.50	10.10	9.31	8.92	9.64	11.20	9.59	2.20	2.11	2.04	1.97	1.81	1.88	1.88	1.88	2.31	2.34
**SD**	3.06	3.32	3.31	3.32	3.19	2.94	2.82	3.05	3.54	3.03	0.63	0.61	0.59	0.57	0.52	0.54	0.54	0.54	0.67	0.68
											percentage force at 200 Hz (compared to wt siblings)									12
	neb34 wt sib										neb34 mut									
	31.86	33.41	32.98	31.78	28.90	26.24	27.75	30.58	36.92	41.66	2.98	2.99	2.99	2.93	2.90	2.83	2.62	3.02	5.05	5.94
	42.32	39.69	39.27	37.10	35.02	34.56	34.67	35.11	40.83	42.17	13.82	13.97	13.62	13.98	13.85	14.11	14.70	17.00	19.74	19.99
	29.12	28.32	27.24	26.51	25.58	25.21	24.29	24.59	28.68	28.96	10.37	10.53	9.82	9.78	9.17	8.78	8.80	9.44	10.95	12.61
	6.49	10.66	11.92	11.97	11.10	9.15	8.91	8.89	10.59	13.18	8.53	8.52	8.58	8.36	8.32	7.83	8.03	8.80	10.83	11.54
											7.09	7.19	7.02	6.92	6.63	6.60	6.40	7.66	10.07	10.70
**n**	4	4	4	4	4	4	4	4	4	4	5	5	5	5	5	5	5	5	5	5
**Mean**	27.45	28.02	27.85	26.84	25.15	23.79	23.90	24.79	29.25	31.49	8.56	8.64	8.41	8.39	8.18	8.03	8.11	9.18	11.33	12.16
**SEM**	15.09	12.47	11.70	10.82	10.15	10.62	10.89	11.44	13.43	13.65	4.01	4.07	3.89	4.03	3.98	4.08	4.39	5.04	5.30	5.07
**SD**	7.54	6.24	5.85	5.41	5.07	5.31	5.44	5.72	6.72	6.83	1.79	1.82	1.74	1.80	1.78	1.83	1.96	2.25	2.37	2.27
											percentage force at 200 Hz (compared to wt siblings)									39
	neb21 wt sib										neb21 mut									
	41.24	38.47	38.18	37.24	37.59	35.10	35.90	35.72	43.92	46.58	1.95	2.36	2.54	3.20	4.45	5.36	6.78	7.58	8.52	8.28
	6.15	6.04	5.91	6.67	9.58	11.45	13.84	15.04	17.99	17.89	7.20	7.34	8.17	10.67	14.96	18.21	23.53	29.51	31.74	31.19
	35.15	36.22	36.22	37.25	34.96	36.07	47.45	57.59	75.95	75.45	3.77	4.06	5.00	6.31	8.20	10.47	13.45	14.76	16.88	15.76
	23.06	22.81	22.16	21.85	22.33	22.52	24.57	29.44	29.93	31.62	7.96	7.96	7.65	8.24	10.32	13.08	16.76	17.48	20.52	20.25
**n**	4	4	4	4	4	4	4	4	4	4	4	4	4	4	4	4	4	4	4	4
**Mean**	26.40	25.89	25.62	25.75	26.11	26.28	30.44	34.45	41.95	42.88	5.22	5.43	5.84	7.11	9.48	11.78	15.13	17.33	19.42	18.87
**SEM**	15.47	14.93	14.95	14.64	12.88	11.66	14.48	17.69	25.02	24.67	2.84	2.67	2.60	3.15	4.39	5.35	6.97	9.13	9.63	9.58
**SD**	7.74	7.46	7.48	7.32	6.44	5.83	7.24	8.85	12.51	12.34	1.42	1.33	1.30	1.58	2.19	2.68	3.49	4.56	4.82	4.79
											percentage force at 200 Hz (compared to wt siblings)									44
	neb30 wt sib										neb30 mut									
	8.62	8.28	7.24	6.90	6.77	6.30	5.64	5.62	4.94	5.64	19.27	18.95	18.55	17.76	17.49	16.66	16.41	15.73	16.40	16.92
	38.12	38.13	38.30	37.57	34.54	32.42	31.58	31.66	34.53	35.33	14.46	15.06	14.71	14.97	14.89	12.71	12.11	12.19	13.11	13.68
	34.19	34.66	34.96	35.75	33.50	31.73	35.13	37.08	39.06	42.70	7.44	7.10	7.20	7.03	7.03	6.88	6.77	6.71	8.19	9.44
	11.18	10.47	10.25	10.12	9.37	9.17	9.78	10.95	12.68	14.32	10.30	10.53	9.81	10.04	9.44	9.57	9.88	10.51	13.11	14.25
	44.11	42.38	41.09	39.14	37.80	36.99	37.52	40.50	43.39	45.86	31.01	29.76	27.17	24.63	21.61	22.75	23.71	25.61	27.70	25.53
											21.77	23.20	23.00	22.38	21.41	20.64	21.16	21.12	24.63	25.51
**n**	5	5	5	5	5	5	5	5	5	5	6	6	6	6	6	6	6	6	6	6
**Mean**	27.24	26.78	26.37	25.89	24.40	23.32	23.93	25.16	26.92	28.77	17.38	17.43	16.74	16.13	15.31	14.87	15.01	15.31	17.19	17.55
**SEM**	16.25	16.15	16.27	15.96	15.01	14.41	15.03	15.84	17.04	17.84	8.56	8.34	7.67	6.86	6.08	6.25	6.61	7.02	7.49	6.62
**SD**	7.27	7.22	7.28	7.14	6.71	6.44	6.72	7.08	7.62	7.98	3.49	3.40	3.13	2.80	2.48	2.55	2.70	2.87	3.06	2.70
											percentage force at 200 Hz (compared to wt siblings)									61

Supplemental Table 4. Force production measurements at optimal sarcomere length (recorded values and descriptive statistics)

		neb^{15}		neb^{11}		neb^{hu28}		neb^{34}		neb^{21}		neb^{30}	
		wt	mut	wt	mut	wt	mut	wt	mut	wt	mut	wt	mut
**Activation Kinetics**	**Slack**												
		5	5	4.5	4.5	5.5	5.5	6	5.5	8	7	4.5	5.5
		5.5	3.5	5.5	5.5	4.5	3.5	6	5.5	8	6	5.5	6.5
		5.5	6	5.5	4.5	5	1.5	6	6	6.5		5.5	5.5
		5.5	3.5		4.5	4.5	6.5	5.5	4.5	6		6.5	6.5
		5.5	6			4.5		6				5.5	7
			7.5			7							
			6.5										
	**n**	5	7	3	4	6	4	5	4	4	2	5	5
	**Mean**	5.4	5.429	5.167	4.75	5.167	4.25	5.9	5.375	7.125	6.5	5.5	6.2
	**SEM**	0.1	0.5714	0.3333	0.25	0.4014	1.109	0.1	0.3146	0.5154	0.5	0.3162	0.3
**Activation Kinetics**	**Optimal**												
		5.5	6	5.5	4.5	6.5	4.5	6	6.5	8	7	5.5	6.5
		5.5	3.5	7.5	2.5	6	7	6.5	6.5	5	9	6.5	6.5
		5.5	5.5	6.5	4.5	6.5	6.5	6	6	7.5	8	6.5	5.5
		5.5	3.5		4.5	5.5	7	6	5.5	7.5		6.5	6.5
		5.5	5			6.5	5.5					7.5	6.5
			4.5			6.5	6						6.5
			6			7	8						
						5.5	4.5						
						6	6						
						6.5	5.5						
						5	4.5						
						6.5							
	**n**	5	7	3	4	12	11	4	4	4	3	5	6
	**Mean**	5.5	4.857	6.5	4	6.167	5.909	6.125	6.125	7	8	6.5	6.333
	**SEM**	0	0.4041	0.5774	0.5	0.1667	0.3491	0.125	0.2394	0.677	0.5774	0.3162	0.1667
**Relaxation Kinetics**	**Slack**												
		6.5	13	9	9	8	13	10	11	10	26	7.5	12
		9	10.5	13	15	10.5	8	9	13	20	24	12	10
		9	11.5	10	10	9	8.5	11	11	13		11	9
		8	12.5		10	8	10	10	12	10		9	12
		11	9.5			8		8				13	12
			8			10							
			6.5										
	**n**	5	7	3	4	6	4	5	4	4	2	5	5
	**Mean**	8.7	10.21	10.67	11	8.917	9.875	9.6	11.75	13.25	25	10.5	11
	**SEM**	0.7348	0.8988	1.202	1.354	0.4549	1.125	0.5099	0.4787	2.358	1	1	0.6325
**Relaxation Kinetics**	**Optimal**												
		11	16	11	13	19	19	14	21	13	35	11	27
		12	16.5	25	17	14	17	17	13	35	37	16	14
		14	16	14	29	27	9	14	13	19	55	17	12
		12.5	17		20	15	21	9	12	20		21	13
		14	11			12	17					19	16
			9			11	18						12
			11			11	13						
						12	14						
						13	10.5						
						11	9.5						
						11	15						
						17							
	**n**	5	7	3	4	12	11	4	4	4	3	5	6
	**Mean**	12.7	13.79	16.67	19.75	14.42	14.82	13.5	14.75	21.75	42.33	16.8	15.67
	**SEM**	0.5831	1.253	4.256	3.4	1.368	1.207	1.658	2.097	4.679	6.36	1.685	2.348

Supplemental Table 5. Activation and relaxation kinetics (measurements and descriptive statistics)

zebrafish line	neb^{15}			neb^{11}			neb^{hu28}			neb^{34}			neb^{21}			neb^{30}		
	wt	het	mut	wt	het	mut	wt	het	mut	wt	het	mut	wt	het	mut	wt	het	mut
n	607	620	721	664	473	493	346	528	301	161	136	330	1262	793	625	549	149	117
mean	0.8075	0.8341	0.7588	0.8388	0.8529	0.7645	0.8192	0.8166	0.6928	0.8239	0.8308	0.7213	0.8301	0.8112	0.7816	0.8538	0.8161	0.8235
SEM	0.002098	0.002382	0.002455	0.002033	0.00324	0.00274	0.002791	0.002774	0.005091	0.004105	0.003927	0.003967	0.00121	0.001855	0.002274	0.002222	0.004387	0.006603
Slope	0.2706	0.3963	0.3189	0.269	0.4591	0.4261	0.2534	0.2433	0.4253	0.1926	0.1804	0.157	0.3358	0.3692	0.3331	0.3216	0.2981	0.4167
Y-intercept	0.2642	0.01876	0.1181	0.2847	-0.1011	-0.07247	0.304	0.3125	-0.134	0.4256	0.4624	0.4105	0.1438	0.07117	0.1206	0.1811	0.2091	-0.03382
X-intercept	-0.9766	-0.04734	-0.3704	-1.059	0.2203	0.1701	-1.2	-1.284	0.3152	-2.21	-2.563	-2.615	-0.4281	-0.1928	-0.3622	-0.5632	-0.7013	0.08114
1/slope	3.696	2.524	3.136	3.717	2.178	2.347	3.946	4.11	2.351	5.193	5.543	6.37	2.978	2.709	3.002	3.11	3.355	2.4
Slope SE	0.02136	0.02533	0.02316	0.01654	0.02759	0.01839	0.0219	0.02552	0.03393	0.05614	0.03545	0.0168	0.01278	0.0214	0.02433	0.02353	0.044	0.06117
Y-intercept SE	0.04295	0.05216	0.04657	0.0342	0.05723	0.03568	0.04513	0.05284	0.0638	0.1162	0.07276	0.03397	0.02613	0.04293	0.04828	0.04931	0.08947	0.1258
percentage length			94			91			85			88			94			96

Supplemental Table 6. Thin filament length (descriptive statistics)

logFC	*neb* [15]	*neb* [11]	*neb* [hu28]	*neb* [34]	*neb* [21]	*neb* [30]
**neb**	-3.358	-3.439	-4.372	0.210	0.707	-0.048
**acta1b**	-1.934	-2.474	-3.109	-1.490	-2.047	-0.681
**klhl38b**	-0.036	-0.036	-2.779	-0.735	-0.281	-1.391
**ankrd1b**	0.296	-0.488	-2.659	0.083	0.203	0.032
**actc1b**	-0.899	-1.228	-2.162	-0.929	-1.198	-0.594
**acta1a**	-1.499	-1.584	-1.730	-1.527	0.275	-0.307
**tnnt1**	-0.707	-0.638	-1.340	-0.623	0.053	0.524
**tpma**	-0.071	-0.329	-1.230	-0.353	-0.304	-0.009
**lmod3**	0.174	-0.164	-0.488	-0.461	-0.037	0.049
**cfl2**	0.237	-0.154	-0.440	-0.290	-0.275	-0.042
**tpm2**	-0.530	-0.508	-0.388	-0.181	-0.154	0.093
**klhl41b**	0.258	-0.015	-0.360	-0.300	-0.206	0.020
**kbtbd13**	-0.410	-0.194	-0.301	-0.127		0.541
**tpm3**	-0.392	-0.367	-0.038	-0.064	-0.099	-0.039
**klhl40a**	0.669	0.193	0.201	-0.100	-0.176	0.328
**klhl41a**	0.172	0.131	0.663	0.247	-1.191	-0.226
**klhl40b**	1.193	0.111	1.292	0.059	0.124	0.340
**tmod1**	-0.409	-0.640	1.493	-0.271	-0.951	0.107

Supplemental Table 7. Nemaline myopathy genes

logFC	*neb*^{15}	*neb*^{11}	*neb*^{hu28}	*neb*^{34}	*neb*^{21}	*neb*^{30}
**acta2**	-1.006	-0.297	-1.169	-0.798	-0.158	-0.392
**actb1**	-0.170	0.052	0.502	0.142	-0.076	0.000
**actc1a**	-0.424	-0.576	-0.655	-0.381	-0.148	0.088
**actc1c**	-4.660	-4.045	-2.981	-2.327	-0.565	-0.526
**actn3a**	0.132	-0.123	-0.874	-0.118	-0.086	0.198
**atp2a2a**	-1.849	-1.659	-0.996	-0.685	-0.333	-0.849
**capza1a**	-0.019	0.173	0.592	0.070	0.401	-0.004
**capza1b**	-0.277	-0.485	-0.752	-0.510	-0.461	-0.091
**capza1b**	-0.277	-0.485	-0.752	-0.510	-0.461	-0.091
**capzb**	-0.390	-0.496	-0.423	-0.365	-0.775	-0.056
**desmb**	-1.506	-0.510	-1.688	-1.162	-0.987	-0.386
**jph1b**	-0.167	-0.179	-0.762	-0.158	-0.242	0.111
**kbtbd12**	0.101	0.061	-0.691	-0.234	-0.181	-0.001
**klhl31**	-0.523	-0.494	-1.264	-0.962	-0.765	-0.339
**lmod1a**	0.496	0.198	-0.256	-0.506	0.166	-0.266
**lmod1b**	0.195	-0.256	-0.410	0.047	1.056	1.142
**lmod2b**	0.071	0.542	-0.587	-0.418	0.204	0.068
**mustn1a**	-0.319	-0.330	-0.888	-0.299	-0.295	0.053
**mustn1b**	3.043	1.950	1.360	1.175	2.238	1.554
**mybpc1**	-0.304	-0.432	-1.204	-0.167	0.155	0.384
**mybphb**	0.064	-0.278	-1.107	-0.269	-0.281	-0.007
**myh11a**	-1.111	-0.448	-0.909	-0.421	-0.605	-0.167
**myh7**	0.104	0.057	-0.656	-0.029	-0.207	0.066
**myha**	0.338	-0.857	-2.055	-1.225	-0.164	-0.165
**myhc4**	-0.226	-1.313	-1.263	-1.220	-1.160	-0.704
**myhz1.1**	-0.069	-0.465	-1.321	-0.422	-0.354	1.462
**myhz1.2**	-0.275	-0.725	-1.238	-0.340	-0.521	0.013
**myhz1.3**	-0.552	-1.112	-2.482	-0.903	-1.220	-0.429
**myhz2**	-0.373	-1.262	-1.748	-0.798	-0.931	-0.783
**myl1**	0.034	-0.327	-1.033	-0.610	-0.809	-0.105
**myl12.1**	0.040	0.179	0.811	0.173	-0.059	-0.125
**myl13**	-0.374	-0.609	-0.917	-0.238	0.008	-0.080
**mylk4a**	0.580	-0.068	-0.703	0.236	-1.269	0.291
**mylpfa**	-0.078	-0.667	-1.688	-0.356	-0.484	-0.120
**mylpfb**	0.147	-0.204	-1.290	-0.119	-0.312	-0.067
**mylz3**	-0.218	-0.734	-1.764	-0.464	-0.369	-0.109
**myo1b**	-0.995	-0.368	-0.845	-0.091	-0.763	-0.025
**myo7bb**	-1.606	-0.354	-1.332	-0.115	-2.245	-0.144
**myod1**	1.218	0.440	-0.869	0.646	0.439	0.481
**myom1a**	0.127	-0.213	-0.695	-0.200	-0.125	0.142
**myom2a**	0.105	-0.152	-0.717	-0.247	-0.187	0.084
**myoz1a**	-0.184	-0.309	-1.003	-0.204	-0.491	0.021
**myoz1b**	0.413	0.160	-0.854	0.005	0.107	0.249
**myoz2a**	0.166	0.036	-1.124	-0.117	0.007	0.422
**palld**	0.057	-0.062	0.073	-0.492	-0.201	-0.302
**pbld2**	-0.732	-0.845	-1.313	-0.642	-0.673	-0.271
**pvalb1**	0.135	-0.276	-1.695	-0.230	0.360	0.249
**pvalb2**	0.128	-0.272	-1.478	-0.282	-0.199	-0.012
**pvalb3**	-0.698	-1.101	-1.353	-0.613	-0.375	0.739
**pvalb4**	-0.529	-0.631	-1.634	-0.379	-0.082	0.043
**ryr3**	0.142	-0.213	-1.016	-0.263	0.136	0.287
**tmod2**	-0.110	-0.192	0.912	-0.064	-0.287	-0.156
**tmod4**	0.284	0.130	-0.934	-0.013	-0.068	0.048
**tnnc2**	-0.064	-0.394	-1.188	-0.315	-0.255	0.037
**tnni1b**	0.429	0.330	0.670	0.455	0.552	0.773
**tnni1c**	-1.304	-0.907	-0.580	-0.439	-0.334	-0.207
**tnni1d**	0.423	0.412	-1.254	0.561	0.000	0.583
**tnni2a.2**	-0.968		-1.217	-1.052		1.263
**tnni2a.3**	-0.839		-1.724	-0.577	0.505	1.692
**tnni2a.4**	-0.029	-0.364	-1.180	-0.377	-0.312	-0.063
**tnni2b.1**	0.282	0.446	0.846	0.404	0.674	0.760
**tnni2b.2**	-0.053	0.557	-0.380	0.076	0.492	0.196
**tnni4a**	0.569	0.473	0.215	0.526	-0.029	0.313
**tnni4b.1**	-3.747	-1.566	-3.437	-1.294	-0.287	-1.114
**tnni4b.2**	-0.395	-0.545	-1.063	-0.234	0.189	-0.041
**tnnt2b**	0.262	0.006	-1.107	0.191	-0.003	0.645
**tnnt2c**	1.392	0.731	1.190	1.498	-0.174	0.974
**tnnt2d**	-0.903	-0.751	-1.011	-0.289	-0.463	-0.272
**tnnt2e**	0.173	-0.027	0.120	0.045	0.179	0.061
**tnnt3a**	-0.035	-0.485	-0.023	-0.293	0.106	0.304
**tnnt3b**	-0.014	-0.305	-1.209	-0.264	-0.289	0.101
**tpm1**	-0.561	-0.340	-0.360	-0.372	-0.128	0.081
**tpm4a**	-0.215	-0.303	0.501	-0.018	-0.312	0.402
**tpm4b**	0.204	-0.322	0.595	0.318	-0.131	0.759
**trim109**	0.378	-0.329	-0.534	-0.391	0.410	-0.076
**trim63a**	0.018	-0.105	-1.818	-0.894	-0.241	-1.271
**trim63b**	0.527	0.087	-1.333	-0.524	0.469	-0.511
**ttn.1**	0.090	-0.466	-1.596	0.114	-0.289	0.309
**ttn.2**	0.170	-0.462	-1.530	0.174	0.261	0.348
**twf2a**	0.729	0.373	-1.126	-0.105	0.086	-0.309
**ucmab**	-1.643	-0.781	-1.165	-1.201	0.641	-1.539
**wasb**	0.205	-0.415	-1.629	-0.400	0.211	-0.325
**xirp1**	1.070	0.364	0.930	0.019	1.157	0.870
**xirp2a**	0.439	-0.093	0.485	-0.020	0.203	0.526
**xirp2b**	-0.265	-0.240	0.445	-0.509	-0.970	0.461
**zgc:153867**	0.015	0.160	0.909	0.162	0.248	0.156
**zyx**	-0.717	-0.172	1.389	-0.311	0.015	-0.677

Supplemental Table 8. Muscle relevant genes
